# Spiral Integration of Organic Synthesis and Instrumental Characterization Using Ferrocenyl Chalcones

**DOI:** 10.1021/acs.jchemed.5c01473

**Published:** 2026-07-21

**Authors:** Liz M. Díaz-Vázquez, Alejandro Burgos-Suazo, Ángela Cruz-Lugo, Jeremy Rivera-Sánchez, Raisa M. De Jesús Torres, Vanessa Álvarez-Carrillo, Ángel L. Morales-Cruz, Ingrid Montes-González

**Affiliations:** Department of Chemistry, 164191 University of Puerto Rico − Río Piedras Campus, San Juan, Puerto Rico 00925-2537, United States

**Keywords:** Upper-Division Undergraduate, Organic Chemistry, Analytical Chemistry, Laboratory Instruction, Instrumental Analysis, Materials Chemistry, Ferrocenyl Chalcones, Spiral Laboratory

## Abstract

Undergraduate chemistry curricula often present organic synthesis and instrumental analysis as disconnected experiences, limiting students’ ability to integrate structural, spectroscopic, and materials-focused concepts when interpreting authentic data. To address this problem, we implemented a spiral laboratory experience in a third-year Instrumental Analysis course at a Hispanic-serving research university (n ≈ 48) in which students revisited ferrocenyl chalcones synthesized in a prior organic chemistry course and analyzed them as “unknowns” using complementary chromatographic, spectroscopic, and thermal analysis techniques. This reintegration required students to draw on prior synthetic knowledge while developing new analytical reasoning skills by interpreting data collected across multiple analytical methods. A structured artificial intelligence (AI) prelaboratory component asked students to use targeted prompts to predict characteristic signatures, supporting conceptual preparation, and promoting critical evaluation of AI-generated chemical information. Assessment of laboratory reports and postactivity surveys revealed increased student confidence with analytical instrumentation, improved ability to integrate concepts across subdisciplines, and increased engagement with materials-oriented applications of organometallic compounds. This activity provides a replicable model for addressing curricular fragmentation by combining spiral integration, integrative analytical reasoning, and responsible AI use within the undergraduate chemistry laboratory.

## Graphical abstract

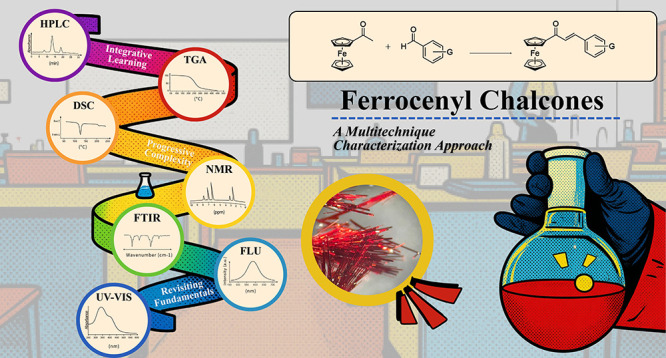

## Introduction

A persistent challenge in undergraduate chemistry education is the fragmentation of laboratory learning across subdisciplines. Organic Chemistry Laboratories (OCL) traditionally emphasize reaction execution and product isolation, while structural and physicochemical characterization is often treated superficially or deferred to later courses. As a result, students may complete experiments without fully connecting molecular structure to spectroscopic signatures, physicochemical behavior, or material properties. In materials chemistry, however, integration across structure, physicochemical properties, including spectroscopic, thermal behavior, and environmental interactions are essential to reveal how a material’s function emerges from the interplay of design, measurement, and interpretation. Therefore, laboratory experiences that intentionally reconnect synthesis with multi-instrumental characterization can help students move beyond procedural competence toward evidence-based chemical reasoning.

Chemical education research increasingly emphasizes laboratory environments that mirror authentic scientific practice, where students integrate synthesis, measurement, and interpretation to construct mechanistic and structural understanding. Project-based laboratory models have been proposed as a means to promote design thinking, data literacy, and critical reasoning beyond procedural execution.^1^ In such environments, students engage with experimental systems that evolve across multiple stages of investigation, allowing them to revisit prior knowledge while applying increasingly sophisticated analytical approaches.

One pedagogical framework that supports this progression is the spiral curriculum, in which students revisit core concepts at multiple points with increasing depth and complexity.^2,3^ In chemistry laboratories, spiral designs allow previously learned experimental skills to be revisited through new analytical contexts, helping students perceive connections among synthesis, structure, and function. Campbell*et al.* demonstrated that revisiting analytical and synthetic techniques across progressively complex laboratory activities promotes autonomy and strengthens students’ perception of laboratory work as an integrated scientific process.^4^ Similarly, project-based laboratory sequences centered on a single molecular system have been shown to enhance conceptual continuity and reinforce transferable analytical skills.^5,6^ In addition, emerging digital tools are beginning to reshape how students engage with chemical information. Artificial intelligence (AI) platforms capable of predicting physicochemical properties offer new opportunities for prelaboratory exploration and hypothesis generation. When used critically, such tools can support student reasoning by allowing predictions to be compared directly with experimental observations, fostering reflection on the reliability and limitations of computational assistance in chemical research.^7,8^

To address these instructional opportunities, we developed a spiral laboratory framework that links Organic Chemistry II and Instrumental Analysis through a shared molecular system. In this approach, students revisit the same chemical system across both courses with progressively greater analytical depth, allowing prior concepts to be reexamined and strengthened through increasingly sophisticated experimental methods. In Organic Chemistry, students synthesize ferrocenyl chalcones via base-catalyzed Claisen–Schmidt condensation while considering reaction efficiency and experimental design. These compounds later reappear as coded unknowns in the Instrumental Analysis Laboratory (IAL), where students construct identity profiles using multiple characterization techniques, including High Performance Liquid Chromatography (HPLC), UV–vis spectroscopy (UV-vis), Fluorescence spectroscopy (FLU), Fourier Transform Infrared Spectroscopy (FTIR), Nuclear Magnetic Resonance (NMR), Differential Scanning Calorimetry (DSC), and Thermogravimetric Analysis (TGA). Within this framework, students move from molecule synthesis to multi-instrumental characterization, mirroring the workflow used in chemical research and materials development.

The choice of ferrocene derivatives as the scaffold is deliberate. Ferrocene, [Fe­(η^5^-C_5_H_5_)]_2_ or bis­(η^5^-cyclopentadienyl) iron, has a crystal structure^9,10^ that consists of two cyclopentadienyl anions coordinated to a central Fe^2+^ ion (Figure 1). Ferrocene is thermally stable, undergoes a reversible one-electron redox process, and is highly reactive towards electrophiles, making derivatization feasible.^11^ Ferrocene’s impact as an organometallic has been thoroughly documented,^12^ including its applications in materials science^13–24^ (Figure 1). The feasibility to characterize Ferrocene’s properties makes it a suitable model for student projects.

**Figure 1:**
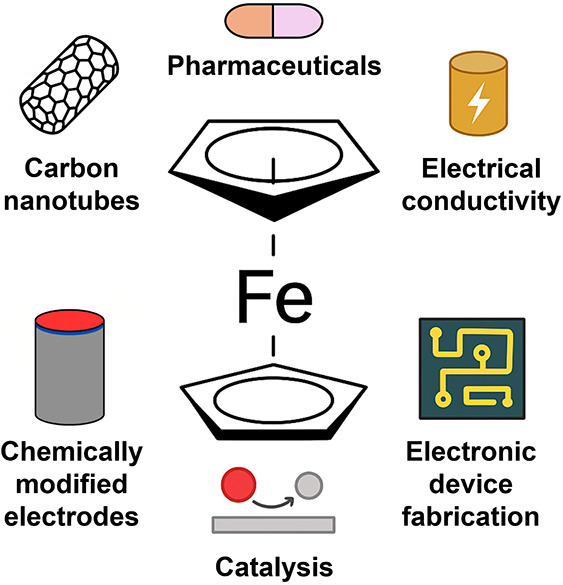
Molecular structure of ferrocene (figure generated with assistance from ChatGPT image-generation tool (OpenAI)).

A well-studied derivative in this field is ferrocenyl chalcones (FCs). These highly conjugated α,β-unsaturated systems feature an enone moiety bonded to a ferrocene unit on one end and an aromatic benzene ring on the other end. They can be classified as System 1 or System 3 depending on enone connectivity with respect to the carbonyl moiety (Figure 2). Their significance in this field is reflected in multiple applications (photovoltaics,^25,26^ fluorescence,^27,28^ and optics^29,30^ ). This laboratory experience uses FCs to immerse students in real-world challenges while reinforcing a spiral constructivist approach. Few studies report their use in academic laboratories, underscoring the relevance of this work.^31^ Our group performs research on ferrocenyl derivatives and applications;^32^ based on this, laboratory experiences were designed, developed, and implemented as part of the OCL curriculum over a decade.^33^

**Figure 2:**
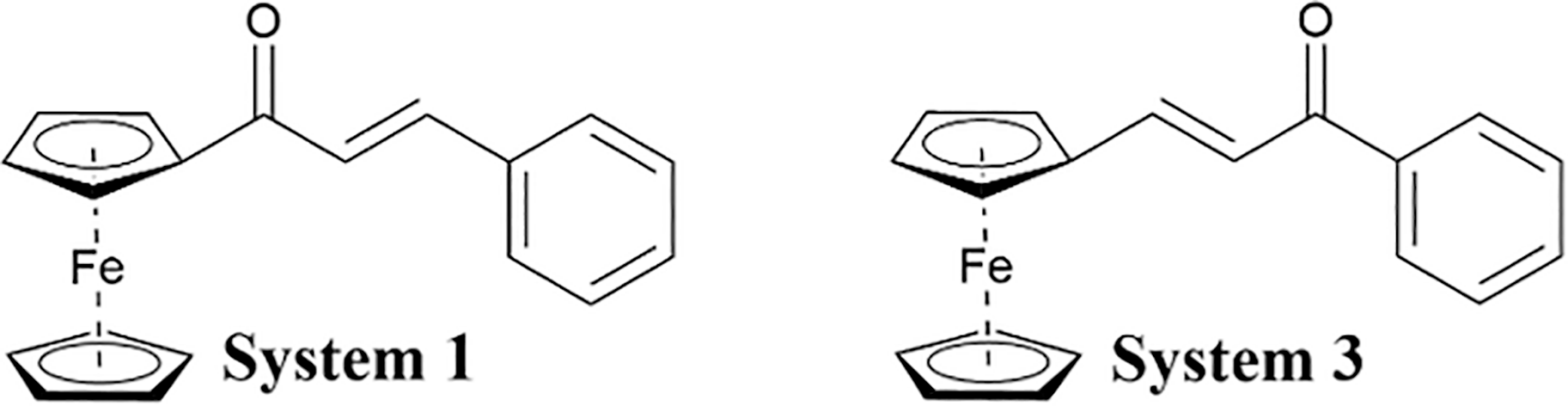
System 1 and System 3 ferrocenyl chalcones.

In the proposed experience, students also employ AI-assisted prediction tools to anticipate spectroscopic signatures and chromatographic behavior of potential FC derivatives. By comparing predicted results with experimental data, students engage in reflective analysis of both chemical evidence and digital prediction tools, strengthening evidence-based reasoning in the interpretation of analytical results. After revisiting the same molecular system across sequential courses, this spiral laboratory design allows students to experience the progression chemists follow when designing, synthesizing, and validating new molecules. The approach transforms isolated laboratory exercises into a coherent investigative process that integrates organic synthesis, analytical instrumentation, and materials characterization. This article describes the design, implementation, and educational outcomes of this cross-course laboratory model, which aims to strengthen interdisciplinary reasoning and promote a deeper understanding of structure–property relationships in undergraduate chemistry education.

## Pedagogical Framework and Student Learning Outcomes

This laboratory experience integrates contemporary educational theory with evidence-based practices through a spiral curriculum (Figure 3). FCs serve as the unifying context: first synthesized in organic chemistry, then analyzed with diverse instrumental methods to integrate data sets and identify an unknown. The sequence from fundamental methods (TLC, melting point, basic spectroscopy) to multi-instrumental characterization, culminates in cross-method data interpretation. The design emphasized authentic problem-solving, collaboration, and iterative hypothesis refinement.

**Figure 3:**
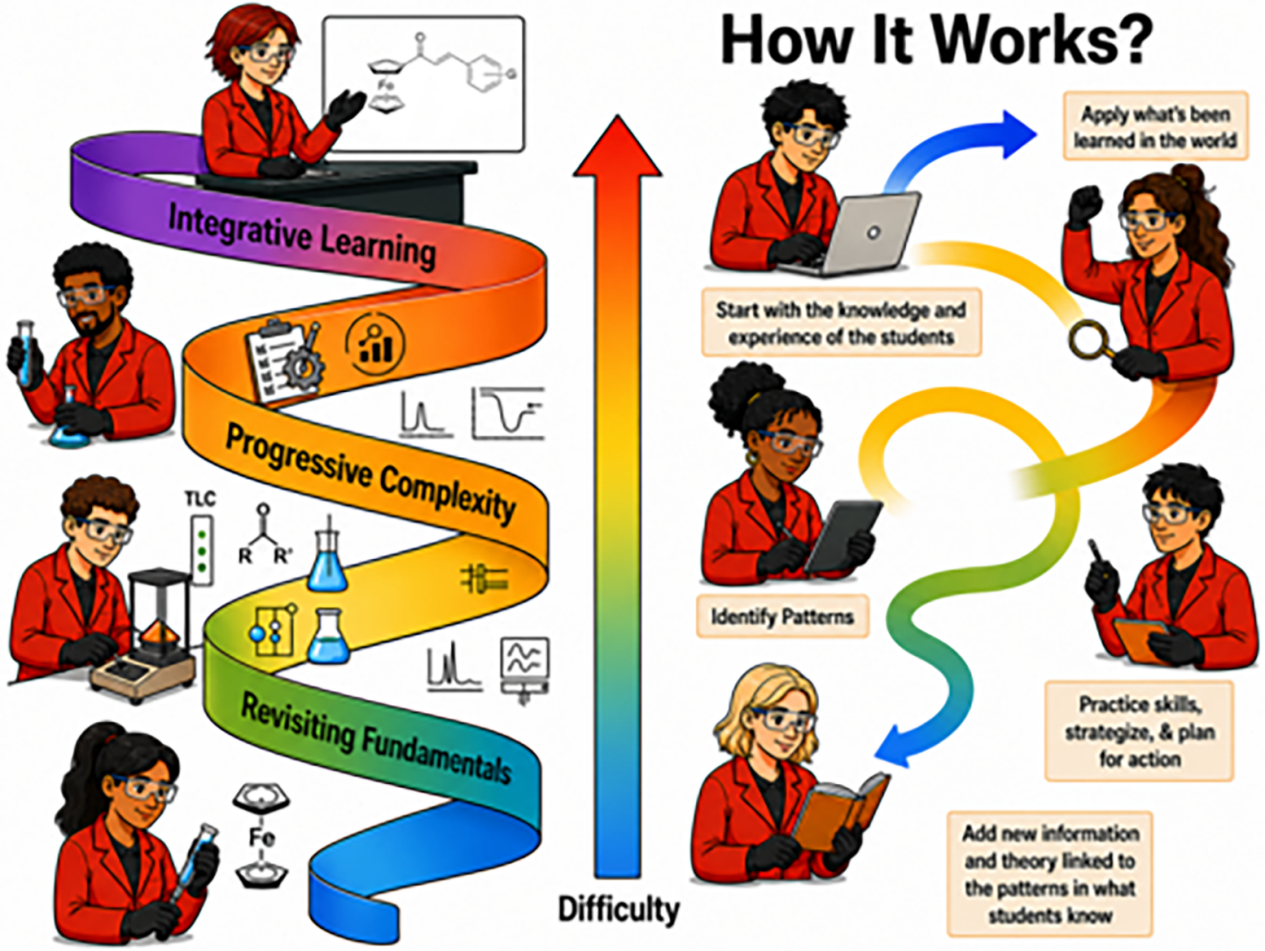
Spiral laboratory framework for the ferrocenyl chalcones module (figure generated with assistance from ChatGPT image-generation tool (OpenAI)).

The students used AI tools such as ChatGPT or similar chatbots to simulate spectra and analytical predictions, then compared these with their own results. In this way, AI functioned as a metacognitive partner, enhancing critical reasoning, digital literacy, and confidence, outcomes consistent with current evidence.^1,2,4,7^ Figure 4 presents the alignment between the learning objectives, laboratory activities and their assessments.

**Figure 4:**
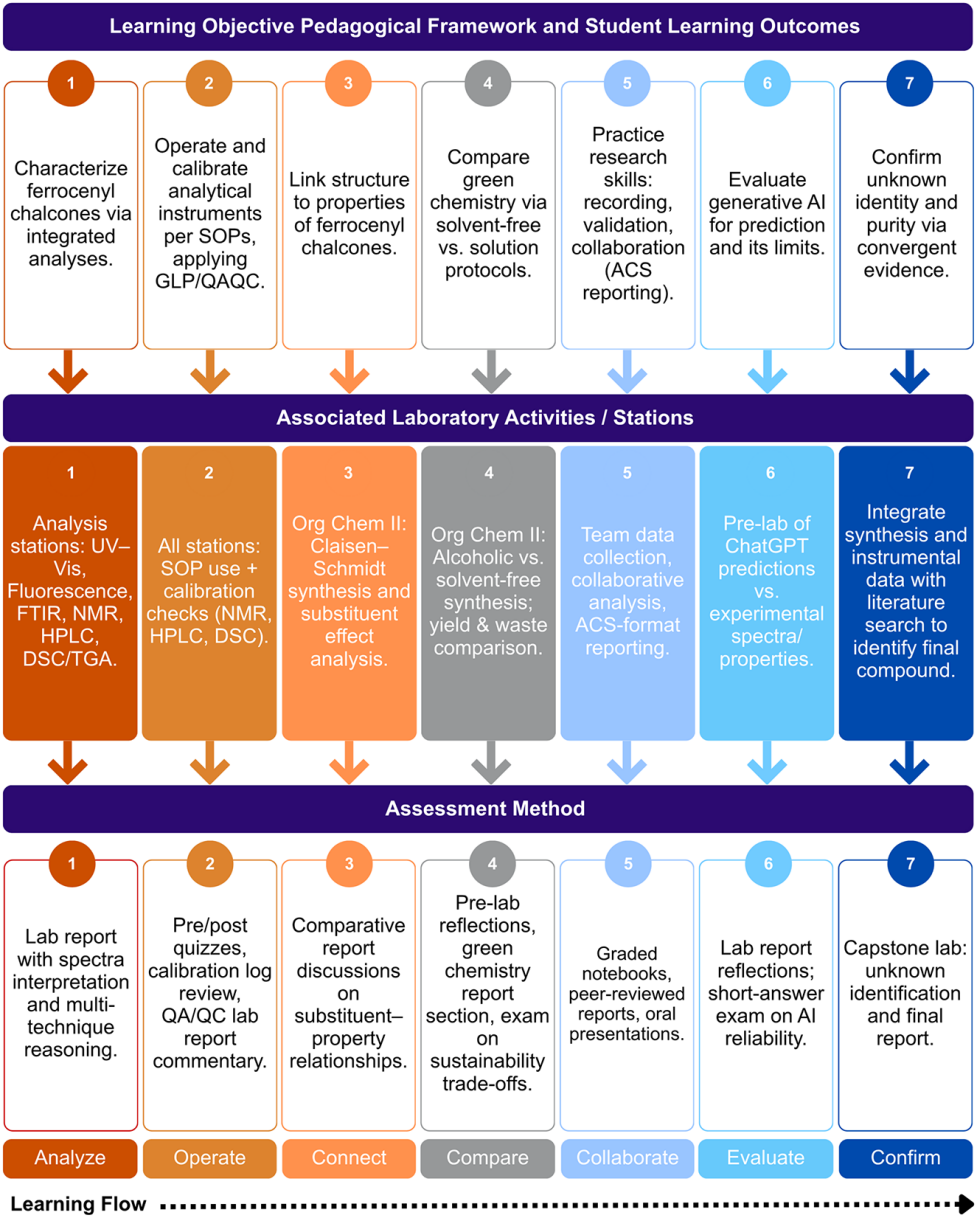
Alignment of learning objectives with laboratory activities and assessments in the ferrocenyl chalcones experiment.

## Implementation

The laboratory module was implemented in the IAL course within the undergraduate Chemistry BS curriculum at a large Hispanic-serving university. Both Organic Chemistry II and Instrumental Analysis are four-credit upper-division courses. The IAL is taken by third- and fourth-year chemistry majors who have completed prerequisite coursework in organic and analytical chemistry, ensuring foundational experience in molecular synthesis and chemical analysis. The present work incorporates multi-instrumental characterization of these compounds in the IAL. In this course, the same compounds reappear as coded unknown samples, which students must identify and characterize using multiple complementary analytical techniques.

To manage the cognitive demands associated with multi-instrumental analysis, the activity was structured with intentional scaffolding. Initial laboratory sessions focused on instrument calibration and data acquisition, allowing students to develop familiarity with each technique before engaging in integrative interpretation. The final session emphasized data synthesis, where students compared results across techniques to construct an identity profile for their unknown. The prelaboratory lecture and AI-assisted prediction activity provided conceptual support to anticipate expected analytical signatures before entering the laboratory. Together, these elements distributed the workload across multiple sessions and supported students in managing analytical complexity.

The project was implemented across four sections of the IAL over two semesters, with 10–14 students enrolled per section, and a total of 48 participants working in groups of 2–3 students. The instructional module spanned four class meetings: a preparatory lecture followed by three laboratory sessions, each lasting 3 h.

The module began with a 90 min class period, introducing spectroscopic techniques, the analytical methods used in the activity, and the workflow for characterizing unknown compounds. The session also assessed students’ prior knowledge, included discussion of strategies for identifying FCs derivatives using complementary instrumental methods and introduced AI-assisted prediction tools. Following this preparatory session, students completed three rotating laboratory sessions organized as station-based activities to collect data for their assigned unknown sample. Each session included multiple analytical stations covering complementary characterization techniques: HPLC, UV–vis, FLU, FTIR, ^1^H NMR, DSC, and TGA.

The first two laboratory sessions focused on students preparing calibration curves, verifying instrument performance using reference standards, and acquiring spectroscopic, chromatographic, and thermal data for their unknown. The final session emphasized data interpretation and integration across analytical techniques, where students compared results from multiple instruments to construct an evidence-based identity profile for their assigned FC derivative. Following the laboratory sessions, students had a two-week period to prepare their report, after which they completed a postexperience quiz and surveys.

Coordination between courses occurred through sequential planning by the respective instructors. The Organic Chemistry instructor implemented the synthesis experiment in the laboratory curriculum, while the Instrumental Analysis instructor designed and implemented the characterization module. The instructors collaborated to ensure conceptual continuity, guiding students in interpreting data and integrating results.

Teaching assistants (TAs) participated as instructional partners throughout the laboratory sessions: training students in the safe operation of analytical instruments, supporting calibration procedures and data acquisition, and providing feedback during data analysis and interpretation.

Student deliverables included two written products designed to promote integrative reasoning. First, students prepared a technical data sheet summarizing the results from each technique. Second, students completed an ACS-style laboratory report, justifying the identity and purity of their assigned compound using evidence from multiple analytical methods. These reports required students to interpret spectroscopic, chromatographic, and thermal data collectively, reinforcing the use of techniques to reach evidence-based conclusions.

Participation in the research component was voluntary and did not affect course grades. Students were informed about the purpose of the study and provided consent for their anonymized data to be used for research purposes. All data were deidentified prior to analysis to ensure participant confidentiality.

## Prelaboratory Activity

A 90 min structured prelaboratory exercise engaged students in predicting the physicochemical characterization of FCs before experimentation. By analyzing potential impurities, anticipating spectroscopic and chromatographic behaviors, and generating AI-assisted predictions for instrumental techniques, students developed hypothesis-driven reasoning and quality assurance strategies. AI was integrated as a structured prediction and critical-evaluation tool rather than a source of definitive answers. Students first generated independent predictions using traditional spectroscopy resources and chemical reasoning before comparing them with AI-generated outputs. Students used generative AI platforms, including ChatGPT (OpenAI, GPT-4) and Google Gemini. The pedagogical goal was to encourage students to critically evaluate algorithmic predictions, identify limitations, and justify conclusions using experimental evidence. Figure 5 summarizes the seven interconnected prelaboratory components, including their descriptions, learning objectives, and alignment with chemistry competencies. SLF_SupportingInformation_1_PrelaboratoryExercise includes the prelaboratory worksheet, with representative prompts, student reflection questions, and guidelines used to standardize the AI-assisted prediction activity across laboratory sections.

**Figure 5:**
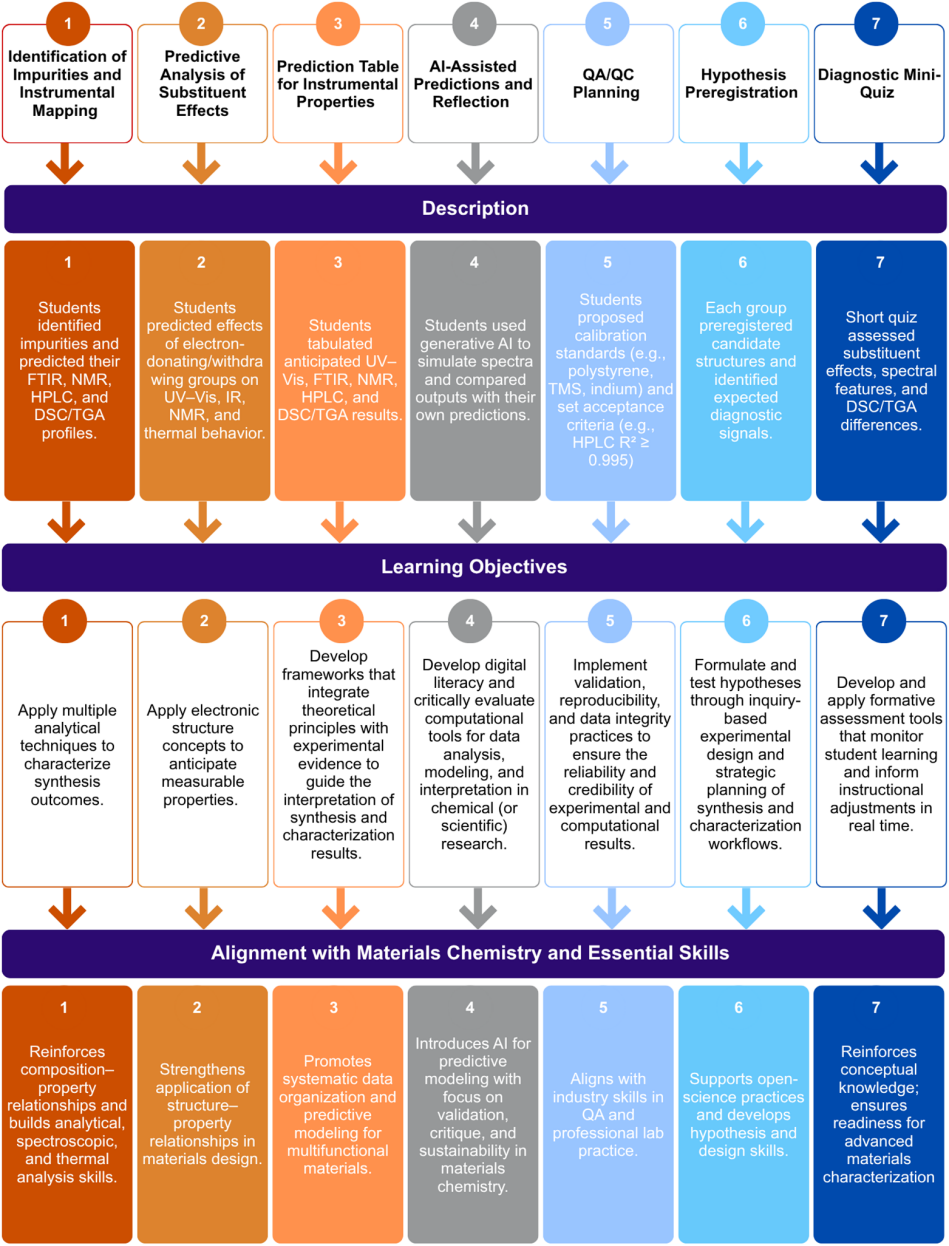
Components of the prelaboratory activity and alignment with materials and instrumental analysis chemistry learning and skills.

## Experimental Overview

Students received a Laboratory Manual detailing experimental procedures, calibration, and sample analysis (SLF_SupportingInformation_2_LaboratoryManual). An Instructor’s Manual was also prepared, providing information on instrumentation, sample preparation, operating parameters, expected results, troubleshooting, analysis of the unknown, pedagogical emphasis, and example technical sheet (SLF_SupportingInformation_3_ ExperimentalSetupAndCalibrationProcedures).

### Synthesis Stage

Chalcones are commonly synthesized by the Claisen-Schmidt condensation due to minimal workup and fewer side products.^34^ In the OCL course, this is a two-period experience. During the first period, students react acetylferrocene (nucleophilic enolate) with the corresponding benzaldehyde (electrophile) to produce substituted ferrocenyl chalcones (Figure 6). Students work in pairs; each is assigned a different substituent.

**Figure 6:**
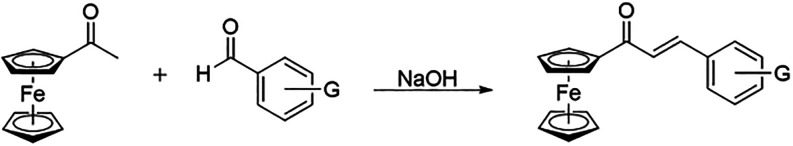
Synthesis of System 1 ferrocenyl chalcones [G = H, 2-OCH_3_, 4-OCH_3_, 3,4-OCH_3_, 4-NO_2_, 3-Cl, 4-Cl, 4-Br].

Students compared the reaction under alcoholic and solvent-free conditions to highlight Green Chemistry principles. In the second period, products are purified and characterized using TLC, FTIR, and melting points. Trends in reactivity based on substituents, yields, reaction time, and other parameters are discussed to introduce research-like analysis. Additional details can be found in the student manual for the OCL course.^33^ After students complete this experience, the samples are stored by the instructors in sealed vials and handed to the instrumental analysis instructors.

### Characterization Stage

Each group received a sample containing either pure ferrocenyl chalcone (Figure 7) or a mixture with unreacted starting material to simulate incomplete reactions and impurity scenarios. Although these chalcones differ only by their aromatic substituent, each has distinct properties (e.g., melting point, polarity, solubility). The experiment aimed to confirm molecular identity and purity while guiding students to integrate evidence across instrumental techniques and apply professional practices. Conducted in a rotating, station-based format, small groups operated different instruments and later exchanged results, emphasizing technical proficiency and interpretative reasoning. Students performed quality assurance and calibration checks before analyzing their unknown by all techniques to assess electronic transitions, functional groups, substitution patterns, thermal behavior, and sample purity. Rather than treating each data set independently, students synthesized results across methods, mirroring authentic research practices and reinforcing collaboration, cross-checking, and interpretation.

**Figure 7:**
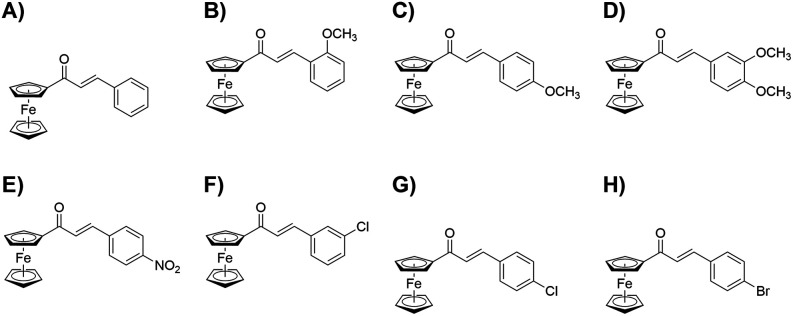
Ferrocenyl chalcones synthesized via Claisen–Schmidt condensation: (A) 3-(phenyl)-1-ferrocenyl-2-propen-1-one (unsubstituted phenyl), (B) 3-(2-methoxyphenyl)-1-ferrocenyl-2-propen-1-one (*o*-OCH_3_), (C) 3-(4-methoxyphenyl)-1-ferrocenyl-2-propen-1-one (*p*-OCH_3_), (D) 3-(3,4-dimethoxyphenyl)-1-ferrocenyl-2-propen-1-one (3,4-di-OCH_3_), (E) 3-(4-nitrophenyl)-1-ferrocenyl-2-propen-1-one (*p*-NO_2_), (F) 3-(3-chlorophenyl)-1-ferrocenyl-2-propen-1-one (*m*-Cl), (G) 3-(4-chlorophenyl)-1-ferrocenyl-2-propen-1-one (*p*-Cl), and (H) 3-(4-bromophenyl)-1-ferrocenyl-2-propen-1-one (*p*-Br).

## Hazards and Safety

All procedures followed ACS and institutional safety guidelines. Students wore appropriate PPE, secured hair, footwear, and handled FCs carefully. Flammable solvents were used in fume hoods, with waste segregated. Instrumental analyses were faculty-supervised. No OSHA-defined highly hazardous substances were employed.

## Assessment of the Experience

Student learning was evaluated through performance rubrics, a prelaboratory exercise, laboratory reports, quizzes, and reflective questionnaires assessing conceptual understanding and experimental proficiency. To evaluate analytical reasoning and scientific communication skills, laboratory reports were assessed using a rubric aligned with course learning objectives (SLF_SupportingInformation_7_EvaluationRubrics). The rubric evaluated students’ ability to document procedures, interpret spectroscopic and chromatographic data, integrate evidence from multiple analytical techniques, and construct evidence-based conclusions. Pre- and postlaboratory assessments measured gains in data analysis and problem-solving, while a postexperience questionnaire captured students’ perceptions of learning. All evaluation instruments are included in the Supporting Information (SLF_SupportingInformation_5_PrelaboratoryEvaluation; SLF_SupportingInformation_6_ComprehensiveImpactEvaluation; SLF_SupportingInformation_7_EvaluationRubrics).

## Results and Discussion

The prelaboratory prepared students for the spiral experiment, reinforcing spectroscopic interpretation, analytical reasoning, and professional practice. Working in groups reduced cognitive load and promoted peer instruction. Reference tables modeled after the organic sequence helped students transfer FTIR, ^1^H NMR, and UV–vis knowledge to new contexts, supporting the construction of impurity maps and prediction tables linking unreacted acetylferrocene, isomers, and decomposition products across FTIR, ^1^H NMR, HPLC, and DSC/TGA. For example, as shown in Figure 8, most students accurately identified bathochromic and hypsochromic shifts, although fewer connected them to HOMO–LUMO gaps or resonance effects. Overall, the prelaboratory strengthened conceptual integration, data literacy, and professional scientific habits.

**Figure 8:**
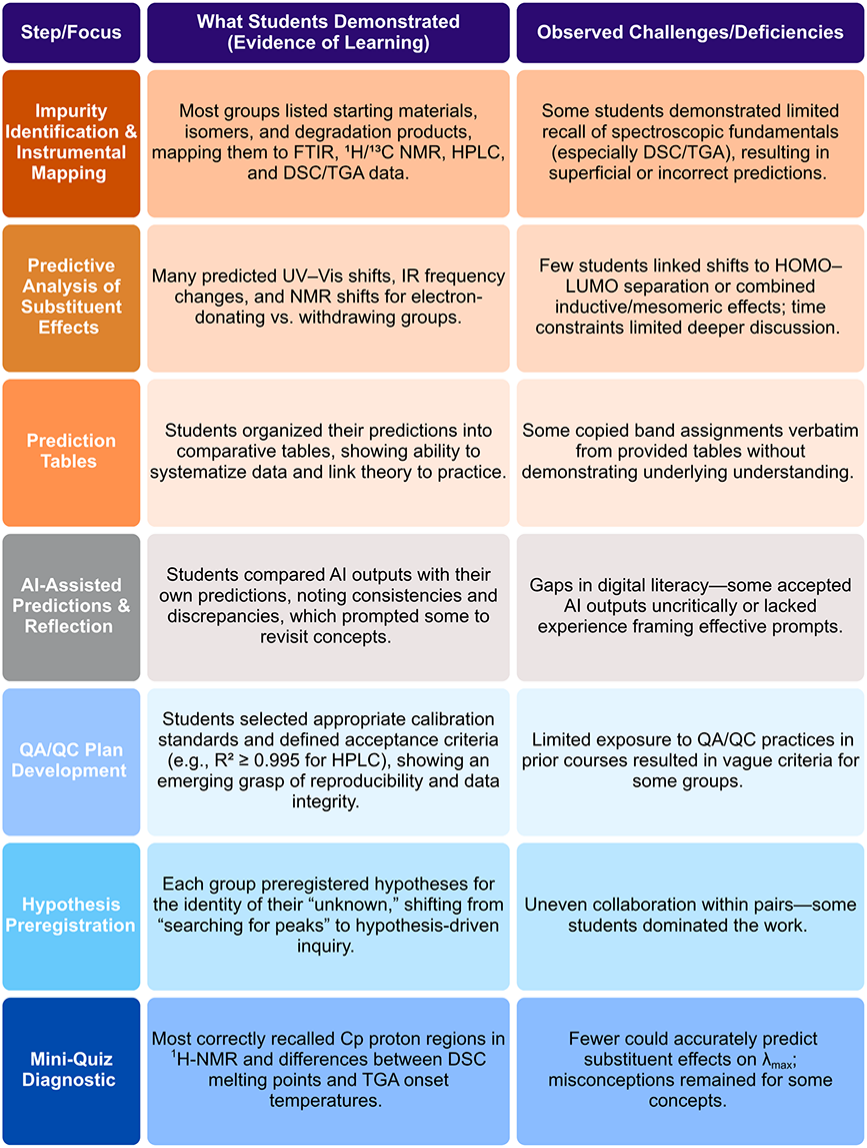
Evidence of learning and challenges across prelaboratory components in the ferrocenyl chalcones experiment.

AI was integrated through a two-stage sequence: an AI-assisted prediction activity in the prelaboratory phase and a reflective evaluation quiz after the experiment. Students identified potential impurities, predicted their appearance in chromatograms or spectra, and constructed tables estimating spectroscopic and chromatographic signatures for chalcones bearing different substituents. After completing these predictions, students used AI-assisted tools to generate comparable predictions. For example, students were instructed to use prompts such as “Generate qualitative predictions and reasonable values for UV-Vis (λ_max_), FTIR (key bands), ^1^H-NMR (peaks/features), HPLC (relative retention index and expected purity), and thermal analysis (DSC/TGA) of a ferrocenyl chalcone bearing a [X] substituent on the aromatic ring. Briefly explain the influence of the substituent (electron-donating/electron-withdrawing) in each technique.”

This comparison was designed to encourage critical evaluation rather than passive acceptance of AI outputs. AI responses often produced qualitatively reasonable predictions: identifying C=O stretching bands near 1650 cm^–1^ in FTIR or cyclopentadienyl proton signals near 4.3–4.8 ppm in ^1^H NMR. However, students also identified several limitations, including overly precise numerical predictions for UV–vis (λ_max_), FTIR bands, and insufficient consideration of experimental conditions such as solvent effects. Through this process, students revised their predictions and justified why only qualitative spectral trends could be expected prior to experimental measurement.

The postlaboratory quiz evaluated students’ ability to critically interpret AI-assisted predictions. Rather than generating predictions, students were presented with AI-generated analytical predictions for a substituted FC and were asked to evaluate them. They identified which predictions were chemically reasonable, detected predictions that were uncertain or questionable, and explained how a chemist could verify the predictions experimentally. Students also determined which analytical technique would provide the strongest evidence for confirming or refuting the prediction and explaining potential errors in spectral assignments.

Mean rubric scores increased from 2.1–2.8 in the preactivity to 3.0–3.3 in the postactivity (Table 1). All improvements were statistically significant (*p* < 0.05) with moderate to large effect sizes (Cohen’s d = 0.76–1.45). The largest improvement occurred in critical evaluation of AI predictions, indicating substantial gains in students’ ability to identify algorithmic limitations and propose experimental validation strategies.

**Table 1:** Learning Gains in Analytical Reasoning and AI-Critical Evaluation from Pre- and Postlaboratory Activities

Task	Pre-Activity Student Action	Post-Activity Student Action	Pre Mean ± SD	Post Mean ± SD	Mean Gain	***p*** value	Effect Size (d)
Identification of Possible Impurities	Listed potential impurities in ferrocenyl chalcone synthesis (unreacted reagents, byproducts, decomposition products) and provided chemical justification.	Reassessed impurity predictions after comparing AI predictions and experimental reasoning.	2.7 ± 0.6	3.2 ± 0.5	+0.5	0.008	0.82
Prediction of Instrumental Manifestation (HPLC, FTIR, NMR, DSC/TGA)	Predicted how impurities or products would appear in chromatograms or spectra (retention times, peaks, spectral signals).	Refined predictions after evaluating AI outputs and considering which techniques would confirm predictions experimentally.	2.6 ± 0.7	3.1 ± 0.6	+0.5	0.012	0.76
Substituent Effects on UV–vis λ_max_	Predicted qualitative spectral shifts based on substituent electronic effects and conjugation.	Revisited predictions and explained spectral behavior using molecular orbital reasoning and comparison with AI predictions.	2.8 ± 0.5	3.3 ± 0.5	+0.5	0.005	0.90
Integrated Instrumental Prediction	Completed a prediction table for estimating values for UV–Vis, FTIR, NMR, HPLC purity, and thermal behavior for substituted chalcones.	Compare predictions with AI outputs and identify discrepancies or unrealistic values.	2.4 ± 0.7	3.0 ± 0.6	+0.6	0.004	0.95
Critical Evaluation of AI Predictions	Limited AI interaction; students relied primarily on prior knowledge to generate predictions.	Analyzed AI responses, identified possible AI hallucinations or uncertainties, and proposed corrections or experimental verification.	2.1 ± 0.8	3.2 ± 0.6	+1.1	<0.001	1.45
Experimental Validation Strategy	Suggested analytical techniques to verify predictions but often without prioritizing one method.	Identified the most decisive analytical technique to resolve discrepancies between AI predictions and experimental data.	2.5 ± 0.6	3.1 ± 0.5	+0.6	0.007	0.87

Improvements were also observed in students’ ability to connect chemical structures with analytical observations. For example, values for predicting instrumental manifestations of impurities and reasoning about substituent effects on UV–vis spectra increased. In addition, students demonstrated a stronger ability to design experimental validation strategies. These improvements indicate that students increasingly interpreted analytical predictions using chemical theory and prioritized techniques such as NMR or HPLC based on their diagnostic power.

Qualitative analysis of student responses further supports these findings. Initial predictions were frequently based on general spectral patterns or reference tables, whereas postactivity explanations increasingly incorporated mechanistic reasoning such as conjugation effects, resonance stabilization, and molecular orbital considerations. Likewise, many students explicitly stated that AI-assisted predictions should be interpreted cautiously and validated experimentally. These observations indicate that the activity promoted a shift from passive use of computational tools toward critical scientific evaluation of algorithmic outputs. Taken together, the combined pre- and postlaboratory activities demonstrate that integrating AI into the learning cycle can serve as a metacognitive scaffold for analytical reasoning.

## Experimental Results

Students employed various characterization techniques to determine the identity and purity of their unknown FC. Example results from students that worked with 3-(4-bromophenyl)-1-ferrocenyl-2-propen-1-one (Figure 9) are shown below along with the expected student data report (Figure 10 ^35–41^ ). Common student experimental issues can be found in SLF_SupportingInformation_3_ Experimental Setup And Calibration Procedures. Overall, students who received an “impure” sample had more difficulty analyzing data and recognizing the possibility of having starting materials. As students obtained new data from each technique, they went through the process of reformulating their initial hypothesis and testing it. Moreover, the experimental design enabled students to gain new knowledge, fill theory-to-practice gaps, and overcome the challenge of concluding the identity of an unknown ferrocenyl chalcone.

**Figure 9:**
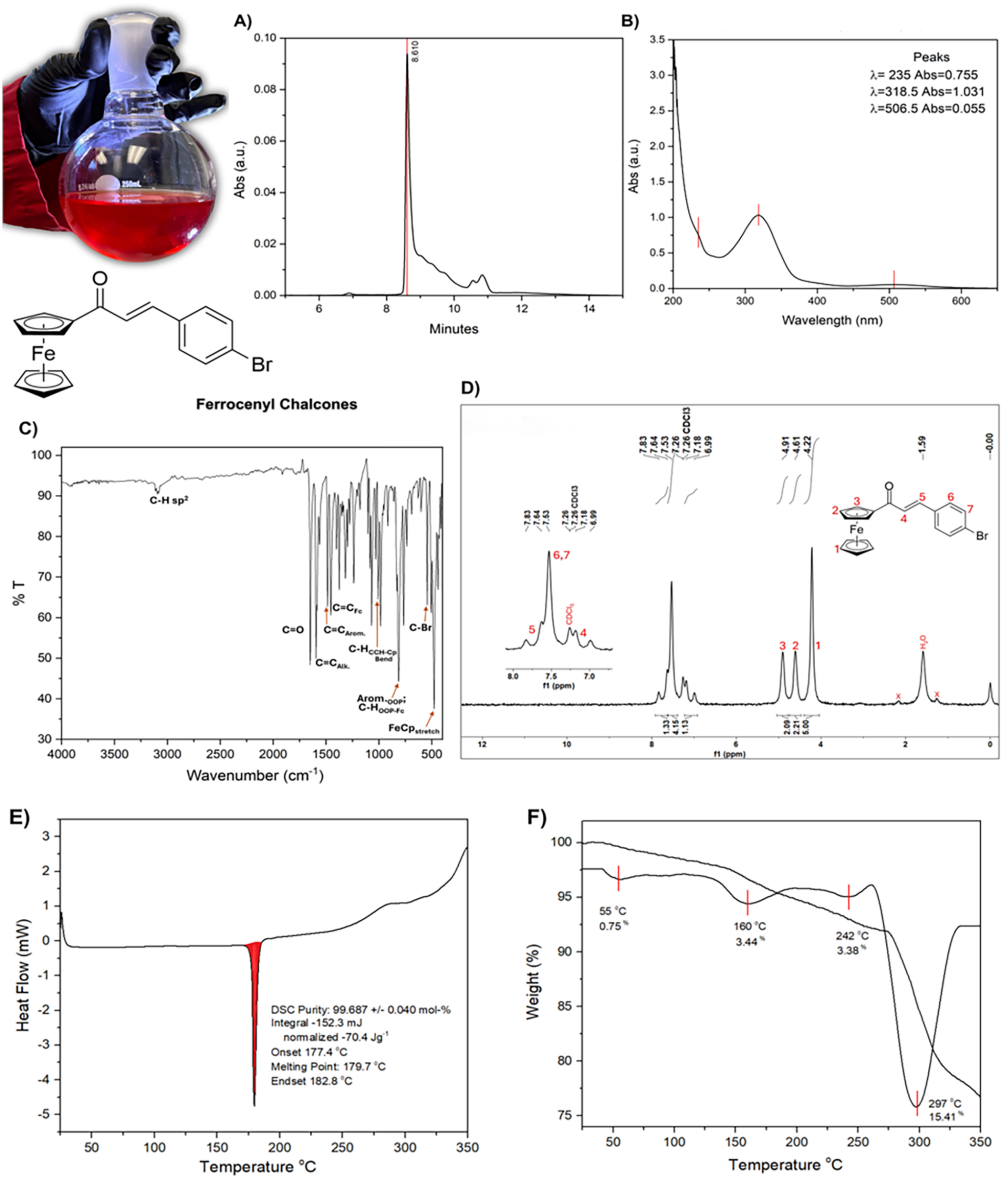
Representative examples of expected experimental results for ferrocenyl chalcone. The results in this figure correspond to ferrocenyl chalcone 3-(4-bromophenyl)-1-ferrocenyl-2-propen-1-one. Data appear in the following order: (A) HPLC, (B) UV–vis, (C) FT-IR, (D) ^1^H NMR, (E) DSC, and (F) TGA. Instrument information and experimental details/parameters can be found in the Supporting Information (SLF_SupportingInformation_3_ ExperimentalSetupAndCalibrationProcedures).

**Figure 10:**
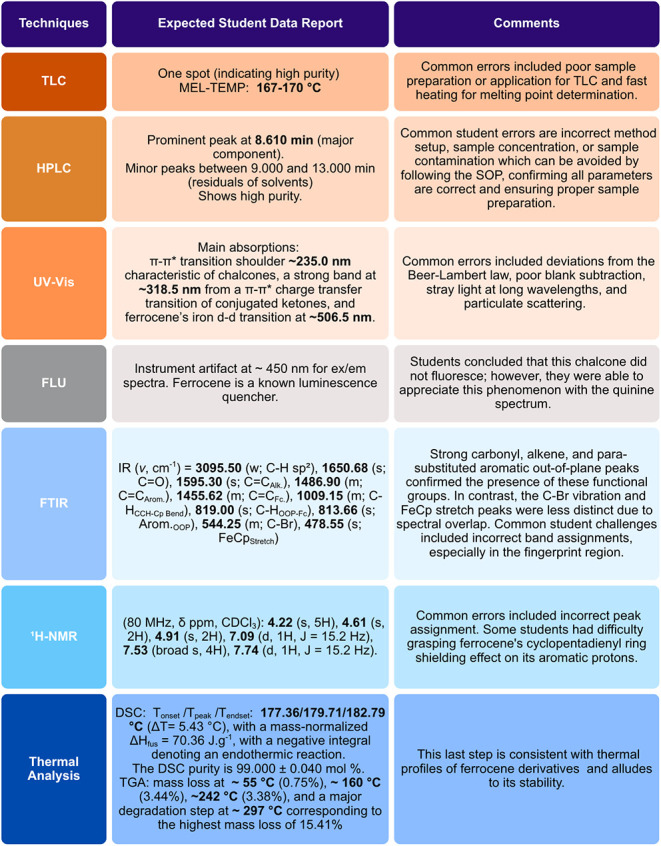
Example of expected experimental results for ferrocenyl chalcone 3-(4-bromophenyl)-1-ferrocenyl-2-propen-1-one for the following analytical techniques: TLC, HPLC, UV–Vis, FLU, FTIR, ^1^H-NMR, and thermal analysis (DSC/TGA).

## Learning Outcomes and Student Perceptions

### Learning Outcomes

The FCs experiment provided an inquiry-based learning experience that required students to synthesize chemical reasoning, instrumental skills, and data interpretation across multiple analytical methods. A structured sequence of questions for all characterization techniques served as cognitive scaffolds guiding progression from observation to analysis. From the HPLC section, students demonstrated competency in using retention time to assess compound polarity and purity. Most groups correctly identified a single dominant peak as a high-purity product and multiple peaks as evidence of unreacted acetylferrocene or byproducts. UV–vis analysis strengthened understanding of electronic transitions and molecular structure: students accurately noted λ_max_ values, attributing these to π–π* and charge-transfer transitions in the FC system. Bathochromic shifts were attributed to electron-donating groups and extended conjugation, demonstrating insight into structure–property relationships. In the fluorescence component, students correlated scan velocity and sensitivity settings to signal resolution, and used Jablonski diagrams to distinguish absorption, excitation, and emission.

FTIR responses showed that most students identified diagnostic bands for C=O, C=C_alkene_, Fe–C, aromatic ring out-of-plane, and halogen stretches. They correctly linked these to functional groups in a *para*-halogenated ferrocenyl chalcone and noted extra bands as possible moisture or impurity. NMR results demonstrated strong spectral reasoning: students identified Cp signals, aromatic doublets, and trans α,β-olefinic protons confirming the *E*-configuration. Some groups also recognized doublets indicating para-substitution and minor peaks from unreacted starting material.

DSC/TGA analyses deepened students’ understanding of thermal events and decomposition. They accurately associate single sharp endothermic peaks with melting and purity, while multiple or broadened peaks indicated impurities or phase transitions. TGA analyses revealed multiple degradation onsets and major mass losses, linked to decomposition of substituents and ferrocene rings. Many students recognized that minimal early mass loss suggested little solvent retention, demonstrating improved evaluation of data quality.

Across the reports, students integrated results from multiple techniques to confirm functional groups (FTIR, NMR), assess purity (HPLC, DSC), and evaluate stability (TGA). Many recognized that agreement across independent analytical methods increased confidence in compound identification. UV–vis spectroscopy and HPLC were frequently used as complementary qualitative and quantitative validation tools. Students also demonstrated professional competencies in quality assurance and quality control (QA/QC), referencing calibration logs, replication of measurements, and documentation of experimental deviations. Table 2 summarizes the rubric-based evaluation of the reports, including proficiency levels across major analytical competencies. Overall, students developed strong skills in scientific communication, data visualization, and interpretation of spectroscopic and chromatographic data, while challenges remained in areas such as multitechnique integration and QA/QC reasoning. The experiment therefore achieved its pedagogical goal, moving students beyond isolated technique-based analysis toward a systems-level understanding of chemical characterization consistent with the objectives of a spiral, interdisciplinary laboratory curriculum.

**Table 2:** Evaluation of Student Laboratory Reports, Common Analytical Reasoning Errors, and Corrective Feedback in a Spiral Instrumental Analysis Activity

Learning Dimension	Assessment Metric	Mean Score (4-pt rubric)	Groups Achieving Proficiency (≥3) Out of 14 groups
Scientific Writing and Organization	Title, abstract clarity, ACS formatting, logical structure	3.6 ± 0.4	12 (86%)
Conceptual Understanding of Analytical Techniques	Explanation of theory behind TLC, HPLC, FTIR, NMR, UV–vis, DSC, TGA	3.3 ± 0.7	10 (71%)
Experimental Documentation	Completeness and reproducibility of experimental procedures	3.4 ± 0.5	12 (86%)
Data Visualization	Quality and clarity of figures, chromatograms, spectra, and thermograms	3.4 ± 0.4	12 (86%)
Multi-Technique Data Interpretation	Interpretation of spectral and chromatographic features	3.4 ± 0.5	11 (79%)
Structural Confirmation and Purity Assessment	Integration of chromatographic, spectroscopic, and thermal evidence	3.1 ± 0.7	9 (64%)
QA/QC and Analytical Reliability	Discussion of calibration, standards, and GLP practices	2.9 ± 0.8	7 (50%)
Evidence-Based Conclusions	Ability to synthesize analytical evidence to justify compound identification	2.8 ± 1.1	7 (50%)

A postlaboratory quiz was administered to assess the interpretation of spectroscopic/thermal data to determine compound identity and purity. Specifically, students analyzed ^1^H NMR splitting patterns, identified possible impurities, interpreted thermograms, and ranked analytical techniques according to their usefulness for compound characterization. The results indicated that students developed a strong understanding of the comparative analytical value of different instrumental methods. When ranking the usefulness of techniques for determining compound identity and purity, as shown in Figure 11, ^1^H NMR was identified as the most important and informative method, followed by thermal analysis, whereas UV–vis spectroscopy received the lowest diagnostic ranking.

**Figure 11:**
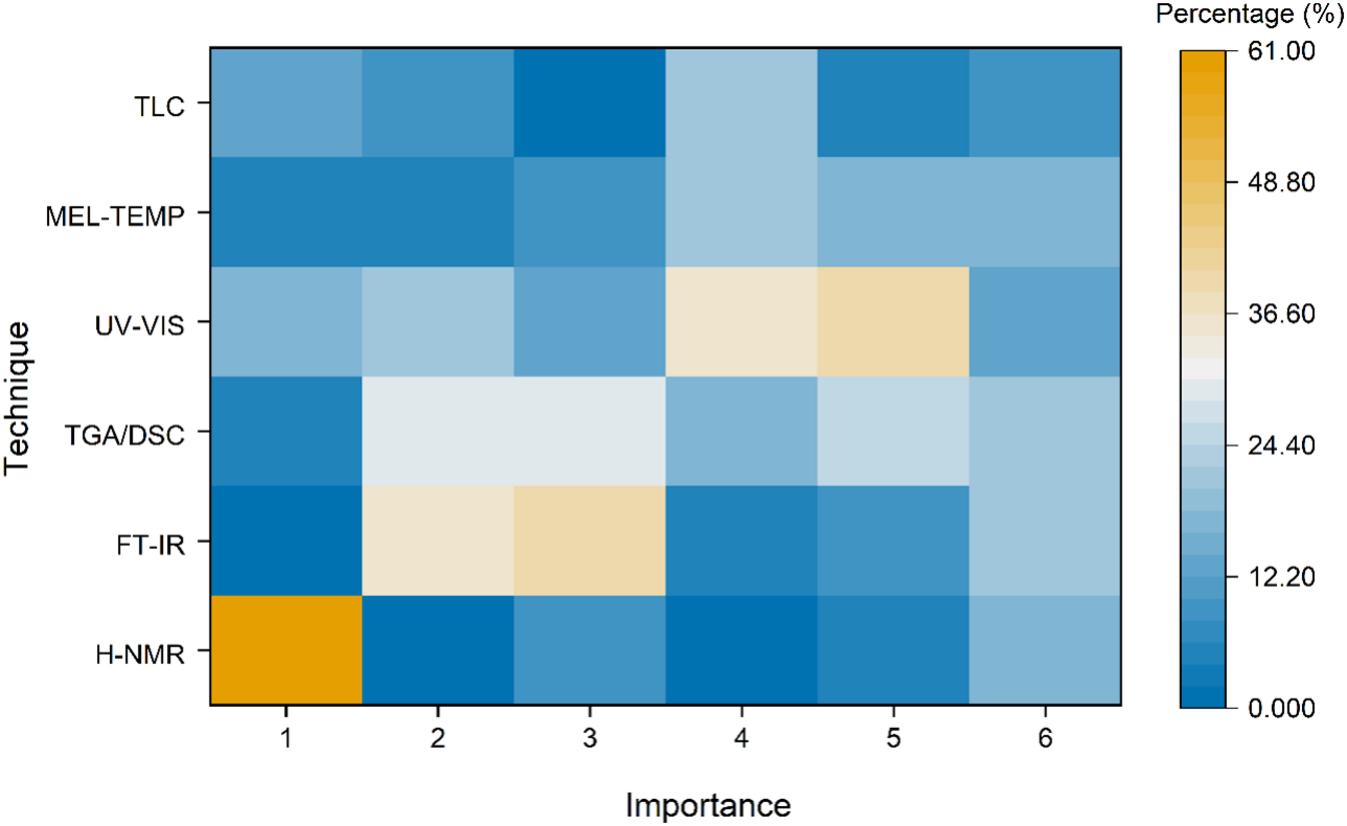
Heatmap percentage ranking of instrumental analytical techniques. Values based on student perspective of usefulness to determine compound identity and purity (1 = most important, 6 = least important).

Overall, the postassessment results indicated that a majority of students were able to interpret instrumental data and integrate evidence from multiple analytical methods to evaluate compound identity and purity. The combination of relatively high success rates in spectral interpretation, thermal analysis, and analytical method evaluation suggests that the laboratory activity supported the development of instrumental literacy, multitechnique reasoning, and evidence-based decision-making.

### Student Perception and Feedback

A student questionnaire (SLF_SupportingInformation_6_Comprehensive Impact Evaluation) revealed strong perceived learning gains (Figure 12). Over 80% agreed that the laboratory enhanced their experimental, problem-solving, and transferable skills. AI integration indicated advanced digital literacy, while interest in materials science and QA/QC emphasis increased reflecting appreciation for calibration and data reliability (Figure 12A). Students also strongly agreed that the laboratory connected Organic Chemistry and Materials Science and helped them apply theory to practice. Participants reported improved data analysis and experimental design skills, integrating multiple analytical methods to achieve a holistic understanding of chemical properties (Figure 12B).

**Figure 12:**
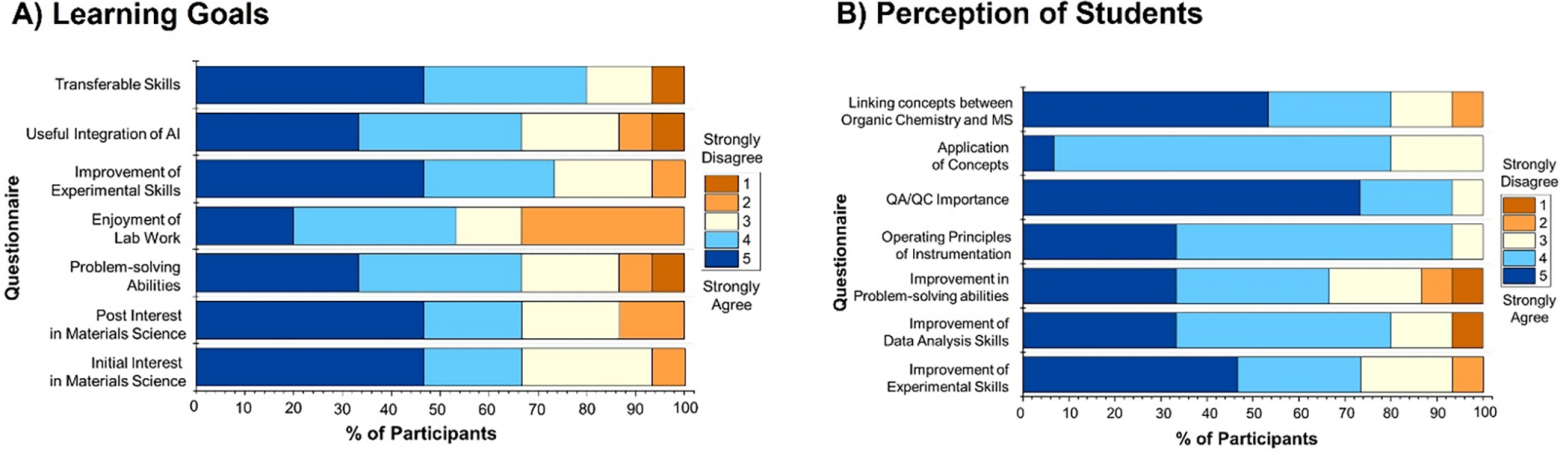
Student learning outcomes and perceptions from the spiral laboratory experience: (A) learning goals and (B) student perceptions summarize participants’ self-reported gains following the ferrocenyl chalcone spiral laboratory activity integrating synthesis and multi-instrumental analysis (HPLC, UV–vis, FLU, FTIR, NMR, DSC, and TGA).

Focus group feedback described the experience as “very good” and “helpful” for integrating synthesis with instrumentation, boosting confidence, and engaging students through inquiry-based “detective-like” problem solving. However, some found the use of multiple analyses cognitively demanding and suggested additional scaffolding. Despite technical challenges with instruments such as HPLC and NMR, students valued the spiral model’s inquiry-driven design, which enhanced engagement, professional competence, and interdisciplinary thinking. Overall, the laboratory effectively strengthened instrumental skills, critical reasoning, and enthusiasm for materials-focused research, aligning with modern chemical education goals. Moving forward, reinforcing FTIR and NMR training in the organic chemistry curriculum is recommended to provide more hands-on experience with sample preparation, experimentation, and data analysis, thereby improving problem-solving skills before the instrumental analysis course.

## Conclusion

The spiral FCs laboratory demonstrates how integrating synthesis, characterization, and AI can enhance materials education. Students develop critical thinking, design competencies, and interdisciplinary connections, as well as being exposed to important concepts such as Green Chemistry and sustainability, and materials science. Moreover, students are exposed to real world challenges of identifying an unknown compound and using analytical techniques to overcome this challenge. In addition, working collaboratively with peers helped students build working skills and collaborative learning. There are some areas for improvement, such as workload distribution and developing better AI prompts for predictions, yet the goal of spiral learning was met, and student feedback supported the success in this process.

## Supplementary Material



## Notes

Prelaboratory activity that students carry out before the experimental sessions take place, with document also including guiding questions, tables for predicting compound properties, AI prompts, and a diagnostic assessment, document sent to students and includes background information, experimental procedures for calibration and sample analysis, guiding questions for analysis, and technical sheets, Instructor’s Manual detailing experimental details of the different techniques (instruments used, calibration/preparation, critical parameters, expected results, common issues and troubleshooting, analysis of the unknown, and pedagogical emphasis) and example technical sheet, list of guiding and follow-up questions for students to analyze the collected data and make a final conclusion on the identity of their unknown, document used to assess students’ ability to predict, justify, and plan analytical characterization of ferrocenyl chalcones across multiple techniques while integrating AI-based reasoning, QA/QC, and scientific reflection, questionnaire sent to students after the activity to gather feedback on the experiment, focused on the impact on student’s attitudes toward materials chemistry, views on laboratory work, and understanding of new concepts, and all the rubrics used for evaluating student work, including prelaboratory experience, results integration sheets, and laboratory report (PDF)
