# Supplementary material for: Spiral Integration of Organic Synthesis and Instrumental Characterization Using Ferrocenyl Chalcones
Source: J Chem Educ. 2026 Jul 21;103(8):4475–87. doi: 10.1021/acs.jchemed.5c01473 (PMC13472136; doi:10.1021/acs.jchemed.5c01473)
Supplement: Supplementary file 1 [file ed5c01473_si_001.pdf]

Spiral Integration of Organic Synthesis and Instrumental Characterization Using Ferrocenyl Chalcones

Supporting Information

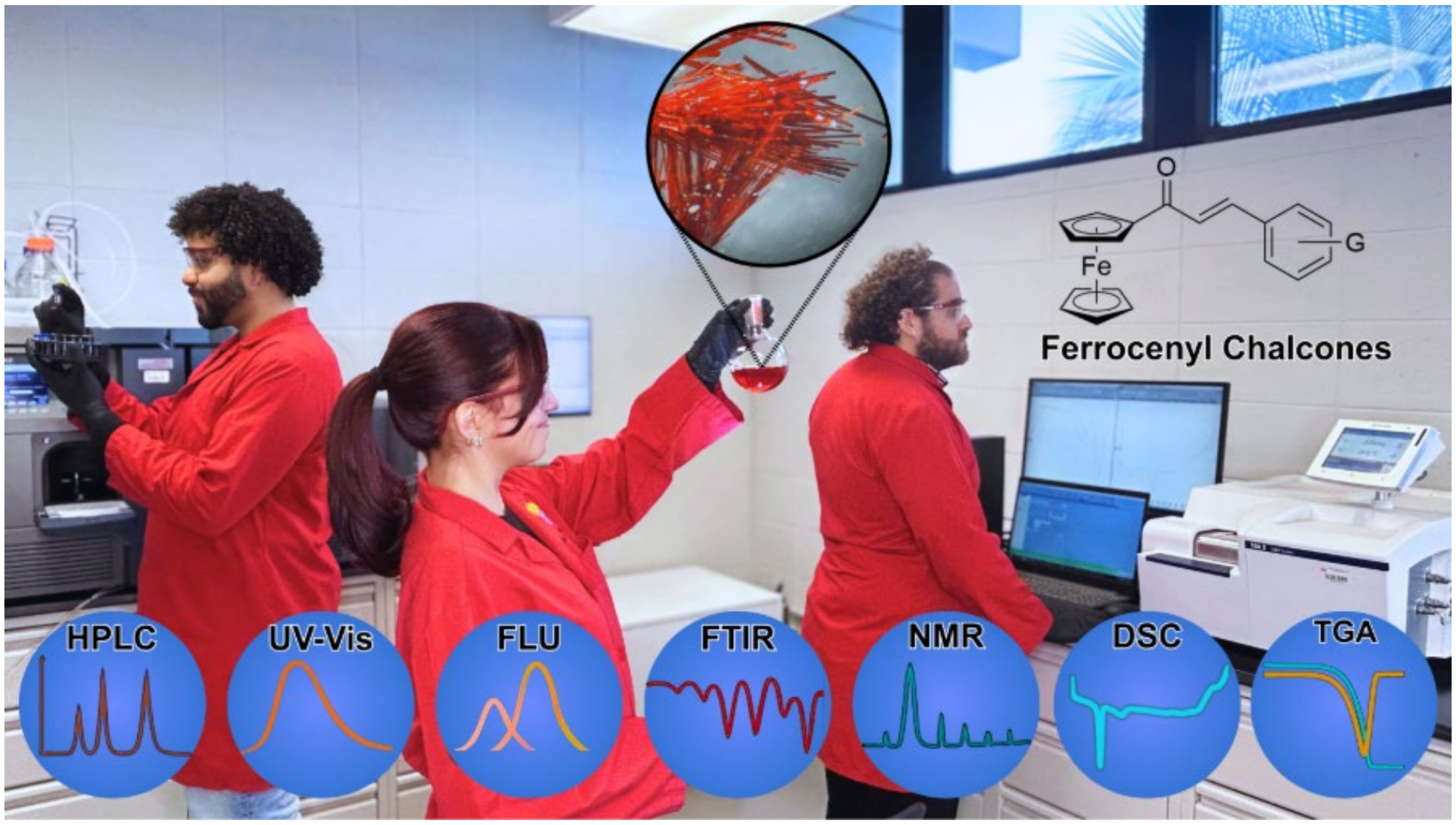

Liz M. Díaz-Vázquez, Alejandro Burgos-Suazo, Ángela Cruz-Lugo, Jeremy Rivera-Sánchez, Raisa M. De Jesús Torres, Vanessa Álvarez-Carrillo, Ángel L. Morales-Cruz, Ingrid Montes-González*

Authors Affiliation:

University of Puerto Rico, Río Piedras Campus, San Juan, 00925-2537, Puerto Rico

Contact Information for Corresponding Author:
Liz M. Díaz-Vázquez
Department of Chemistry
Email: liz.diaz2@upr.edu

Supporting Information (SI) Map

Linking SI to Manuscript Sections

The table below shows where each ***SI*** file is used in the manuscript and what it contains

Manuscript Section	Supporting Information file Title (*SI*)
**Pre-Laboratory Section**	**SLF_SupportingInformation_1_PrelaboratoryExercise**<br>Pre-laboratory worksheet, guiding questions, prediction tables, and AI prompts<br>**SLF_SupportingInformation_5_PrelaboratoryEvaluation**<br>Pre-laboratory assessment instrument
**Experimental Overview - Laboratory Implementation**	**SLF_SupportingInformation_2_LaboratoryManual**<br>Student lbaoratory manual (procedures, calibration, sample analysis, technical sheets<br>**SLF_SupportingInformation_3_ExperimentalSetup AndCalibrationProcedures**<br>Instructor manual with detailed experimental setup, calibration protocols, expected results and troubleshooting
**Data Analysis & Interpretation**	**SLF_SupportingInformation_4_StudentManual Questionnaire**<br>Guiding and follow-up questions for data interpretation and compound identification
**Assessment of the Experience**	**SLF_SupportingInformation_5_PrelaboratoryEvaluation**<br>Assessment of prediction and planning skills<br>**SLF_SupportingInformation_7_EvaluationRubrics**<br>Rubrics for pre-laboratory work, data integration, and lab report
**Student Perception & Feedback**	**SLF_SupportingInformation_6_ComprehensiveImpact Evaluation**<br>Post-activity student questionnaire on learning gains and perceptions
**Figures and Representative Data**	**SLF_SupportingInformation_3_ExperimentalSetup AndCalibrationProcedures**<br>Instrumental parameters, calibration details, and representative datasets referenced in figures

Table of Contents

1. SLF_SupportingInformation_1_PrelaboratoryExercise 1-5
Pre-laboratory activity that students carry out before the experimental sessions take place. The document also includes guiding questions, tables for predicting compound properties, AI prompts, and a diagnostic assessment.

2. SLF_SupportingInformation_2_LaboratoryManual 6-31
This document is sent to students and includes background information, experimental procedures for calibration and sample analysis, guiding questions for analysis, and technical sheets.

3. SLF_SupportingInformation_3_ExperimentalSetupAndCalibration Procedures 32-54
This is an Instructor's Manual detailing experimental details of the different techniques (instruments used, calibration/preparation, critical parameters, expected results, common issues & troubleshooting, analysis of the unknown, and pedagogical emphasis) and an example technical sheet.

4. SLF_SupportingInformation_4_StudentManualQuestionnaire 55-58
The document has a list of guiding and follow-up questions for students to analyze the collected data and make a conclusion on the identity of their unknown. Questions are related to calibration, experimental parameters, and experimental results analysis.

5. SLF_SupportingInformation_5_PrelaboratoryEvaluation 59-61
Document used to assess students' ability to predict, justify, and plan analytical characterization of ferrocenyl chalcones across multiple techniques while integrating AI-based reasoning, QA/QC, and scientific reflection.

6. SLF_SupportingInformation_6_ComprehensiveImpactEvaluation.... 62-68
Questionnaire sent to students after the activity to gather feedback on the experiment, focused on the impact on student's attitudes toward materials chemistry, views on laboratory work, and understanding of new concepts.

7. SLF_SupportingInformation_7_EvaluationRubrics 69-76
The document includes all the rubrics used for evaluating student work, including pre-laboratory experience, results integration sheets, and laboratory report.

SLF_SupportingInformation_1_PrelaboratoryExercise

Pre-laboratory Exercise: Prediction of Properties and Instrumental Behavior of Ferrocenyl Chalcones

Taking into consideration this reaction for the preparation of ferrocenyl chalcones, answer the following questions:

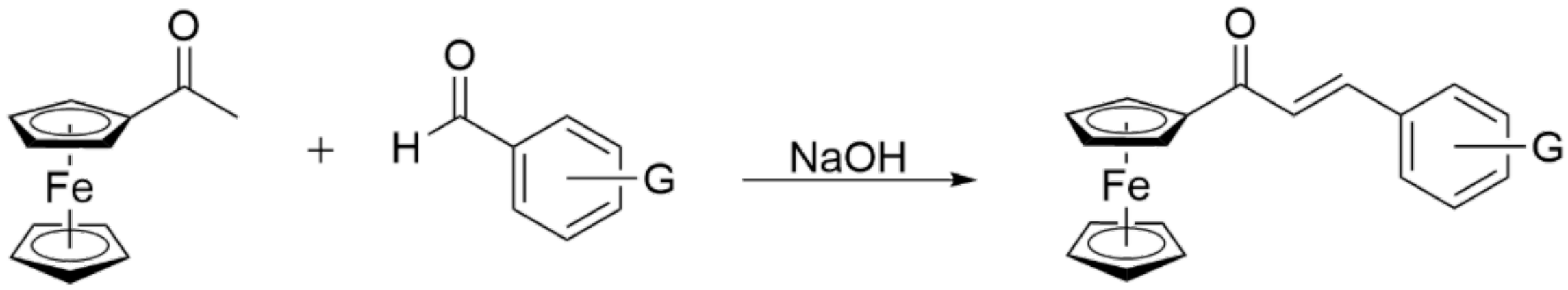

1. **Possible Impurities:**

List the possible impurities that could be present in your ferrocene chalcones sample, considering the following sources:

   a. Unreacted starting materials (e.g. 1-acetylferrocene, 4-bromobenzaldehyde).
   b. By-products of lateral condensations or other secondary reactions.
   c. Products of thermal or photochemical decomposition.
   d. Other (Specify).

2. **Instrumental Manifestation of Impurities:**

For each of the possible impurities identified in the previous point, describe how you would expect to detect and identify it on the following instruments:

   a. HPLC: What differences do you expect in the chromatogram in terms of retention times, relative area, etc.?
   b. FTIR: What missing or additional bands could you observe in the spectrum?
   c. ^{1}H-NMR/^{13}C-NMR: What unexpected signals would you expect to see in the spectrum?
   d. DSC/TGA: What variations would you expect in the melting or degradation curves?

3. **Predictive Analysis: Impact of the Substituent on the UV-Vis Spectrum**:

Consider the general structure of ferrocenyl chalcones: Ar-CH=CH–CO–ferrocene. How would you expect the nature of the aromatic substituent (Ar) to affect the wavelength of maximum absorption (λ_{max}) in the UV–Vis spectrum? Justify your answer in terms of molecular orbital theory and the electronic properties of the substituents (inductive and mesomeric effects).

4. **Prediction of Instrumental Properties:**

Complete the following table with your predictions about how the properties of potential ferrocenyl chalcones would manifest under instrumental analysis. Be specific about the expected values or trends you anticipate:

**Possible Chalcone (Specify Substituent):**	**Estimated λ_{max} UV/Vis (nm)**	**FTIR Peaks (cm^{-1})**	**Characteristic Peaks ^{1}H-NMR (ppm)**	**Estimated Purity on HPLC (Area %)**	**Expected Thermal Behavior (T_m, Degradation)**
Chalcone 1: **2-methoxy**					
Chalcone 2: **4-nitro**					
Chalcone 3: **3-chloro**					
Chalcone 4: **4-methoxy**					
Chalcone 5: **4-chloro**					
Chalcone 6: **4-bromo**					
Chalcone 7: **3,4-dimethoxy**					
Chalcone 8: **without substituent**					

5. **Predictions with Artificial Intelligence (AI)**

In this section you will generate AI-assisted predictions and compare them with your human predictions.

a. Base Prompts: using the AI chatbots of your preference (ChatGPT, Gemini, etc.) Copy/paste and adapt according to the selected substituent.

Prompt 1 (global):

"Generate qualitative predictions and reasonable values for UV-Vis (λ_{max}), FTIR (key bands), ^{1}H-NMR (signals/features), HPLC (relative retention index and expected purity), and thermal analysis (DSC/TGA) of a ferrocenyl chalcone bearing a [X] substituent on the aromatic ring. Briefly explain the influence of the substituent (electron-donating/electron-withdrawing) in each technique."

Prompt 2 (refinement):

"Refine the predictions assuming solvent [specify], HPLC method [column, mobile phase, gradient, flow rate], and DSC/TGA temperature program [standard program]. Propose ranges with tolerances."

Prompt 3 (comparison):
"Compare your predictions with this human-generated table [insert Section 4] and suggest critical discrepancies, possible sources of error, and which analytical technique would provide the greatest discriminating power."

a. AI Record (mandatory): Attach the prompts used and the key portions of the AI's responses. Indicate agreements and discrepancies with your Section 4 results.
b. Guided Reflection (short answers)
   i. In which analytical technique(s) was AI most useful, and why?
   ii. Where was it most uncertain or speculative?
   iii. What biases or hallucinations did you detect, and how would you mitigate them?
   iv. Which experimental datum would you prioritize to resolve discrepancies?

6. **QA/QC Plan**

Design a minimum quality assurance plan for your laboratory session:

a. Calibration/standards: Indicate which standards will be used, where, and for what purpose (e.g., wavelength standard for UV-Vis; polystyrene for FTIR; tetramethylsilane/solvent for NMR; retention-time standard for HPLC; indium for DSC).
b. Acceptance criteria: establish your acceptance criteria and actions if they are not achieved. For example: $R^2 \geq 0.995$ (HPLC); λ_{max} deviation ≤ 5–10 nm; FTIR within ±20–30 cm^{-1}; ^{1}H-NMR reference within ±0.02 ppm; blanks and duplicates where applicable.
c. Controls and verification: Instrumental blank, control sample (if available), and replicate analyses.
d. Brief checklist: Clean cell, appropriate solvent, acquisition parameters verified, proper sample storage.

7. **Pre-registration of Identity Hypothesis**

Before entering the laboratory, draft an identity hypothesis for your "unknown" sample:

a. Top-2 most probable candidates and justification.
b. Decisive technique expected to confirm or rule out the identity.
c. Critical signals/values to be sought (with tolerances).

8. **Mini Prelab Quiz (diagnostic assessment)**

Answer without using AI.

1. Indicate the effect of a p-OCH_3 group on λ_{max} and justify (MO, conjugation).

2. In FTIR, what pattern would you expect for p-NO_2? (asymmetric/symmetric NO_2 stretches).
3. Where would you expect Cp protons in ^{1}H-NMR, and why do they typically appear as relatively simple multiplets?
4. Define a purity criterion in HPLC (main peak and threshold).
5. What conceptual difference exists between T_m (DSC) and T_{onset} (TGA)?

SLF_SupportingInformation_2_LaboratoryManual

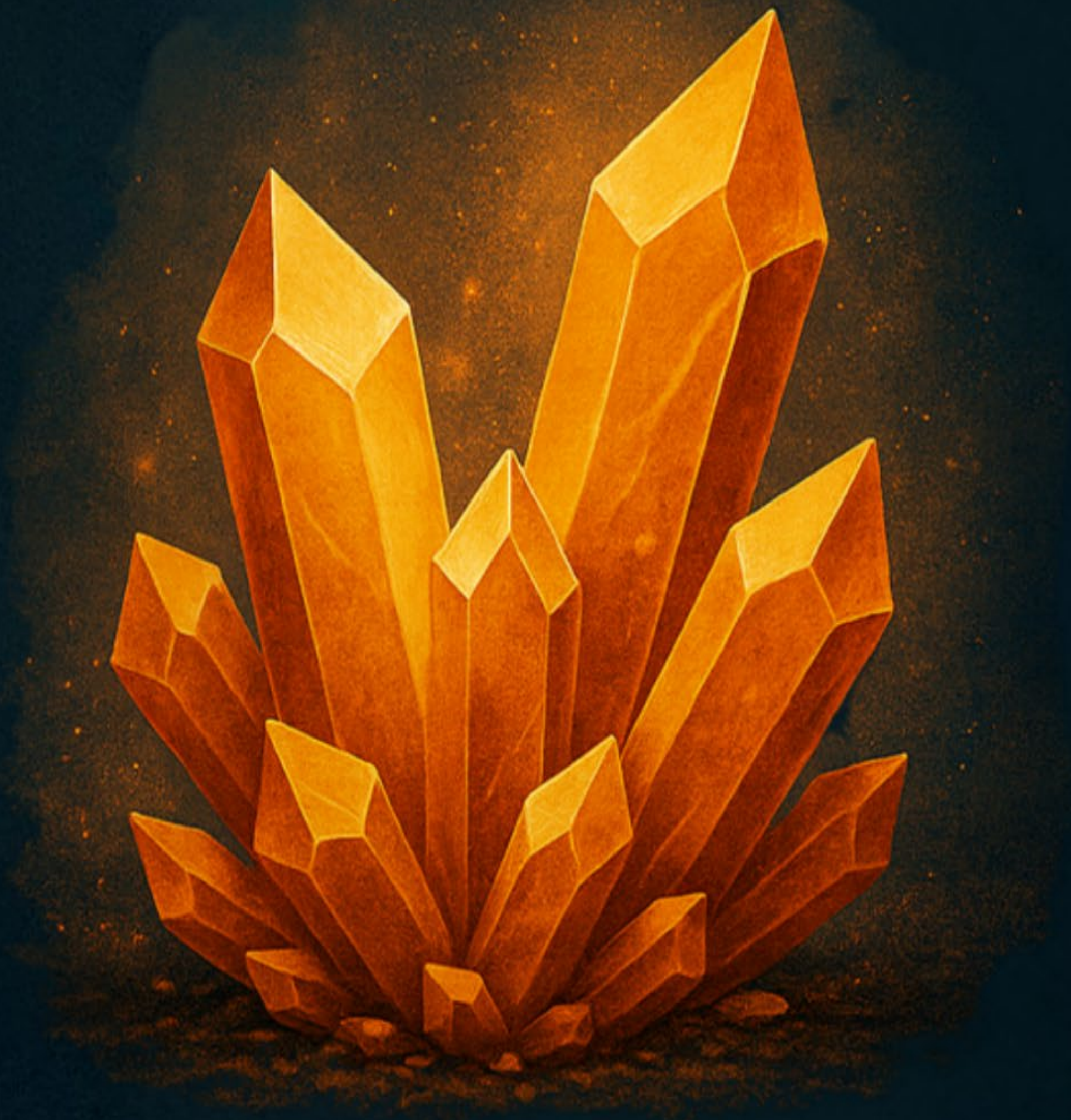

FERROCENYL CHALCONES ANALYSIS

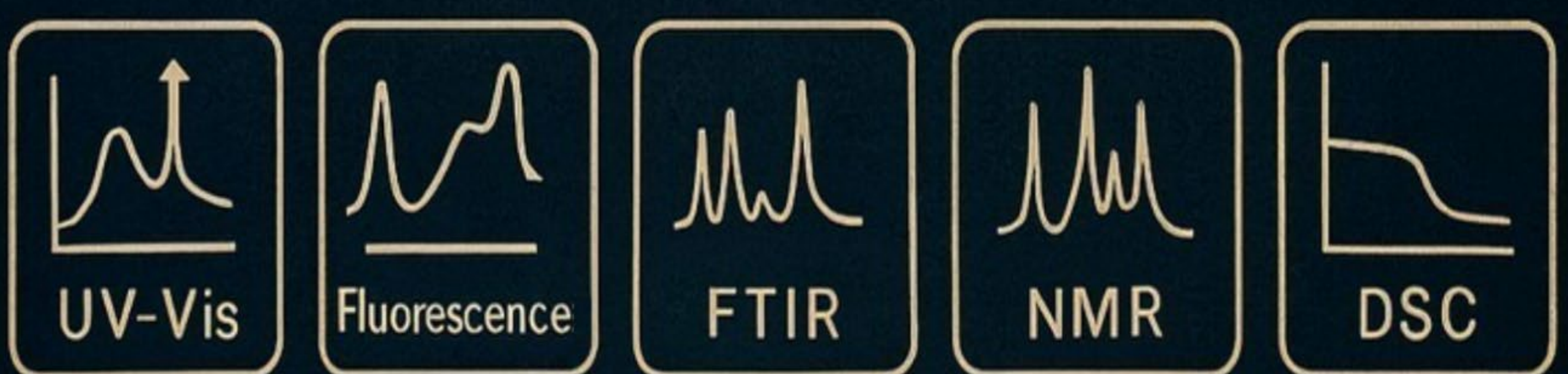

UNIVERSITY OF PUERTO RICO – RÍO PIEDRAS CAMPUS
CHEMISTRY DEPARTMENT
INSTRUMENTAL CHEMICAL ANALYSIS COURSE

LABORATORY MANUAL
Characterization and Quality Control of Ferrocenyl Chalcone Derivatives Utilizing Instrumental Techniques

Professors:

Dr. Liz M. Díaz-Vázquez
Dr. Ingrid Montes-González
Dr. Ángel Morales-Cruz

Teaching Assistants:
Alejandro Burgos-Suazo
Jeremy Rivera-Sánchez
Angela C. Cruz-Lugo
Raisa De Jésus-Torres
Vanessa Alvarez-Carrillo

Introduction

Ferrocene ($Fe(C_5H_5)_2$) is an organometallic compound whose stability, symmetry, and electronic properties make it an excellent scaffold for designing new materials and bioactive molecules. Its derivatives have been applied in materials science, catalysis, and pharmacology, with ferrocenyl chalcones reported as compounds with antitumor, antimicrobial, and antioxidant potential, as well as materials science applications. In your Organic Chemistry course, you synthesized ferrocenyl chalcones with different aromatic substituents through the Claisen–Schmidt condensation, a reaction that couples 1-acetylferrocene with different aromatic aldehydes (Ar-CHO) under basic catalysis. The general reaction can be represented as follows:

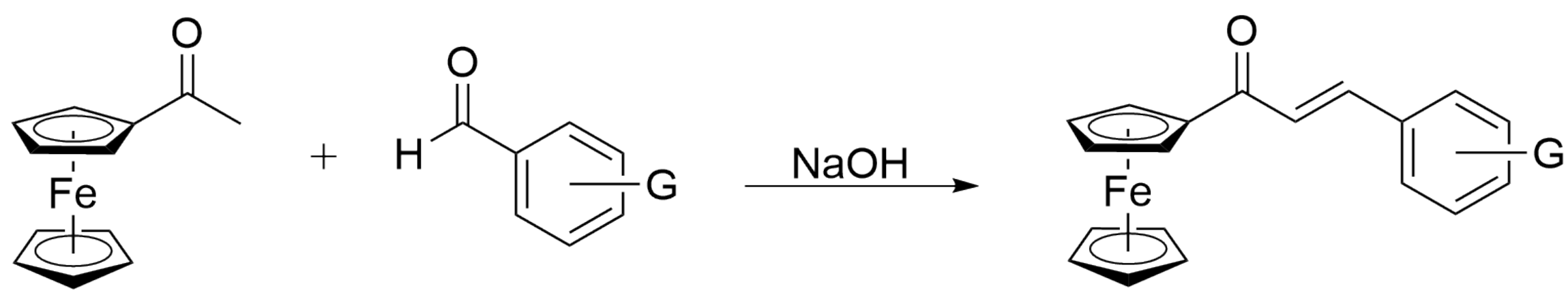

Figure 1: Claisen–Schmidt condensation

The transformation involves the addition of the methyl group α to the carbonyl of 1-acetylferrocene, generating an enolate that serves as the nucleophile to attack the carbon of the substituted benzaldehyde. After the formation of the β-hydroxyketone intermediate, followed by the elimination of a water molecule, the corresponding ferrocenyl chalcone is obtained, characterized by a carbon–carbon double bond conjugated with the carbonyl system and the aromatic ring. We know this α,β-unsaturated system as an enone. The aryl group (Ar) can carry different electronic substituents (electron-donating or electron-withdrawing), which modify the spectroscopic, electronic, and thermal properties of the synthesized ferrocenyl chalcone. For this exercise, consider the aromatic substituents (Ar) shown in Figure 2. These will influence the final behavior of the ferrocenyl chalcones:

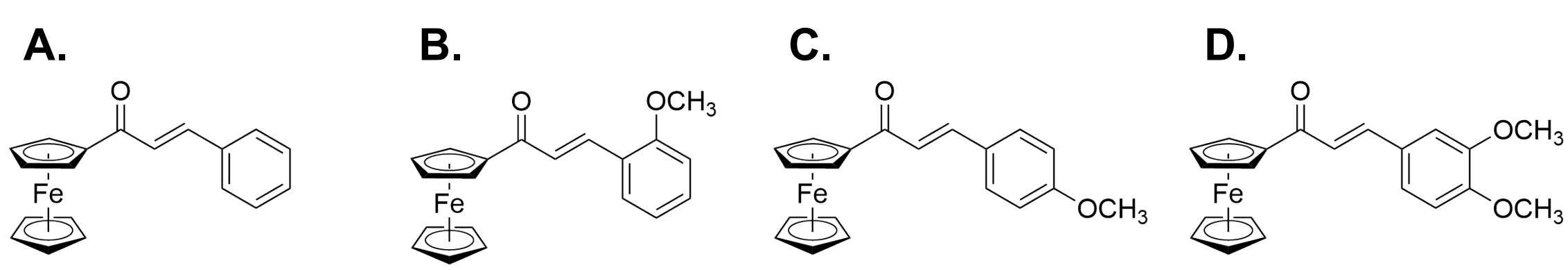

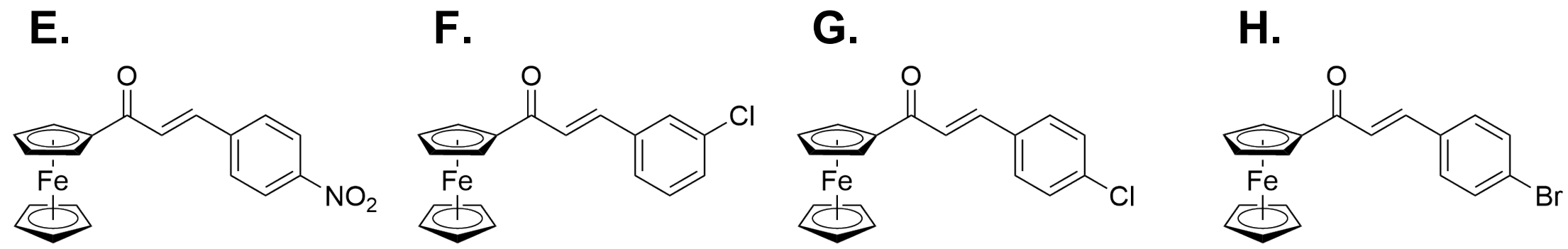

Figure 2. Ferrocenyl chalcones synthesized via the Claisen–Schmidt condensation of 1-acetylferrocene with substituted benzaldehydes:

A. 3-(Phenyl)-1-ferrocenyl-2-propen-1-one (unsubstituted phenyl),
B. 3-(2-Methoxyphenyl)-1-ferrocenyl-2-propen-1-one (p-OCH_3),
C. 3-(4-Methoxyphenyl)-1-ferrocenyl-2-propen-1-one (m-OCH_3),
D. 3-(3,4-Dimethoxyphenyl)-1-ferrocenyl-2-propen-1-one (3,4-di-OCH_3),
E. 3-(4-Nitrophenyl)-1-ferrocenyl-2-propen-1-one (p-NO_2),
F. 3-(3-Chlorophenyl)-1-ferrocenyl-2-propen-1-one (m-Cl),
G. 3-(4-Chlorophenyl)-1-ferrocenyl-2-propen-1-one (p-Cl),
H. 3-(4-Bromophenyl)-1-ferrocenyl-2-propen-1-one (p-Br),

In this first experience of the Instrumental Chemical Analysis course, you will face a common challenge in research: the characterization of an unknown sample. The material you will receive will be one of the ferrocenyl chalcones presented in Figure 2, organometallic compounds that are not only stable and versatile but have transformed the way we understand the relationship between metals and organic systems. Your task will be to confirm its identity and purity using, for the first time, a set of advanced instruments that constitute the foundation of modern analytical practice, including **UV-Vis, Fluorescence**, **FTIR**, **NMR**, **HPLC**, **DSC**, and **TGA**.

This experience is designed so that you go through and learn the complete process of characterization like a researcher would do it, or scientists in industry, technical fields or pharmaceuticals from the initial calibration that guarantees the validity of results to the final interpretation that connects structure, purity, and properties. By doing so, not only will you practice vanguard techniques but also you will interiorize the principles of Goof Laboratory Practices (GLP) and Quality Control/Assurance (QC/QA) that accompany all research in chemistry.

General Objectives

- Operate and calibrate advanced analytical instruments.
- Determine the physicochemical properties of synthesized chalcones.
- Interpret spectroscopic and chromatographic data.
- Apply GLP and QA/QC principles.
- Generate a professional technical report.

Principles of Safe Laboratory Practices

Before beginning experimental work, remember that laboratory safety is of utmost importance. At all times, you must follow safety rules and wear appropriate safety clothing, including a lab coat, goggles, and gloves. Please refer to https://institute.acs.org/acs-center/lab-safety/education-training/spanish-language-resources.html for more information about Safety in the Academic Chemistry Laboratory.

Procedure: The main objective of this initial practice is to familiarize students with a variety of essential instrumental analytical techniques for compound characterization. To achieve this objective, the practice is organized into seven workstations, each dedicated to a specific technique: **Station I**: (Liquid Chromatography – HPLC/UV-Vis), **Station II**: (UV-Vis Spectroscopy), **Station III**: (Fluorescence), **Station IV**: (FTIR Spectroscopy), **Station V**: Nuclear Magnetic Resonance (NMR), and **Station VI**: Differential Scanning Calorimetry – DSC and **Station VII**: Thermogravimetric Analysis – TGA). Each group of students will receive an unknown sample of a ferrocenyl chalcone, which they must analyze collaboratively using each of the techniques available at the different stations. The rotation through the stations will be carried out following the pre-established order in the following table, strictly respecting the time assigned to each activity:

Table #1 Rotation Order – Initial Practice: Characterization of Ferrocenyl Chalcones

Group 1	Group 2	Group 3	Group 4
Station 1	Station 2	Station 3	Station 4
Station 2	Station 3	Station 4	Station 5
Station 3	Station 4	Station 5	Station 6
Station 4	Station 5	Station 6	Station 7
Station 5	Station 6	Station 7	Station 1
Station 6	Station 7	Station 1	Station 2
Station 7	Station 1	Station 2	Station 3

Upon arriving at a station, it is essential that all group members carefully review the corresponding instrument's Standard Operating Procedure (SOP) and the specific instructions provided. During the session, the group must diligently complete data tables, collaboratively answer questions, and record any relevant observations, both for the standard analyses and for the unknown ferrocenyl chalcone sample. Once the allotted time has elapsed, the group is expected to move to the next station, ready to apply the knowledge acquired and continue with the comprehensive characterization of their ferrocenyl chalcone. The instructor will supervise the rotation and will be available to answer questions and help each group at each station.

STATION I: HIGH PERFORMANCE LIQUID CHROMATOGRAPHY (HPLC/UV-Vis) – Caffeine Calibration, TLC Prediction, and Ferrocenyl Chalcone Purity Assessment

Objective: High-performance liquid chromatography (HPLC) is essential for separating and analyzing complex mixtures. In this station, we will first verify the performance of the HPLC/UV-Vis system with caffeine. We will then predict the separation of the unknown ferrocenyl chalcone using TLC, and finally, we will use HPLC to assess its purity, comparing the results of both techniques.

Procedure:

1. **Instrument Preparation:** Turn on the HPLC and wait 10 minutes. Refer to the SOP.
2. **Caffeine Calibration:**
   a) Prepare a caffeine solution of known concentration. (Refer to the instructor.)

   b) Adjust the HPLC method for caffeine. (Refer to SOP)

   c) Inject the caffeine solution in triplicate.

   d) Construct the calibration curve by analyzing the solutions provided by your instructor (peak area vs. concentration), determine the equation of the calibration curve, and calculate the R^2 value.

3. **Thin Layer Chromatography (TLC) for Separation Prediction (Simulating C18 Reversed Phase Conditions):**

   a) **Plate Preparation:** Use a commercially available reversed-phase modified silica gel plate (RP-18). If not, use a standard silica gel plate.
   b) **Sample Preparation:** Dissolve a small amount of the unknown ferrocenyl chalcone in acetonitrile (or other solvent if solubility is low).
   c) **Elution:** Place a small aliquot of the solution on the plate and elute with a mixture of hexane and ethyl acetate (8:2).
   d) **Visualization:** Observe the spots under UV light.
   e) **Rf Calculation:** Measure and calculate the Rf value for each spot.
   f) **Analysis:** Use R_f values to predict the elution order of components in HPLC analysis. Remember to consider the polarities used for the mobile and stationary phases in both techniques.

Compound Order for TLC: _____ > _____ >_____>_____
Compound Order for HPLC: _____ > _____ > _____>_____

4. **HPLC Analysis of Ferrocenyl Chalcone:**

   a) **Sample Preparation**: Dissolve 5 mg of the ferrocenyl chalcone in 10 mL of methanol (concentration 0.5 mg/mL). Filter the resulting solution using a syringe filter with a pore size of 0.22–0.45 µm.
   b) **Mobile Phase Selection:** Based on the TLC results, select a binary mobile phase composed of water (A) and methanol (B)
   c) **Elution Gradient:** Program the HPLC to run the following linear gradient:

Time (min)	A (%) Aqueous Phase	B (%) Organic Phase
0	50	50
4	40	60
6	30	70
10	10	90
15	50	50

   d) **Injection and Recording:** Inject 10 µL of the filtered ferrocenyl chalcone solution into the HPLC and record the chromatogram at a wavelength suitable for ferrocene detection (typically 254 nm or 280 nm). **Note:** Record the method parameters used (mobile phase, gradient, flow rate, wavelength, etc.) on the report sheet.

5. **Analysis and Interpretation of the Results:**

   a) **Peak Identification:** Identify the principal peak that corresponds to the ferrocenyl chalcone, based on its area and retention time.
   b) **Impurities Determination:** Count the number of peaks different from the principal one on the chromatogram. Each one of these peaks represents a possible impurity.
   c) **Comparison with the TLC:** Compare the number and elution order of the peaks in the chromatogram with the TLC results.
   d) **Evaluation of Purity:** Estimate the purity of the ferrocenyl chalcone calculating the percentage of the area of the principal peak with respect to the total area of all the peaks in the chromatogram.

$$Purity\ (\%) = \left(\frac{Principal\ Peak\ Area}{Total\ Area\ of\ the\ Peaks}\right) x\ 100$$

Data Recording and Analysis:

Table #2: Caffeine Calibration

Injection #	Caffeine Concentration (ppm)	Peak Area
1		
2		
3		
Calibration Curve Equation:		
Correlation Coefficient (R^2):		

Table #3: TLC Results of Unknown Ferrocenyl Chalcone

Solvent Used (Proportion)	Spot #	Rf
[Solvent]		
Prediction of Elution Order in HPLC:		

Table #4: HPLC Chromatography Results of Unknown Ferrocenyl chalcone

Peak #	Retention Time (min)	Relative Area (%)	Identification (if possible)
1			
2			
Impurities Detected:			
Estimated Purity:			

Questions:

1. What does the R^2 value indicate about the performance of HPLC?
2. How many components did the TLC predict?
3. Do TLC and HPLC results agree in peak number and order? What advantages/disadvantages does TLC have compared to HPLC?
4. Is the sample pure? How many impurities are present?
5. What could be the sources of error in this experiment?
6. How can you determine the identity of the impurity using TLC and using HPLC?

STATION II: UV-Vis SPECTROSCOPY – Stability Verification and UV-Vis Characterization

Objective: Evaluate the stability of the spectrophotometer by verifying the suitability of the system (System Suitability Check) using potassium dichromate solutions and apply the technique to obtain and analyze the UV-Vis spectrum of unknown ferrocenyl chalcones.

Procedure:

1. **Instrument Preparation:** Turn on the UV-Vis spectrophotometer 15 minutes before starting the practice to ensure stable equipment. Refer to the instrument's Standard Operating Procedure (SOP) for detailed instructions on initial setup and operation.

2. **Preparation of Potassium Dichromate Solutions (**System Suitability Check):

   a) **Solution A:** Dissolve (25.2 ± 0.5) mg of potassium dichromate in 0.005 M sulfuric acid solution and dilute to 500 mL of solution in a volumetric flask. Record the exact mass used.
   b) **Solution B:** Dissolve (50.4 ± 0.5) mg of potassium dichromate in 0.005 M sulfuric acid solution and dilute to 500 mL of solution in a volumetric flask. Record the exact mass used.

Caution: Use volumetric flasks and calibrated pipettes to ensure accurate solution preparation. Record the exact mass used.

3. **Acquisition of Potassium Dichromate Spectra:**

   a) Turn on the spectrophotometer lamp and allow it to stabilize.

   b) Set the spectrophotometer to perform a spectral scan in the wavelength range of 220-360 nm.

      1. Use a clean quartz cuvette and 0.005 M sulfuric acid as a blank to calibrate the instrument's zero.

      2. Measure the absorption spectrum of Solution A. Save the spectrum.

      3. Measure the absorption spectrum of Solution B. Save the spectrum.

Note: Ensure the cuvette is cleaned thoroughly between each measurement to prevent cross-contamination.

4. **Preparation of the Unknown Ferrocenyl Chalcone Solution:**
   a) Dissolve a small amount of the unknown ferrocenyl chalcone in an appropriate solvent (acetonitrile or methanol) to obtain a solution with an absorbance between 0.5 and 1.0 in the wavelength range of interest. If the sample does not dissolve readily, gently warm it and/or use a different solvent. Record the solvent used.

Precaution: The concentration of the ferrocenyl chalcone must be sufficient to obtain a clear signal, but not so high as to cause detector saturation. Furthermore, the cuvette and/or solutions must be covered when not in use to prevent exposure to light.

5. **Acquisition of UV-Vis Spectrum of Unknown Ferrocenyl Chalcone:**

   a) Transfer the chalcone solution to a clean quartz cuvette.

b) Scan the absorption spectrum within an appropriate range (200–700 nm), adjusting the instrument parameters to achieve optimal measurement.

c) Record the spectrum, including the maximum wavelength (λ_{max}) and the absorbance at that wavelength.

Note: Ensure that the solvent used for the sample is transparent in the wavelength range of interest.

6. **Analysis and Interpretation of Results:**

a) **Potassium Dichromate (System Suitability):** Compare the spectra of solutions A and B. Determine the absorbance value of each solution at 235, 257, 313, and 350 nm. Compare your results with the values provided by the Instructor. Calculate the molar concentration of each solution based on the mass used and the final volume.

b) **Unknown Ferrocenyl Chalcone:** Determine the wavelength of maximum absorption (λ_{max}) of the ferrocenyl chalcone. Compare the shape of the spectrum obtained with reference spectra of similar ferrocenyl chalcones that are available. Are there any significant similarities or differences? Why are there multiple absorption peaks in the chalcone spectrum? To which electronic transition does each peak correspond?

Data Recording and Analysis:

Table #5: Preparation of Potassium Dichromate Solutions

Solution	Mass of $K_2Cr_2O_7$ (mg)	Final Volume (mL)	Molar Concentration (Calculated)
A			
B			

Table #6: Absorbance of Potassium Dichromate Solutions

Wavelength (nm)	Absorbance (Solution A)	Absorbance (Solution B)
235		
257		
313		
350		

Table #7: UV-Vis Spectral Data of Unknown Ferrocenyl Chalcone

Parameter	Value
Solvent Used	

Maximum Wavelength (λ_{max}), nm	
Absorbance at λ_{max}	

Questions:

1. What are the concentrations (in M) of solutions A and B of potassium dichromate according to your results? How does the spectrum of both solutions compare to each other? What solution would be more appropriate for a precise calibration of the instrument?
2. What is the wavelength of maximum absorption (λ_{max}) of the unknown ferrocenyl chalcone? How does the shape of the obtained spectrum compare with reference spectra of similar chalcones? Based on this comparison, is the analyzed sample pure?
3. What type of information can be obtained from the UV-Vis Spectrum of the unknown ferrocenyl chalcone? What are the limitations of this technique for its characterization?
4. What precautions must be taken when preparing solutions and making measurements to ensure the precision and accuracy of the results?
5. How can the UV-Vis be used in combination with other analytical techniques, such as chromatography, to obtain a more comprehensive characterization of the unknown ferrocenyl chalcone?

STATION III. FLUORESCENCE - Fluorescence Characterization

Objective: Understand the fundamental principles of fluorescence spectroscopy using quinine as a model compound. Learn how to optimize instrument settings and interpret data.

Procedure:

1. **Instrument Preparation:** Turn on the spectrofluorometer at least 30 minutes before use to allow it to warm up. This is crucial for obtaining stable readings. Read the SOP carefully.
2. **Blank Preparation:** Prepare a blank solution using the same solvent as your quinine sample (0.05 M sulfuric acid). This blank will correct for background signals from the solvent.
3. **Quinine Solution Preparation:** Prepare a 2.5 ppm quinine solution in 0.05 M sulfuric acid. Ensure the quinine is completely dissolved before proceeding.
4. **Acquire the excitation spectrum:**
   a) Set the emission wavelength (λ_{em}) to 450 nm. This is the wavelength at which the emitted light will be monitored.
   b) Scan the excitation wavelength (λ_{ex}) from 300 to 600 nm. Record the intensity at each wavelength.
   c) **Important**: Handle the cuvettes carefully, touching only the frosted sides of the cuvettes. Fingerprints can interfere with readings; always wear gloves to prevent this.

5. **Acquire the emission spectrum:**
   a) Set the excitation wavelength (λ_{ex}) to the maximum excitation wavelength found in the previous step.
   b) Scan the emission wavelength (λ_{em}) from 300 to 600 nm.
6. **Overlay the spectra:**
   a) Using appropriate software, plot both the excitation and emission spectra on the same graph. Write the wavelengths of maximum absorbance and maximum emission.

Quality Control: The excitation spectrum should resemble the UV-Vis absorbance spectrum of quinine.

7. **Effects of Sensitivity**: Using the optimized excitation wavelength, generate emission spectra using the "high" and "low" sensitivity settings on the instrument. Observe the effect on the signal-to-noise ratio.

8. **Effects of Scanning Speed:** Generate emission spectra at "fast" and "slow" scan speeds. Observe how the scan speed affects the resolution and intensity of the spectrum.

After calibration and analysis of quinine are complete, dissolve a small amount of the unknown ferrocenyl chalcone in an appropriate solvent and determine its emission spectrum. Compare the obtained spectrum with that of quinine and note any significant differences. Does the ferrocenyl chalcone show detectable fluorescence under the same conditions as quinine? If not, what could explain this difference?

9. **Data Analysis:** Complete the data tables below and discuss your results in the report.

Data Recording and Analysis:

Table #8: Excitation and emission spectra of quinine

Parameter	Value, Units
Maximum excitation wavelength (λ_{ex}), nm	
Maximum emission wavelength (λ_{em}), nm	
Intensity in λ_{em} (arbitrary units)	

Table #9: Effect of sensitivity on the emission spectrum

Parameter	High sensibility	Low sensibility
Maximum emission wavelength (λ_{em}), nm		
Intensity (arbitrary units)		
Signal/Noise Relationship (estimation)		

Table #10: Effect of scanning speed on the emission spectrum

Scanning Speed (nm/s)	Maximum emission wavelength (λ_{em}), nm	Intensity (arbitrary units)
Fast		
Slow		

Questions:

1. How does a faster scan speed affect the resolution of the emission spectrum?
2. Why is it important to choose the adjustment of sensitivity appropriate for the fluorescence measurements?
3. Compare and contrast the processes of absorption, excitation, and emission, illustrated with a Jablonski diagram.

STATION IV: FTIR SPECTROPHOTOMETRY – Identification of Functional Groups and Structural Confirmation

Objective: Fourier transform infrared spectrophotometry (FTIR) is an invaluable technique for identifying the functional groups present in a molecule. These functional groups absorb infrared radiation at characteristic frequencies, generating a spectrum that acts as a fingerprint of the molecule. In this station, you will learn how to operate the FTIR spectrometer, prepare samples, obtain and interpret spectra, and confirm the presence of the expected functional groups in the unknown ferrocenyl chalcone.

Procedure:

1. **Instrument Preparation:** Turn on the FTIR spectrometer and allow it to warm up for at least 30 minutes. Refer to the instrument's Standard Operating Procedure (SOP) for detailed instructions.
2. **Instrument Calibration:** Perform calibration of the FTIR spectrometer using polystyrene films. Record the positions of the characteristic polystyrene peaks and compare them with reference values to verify instrument accuracy. Refer to the SOP for the calibration procedure.
3. **Sample Preparation:** Prepare a sample of the unknown ferrocenyl chalcone for FTIR analysis. Depending on the instrument type and available accessories, this may involve:
   a) **ATR (Attenuated Total Reflectance):** Place a small amount of the ferrocenyl chalcone directly onto the ATR crystal.

b) **KBr (Potassium Bromide):** Mix a small amount of the ferrocenyl chalcone with dry KBr and press the mixture into a clear pellet.

Note: Ensure the sample is dry to avoid moisture interference in the spectrum.

4. **FTIR Spectrum Acquisition:** Place the prepared sample in the FTIR spectrophotometer and acquire the spectrum in the range of 4000 to 400 cm^{-1}. Record the spectrum in a suitable format for subsequent analysis.
5. **Analysis and Interpretation of the Results:**
   a) **Peak Identification:** Identify the most prominent peaks in the FTIR spectrum of the unknown ferrocenyl chalcone. Consult functional group correlation tables to assign these peaks to specific chemical bond vibrations (e.g., C=O, C-H, C=C, etc.).
   b) **Functional Group Confirmation:** Compare the observed peaks with the expected functional groups in the structure of the ferrocenyl chalcones (e.g., cyclopentadienyl ring, carbonyl group, aromatic substituents). Determine if the spectrum confirms the presence of these functional groups.
   c) **Impurity Analysis:** Evaluate whether there are additional peaks in the spectrum that cannot be assigned to the expected structure of the ferrocenyl chalcone. These peaks could indicate the presence of impurities. Additionally, use the correlation tables to evaluate the peak intensities of the most prominent functional groups.

Data Recording and Analysis:

Table #11: Calibration of the FTIR Spectrometer

**Standard: Polystyrene Film**	**Theoretical Peak Position (cm^{-1})**	**Experimental Peak Position (cm^{-1})**
Peak 1		
Peak 2		
Peak 3		

Table #12: Identification of Functional Groups in the Unknown Ferrocenyl Chalcone

**Theoretical region (cm^{-1})**	**Vibrational assignment / Functional group**	**Involved chemical bond**	**Observed value (cm^{-1})**	**Experimental intensity**	**Comment**
3100 – 3000	Stretch C–H aromatic (Cp and phenyl)	Aromatic C–H			

Theoretical region (cm^{-1})	Vibrational assignment / Functional group	Involved chemical bond	Observed value (cm^{-1})	Experimental intensity	Comment
1660 – 1650	C=O stretch conjugated with C=C	Conjugated C=O			
1600 – 1580	Aromatic C=C Stretch (phenyl)	Aromatic C=C			
1550 – 1500	C=C stretching of the Cp ring	C=C (Cp)			
1100 – 1000	Out-of-plane C–H bending (Cp and phenyl)	C–H (out-of-plane)			
870 – 820	Characteristic vibrations of the Cp ring	C–H (Cp, ferrocene)			
500 – 480	Fe–C stretching (ferrocene diagnostics)	Fe–C			

Questions:

1. According to the results obtained with the polystyrene, is the FTIR Spectrophotometer calibrated correctly? Justify your answer.
2. Based on the FTIR spectrum, what functional groups can you identify in the unknown ferrocenyl chalcone? Are these functional groups consistent with the structure of the compound?
3. Are there peaks in the FTIR that do NOT correspond to the expected structure? If there are, what could these peaks indicate?
4. How can you use the information obtained with FTIR in combination with the results of other analytical techniques (HPLC or NMR) to confirm the structure of an unknown ferrocenyl chalcone?
5. What are the limitations of FTIR Spectroscopy for characterizing organic compounds?

STATION V: NUCLEAR MAGNETIC RESONANCE (NMR) – Structural Confirmation and Purity Assessment

Objective: Nuclear Magnetic Resonance (NMR) spectroscopy is a powerful technique for determining the structure and purity of organic compounds. NMR spectra provide detailed information about the chemical environment of different atoms in a molecule, allowing for the identification of functional groups, determination of atomic connectivity, characterization of the chemical environment surrounding nuclei, and quantification of the components in a mixture. In this station, you will learn how to prepare samples for NMR analysis, acquire and interpret ^{1}H-NMR and ^{13}C-NMR spectra, assign observed signals to specific atoms in the unknown f ferrocenyl chalcone, and assess its purity.

Procedure:

1. **Instrument Preparation:** Turn on the NMR spectrometer and allow it to stabilize. Refer to the instrument's Standard Operating Procedure (SOP) for detailed instructions.
2. **Sample Preparation:** Dissolve approximately 5–10 mg of the unknown ferrocenyl chalcone in 0.75 mL of an appropriate deuterated solvent (e.g., $CDCl_3$, DMSO-d_6). Ensure the sample is completely dissolved and transfer the solution to a 5 mm diameter NMR tube. Add a few drops of tetramethylsilane (TMS) as an internal reference (some deuterated solvents already include this reference). **Note 1**: When not analyzing your sample, cover it with aluminum foil or keep it away from light. **Note 2**: It is crucial to use a deuterated solvent to avoid solvent signals in the ^{1}H-NMR spectrum.
3. **NMR Spectra Acquisition:** Acquire a ^{1}H-NMR spectrum of the unknown ferrocenyl chalcone. Adjust instrument parameters (spectral width, acquisition time, number of scans, etc.) to obtain a high-quality spectrum with a good signal-to-noise ratio.
   Note: Consult your instructor or the instrument's SOP for instructions on optimizing NMR spectral acquisition parameters.
4. **NMR Spectra Processing:** Utilize the NMR spectrophotometer software to process the acquired spectra, including baseline correction, phase adjustment, chemical shift scale calibration using the TMS signal, and integration of each peak.
5. **Analysis and Interpretation of Results:**
   a) Before analyzing your spectrum, you should look at the unknowns in Figure 2 and predict which peaks you would expect to observe. Consider whether the substituents on the aromatic ring would produce any signals.

b) Determine the chemical shift (in ppm) of each signal in the ^{1}H-NMR spectrum. You can use the software to assist you.

c) Determine the multiplicity (singlet, doublet, triplet, quartet, multiplet), the coupling constant (in Hz) for the split peaks, and the integration for each peak.

d) Integrate the areas under each signal to determine the relative number of hydrogen atoms contributing to each signal.

e) Assign each signal to a specific hydrogen atom in the structure of the ferrocenyl chalcone, based on its chemical shift, integration, multiplicity, and coupling constant.

f) Assign each signal to a specific carbon atom in the structure of the ferrocenyl chalcone, based on its chemical shift and the information obtained from the ^{1}H-NMR spectrum.

g) Analyze the number of observed signals and their relative intensity to determine the symmetry of the molecule.

6. **Purity Assessment:** Determine if there are additional signals in the NMR spectrum that cannot be assigned to the expected structure of the ferrocenyl chalcone. If detected, these signals could indicate the presence of impurities. Estimate the purity of the ferrocenyl chalcone by comparing the integration of the peaks corresponding to the main product with the integration of the peaks corresponding to the impurities.

Data Recording and Analysis:

Table #13: ^{1}H-NMR Spectral Data of Unknown Ferrocenyl Chalcone

Chemical Shift (ppm)	Multiplicity	Coupling Constant (Hz)	Integration	Proton-Peak Assignment

Table #14: Purity Evaluation

Signal	Relative Area (%)	Does it correspond to the Main Product?	Does it correspond to an impurity?
Estimated Purity (based on NMR):			

Questions:

1. What is the molecular formula and molecular weight of the unknown ferrocenyl chalcone? What information did you use to determine these values?

2. In the ^{1}H-NMR spectrum, which signals correspond to the hydrogen atoms in the cyclopentadienyl ring? How do you justify the peak assignment based on chemical shifts and the signal multiplicity? How do the chemical shifts of these aromatic hydrogens from the cyclopentadienyl ring compare with other aromatic hydrogens? Explain the difference in shifts (Review the concept of anisotropy).

3. What substituents are present in the unknown ferrocenyl chalcone as determined by the ^{1}H NMR? What is the position of the substituent in the aromatic ring? How do you justify the assignment of peaks based on the chemical shifts, splitting, and signal coupling patterns?

4. According to the NMR spectrum, what is the purity of the unknown ferrocenyl chalcone? What are the possible impurities you have identified in the sample?

5. What are the limitations of NMR for characterizing organic compounds? What type of additional information could be obtained with other spectroscopy or chromatography techniques?

STATIONS VI and VII Thermal Analysis – Differential Scanning Calorimetry (DSC) and Thermogravimetric Analysis (TGA)

Objective: Thermal Analysis, which includes Differential Scanning Calorimetry (DSC) and Thermogravimetric Analysis (TGA), is a set of techniques that assess the physical and chemical properties of a material as a function of temperature. DSC measures the heat flux associated with thermal transitions, such as melting or the glass transition. In contrast, TGA measures changes in a sample's mass as it is heated, providing information about its thermal stability and the extent of decomposition. At this station, you will learn how to calibrate the DSC instrument

using indium, and then use both techniques to assess the purity, thermal stability, and degradation patterns of the unknown ferrocenyl chalcone.

Procedure:

1. **DSC Instrument Calibration:** Turn on the DSC instrument and allow it to stabilize. Refer to the instrument's Standard Operating Procedure (SOP) for detailed instructions on powering up, configuration, and operating the instrument safely.

2. **Indium Calibration:** Perform DSC calibration using indium as the standard. Follow the steps below:

   a) Place a small piece of metallic indium (certified purity) in a clean, dry aluminum crucible.

   b) Program the DSC to perform a temperature sweep from 25° C to 350 °C at a heating rate of 15 °C / min (10 °C / min ramp).

   c) Record the indium melting temperature (T_m) and heat of fusion (ΔH_{fus}).

   d) Compare the measured values with the certified values for indium. Adjust the DSC calibration as necessary, following the SOP instructions.

Note: Ensure the DSC cell is purged with an inert gas (e.g., nitrogen) during calibration and analysis to prevent sample oxidation.

3. **Preparation of the Unknown Ferrocenyl Chalcone Sample:**

   a) Carefully weight 2 to 5 mg of the unknown ferrocenyl chalcone and place it in a clean, dry aluminum crucible.

   b) Register the exact mass used.

Caution: The sample mass must be sufficient to obtain a clear signal, but not so considerable as to cause heat transfer problems.

4. **DSC Analysis of Unknown Ferrocenyl Chalcone:**

   a) Place the crucible with the sample in the DSC.

   b) Program the DSC to perform a temperature sweep from 25 °C to 350 °C at a heating rate of 15 °C / min.

   c) Record the DSC curve, including temperature and heat flux.

5. **TGA Analysis of Unknown Ferrocenyl Chalcone:**

    a) Place the crucible with the sample (ideally, the same sample used for the DSC) in the TGA.

    b) Program the TGA to perform a temperature sweep from 25 °C to 350 °C at a heating rate of 15 °C / min.

    c) Record the TGA curve, including temperature and mass loss.

6. **Analysis and Interpretation of Results:**

    a) **Melting Point (DSC):** Determine the melting point (T_m) of the ferrocenyl chalcone from the DSC curve. A single, sharp peak indicates high purity, while a broad peak or multiple peaks suggest the presence of impurities.

    b) **Crystalline Purity (DSC):** Evaluate the crystal purity of the ferrocenyl chalcone by analyzing the shape and width of the melting peak on the DSC curve.

    c) **Thermal Stability (TGA):** Determine the degradation onset temperature (T_{onset}) from the TGA curve. A high T_{onset} indicates good thermal stability.

    d) **Degradation Patterns (TGA):** Analyze the shape of the TGA curve to identify possible decomposition steps. Mass loss at different temperature ranges may indicate the release of different volatile components or decomposition of different parts of the molecule.

Data Recording and Analysis:

Table #15: DSC Calibration with Indium

Parameter	Certified Value	Exp. Value
Melting Temperature (T_m), °C		
Heat of Fusion (ΔH_{fus}), J/g		
Temperature range		

Table #16: Thermal Analysis of the Unknown Ferrocenyl Chalcone

Parameter	Value
Sample Mass (mg)	
Fusion Temperature (T_m), °C	
Width of the Melting Peak (DSC), °C	
Degradation Start Temperature (T_{onset}), °C	
Total Mass Loss (TGA), %	
Temperature stage	25 °C to 350 °C

Questions:

1. What does the melting temperature obtained in DSC analysis of the unknown indicate? Compare the value with the values in the literature.

2. Does the form of the melting peak in DSC suggest that the ferrocene is pure or impure? Explain.

3. If there were more than one peak in the DSC curve, explain what would be the effect on the melting point when a soluble impurity is present in the sample analyzed? What would be the effect if the impurity were insoluble?

4. At what temperature does the ferrocenyl chalcone start to degrade, as determined by the TGA analysis? Is this temperature reasonable, considering the structure of the compound?

5. What information about the decomposition of the ferrocenyl chalcone can be obtained from the TGA curve?

6. How do the results in DSC and TGA compare to each other? Do they complement each other to have a more complete characterization?

7. What are the possible errors of this experiment? How could you optimize the precision of the measurements?

FINAL INSTRUCTIONS

At the end of the practice, each student or group must organize the information obtained into **three complementary products**:

1. **Results Integration Sheet:** This should be completed first and will serve as an organized record of all raw experimental data generated in the lab.

2. **Technical data sheet for the assigned compound:** based on the integration sheet, create a brief and precise data sheet that includes the name of the chalcone, its structure, the most relevant spectroscopic data (UV-Vis, FTIR, NMR), the purity parameters (HPLC, DSC, TGA), and the main observations on the compound's stability or properties.

3. **Laboratory report in ACS format:** Use the information summarized in the data sheet to write a formal report with the following structure.

   - **Title** (concise and clear).

   - **Authors** (Name of students and group).

   - **Summary** (150–200 words with objectives, techniques, key results, conclusions).

- **Introduction** (context, relevance in materials chemistry, aim of the work).
- **Experimental Methodology Summarized** (synthesis, sample preparation, and the instruments used).
- **Results and Discussion** (tables, figures, critical analysis comparing experimental data with literature data and AI predictions).
- **Conclusions** (identity, purity, and contributions of the practice).
- **References** (in ACS format).

As a **report appendix**, all the analysis questions proposed throughout the manual should be included and thoroughly answered.

In summary, the expected workflow is as follows: **first**, complete the integration sheet as a database of experimental data; **then**, create the chalcone's technical data sheet from that information; and **finally**, write the full report in ACS format, applying scientific language and structure as used in the chemical community.

Results Integration Sheet – Ferrocenyl Chalcones

Name: ______________________________
Assigned Sample (A–H): _____________________________

1. Summary of Experimental Data

Complete the table with the values obtained in each technique:

Technique	Experimental Data	Observations	What information does it provide about the sample?
UV-Vis / Fluorescence	λ_{max} Bands I–IV: _____________<br>Emission _____________<br>Excitation		
FTIR	Characteristics peaks:<br>_____________		
^{1}H NMR	δ Shifts (ppm): _________<br>Integration: ________<br>J Coupling: _________		
^{13}C NMR	δ Shifts (ppm): _____________		
HPLC	Principal t_R: _____________<br>Purity %: _____________		
DSC	T_m (°C): _____________<br>Peak form: _____________		
TGA	Onset degradation: __________<br>Loss %: _____________		

2. Chalcone Identity

a) Which ferrocenyl chalcone (A–H) corresponds to your sample?
b) Indicate which spectra or signals were decisive in your conclusion (e.g., FTIR band, NMR shift, UV-Vis absorption).

Response:

____
____
____

3. Purity Assessment

a) Contrast HPLC, DSC, and NMR: Do they agree in indicating a pure material? If there are discrepancies, how would you explain them?

Response:

___

___

___

4. Consistency Between Techniques

a) Does your spectroscopy data (UV-Vis, FTIR, NMR) agree with the chromatographic and thermal results?
b) Give an example of a precise match and a possible discrepancy.

Response:

___

___

___

5. Reflection on GLP and QA/QC

a) What standards did you use to verify instrument calibration?
b) Explain how these verifications provide confidence in your data.

Response:

___

___

___

6. Integrative Conclusion:

a) Summarize the identity and purity of your sample in 3–4 sentences.
b) Indicate which technique you consider the most decisive and why.

Response:

___

___

___

TECHNICAL SHEET – FERROCENE CHALCONE

Name: ___________________________ **Assigned Sample (A–H):**____________

Sample Identity:

- **IUPAC Name:** ____________________________
- **Structure:** (insert image)
- **Molecular Formula:** ___________________
- **Molecular weight (g/mol):** _________________

Instrumental Characterization:

Technique	Experimental Data	Theoretical Values	Observations
UV-Vis	λ_{max}: ___________	200–240 nm<br>300–360 nm<br>360–480 nm	____________________
FTIR	Peaks:<br>_____________	1650–1660 (C=O) 480–500 (Fe–C) etc.	____________________
¹H NMR	δ (ppm):<br>_____________	Cp: 4–5 ppm, vinylic: 7–8 ppm	____________________
HPLC	t_R: ______ min / Purity: ______ %	Standard > 95 %	____________________
DSC	T_m: ______ °C	Reference ______ °C	____________________
TGA	Onset: ____ °C	Stability >250 °C	____________________

Featured Properties:

- **Observed Color:** ___________
- **Physical State:** ___________
- **Relative Purity:** High ☐ Medium ☐ Low ☐
- **Thermal Stability:** ___________

Researcher's Notes:

- **Key results confirming identity:**
  ____
- **Possible impurities or deviations:**
  ____
- **Comparison with AI predictions:**
  ____

SLF_SupportingInformation_3_ExperimentalSetupAndCalibrationProcedures

Experimental Setup and Calibration Procedures for Instructors

Instructor Overview

Upon completion of this experiment, students will:

- Apply Good Laboratory Practices (GLP) and Quality Assurance/Quality Control (QA/QC) principles.
- Operate and calibrate UV–Vis, Fluorescence, FTIR, NMR, HPLC, DSC, and TGA instruments.
- Evaluate purity and identity of ferrocenyl chalcone derivatives through cross-technique data integration.
- Construct professional data reports aligned with ACS formatting standards.

Instructor Tip: Begin the session by reviewing the calibration logic for each technique (use standard materials: caffeine, quinine, polystyrene, indium, etc.). Emphasize to students that calibration validates the analytical quality of their data.

List of Equipment / Materials

- ❖ MEL-TEMP
- ❖ Alcohol Thermometer
- ❖ Melting point capillary tubes
- ❖ Aluminum foil
- ❖ Silica gel Thin Layer Chromatography [Aluminum support; fluorescence indicator 254 nm]
- ❖ TLC capillary tubes
- ❖ Beakers
- ❖ Glass watches
- ❖ Ruler
- ❖ Volumetric flasks (10 mL, 25 mL, 100 mL)
- ❖ Quartz cuvettes
- ❖ Pasteur Pipettes
- ❖ Volumetric Pipettes (1 mL)
- ❖ HPLC vials and caps
- ❖ Test tubes
- ❖ NMR Sample tubes (5 mm o.d.) and caps
- ❖ Delicate task wipes
- ❖ Aluminum crucibles and lids
- ❖ Crucible sealing press
- ❖ Tweezers
- ❖ Hexane
- ❖ Ethyl Acetate
- ❖ Methanol
- ❖ Deuterated chloroform $CDCl_3$
- ❖ Standard samples (specific for each method mentioned below)

- Synthesized pure chalcones
  - 3-(phenyl)-1-ferrocenyl-2-propen-1-one (unsubstituted phenyl)
  - 3-(2-Methoxyphenyl)-1-ferrocenyl-2-propen-1-one (o-OCH_3)
  - 3-(4-Methoxyphenyl)-1-ferrocenyl-2-propen-1-one (p-OCH_3)
  - 3-(3,4-Dimethoxyphenyl)-1-ferrocenyl-2-propen-1-one (3,4-di-OCH_3)
  - 3-(4-Nitrophenyl)-1-ferrocenyl-2-propen-1-one (p-NO_2)
  - 3-(3-Clorophenyl)-1-ferrocenyl-2-propen-1-one (m-Cl)
  - 3-(4-Chlorophenyl)-1-ferrocenyl-2-propen-1-one (p-Cl)
  - 3-(4-Bromophenyl)-1-ferrocenyl-2-propen-1-one (p-Br)

Experimental Organization

A. Pre-laboratory Sample Preparation

Review student lab manual Sections I–VII before class. Prepare vials of unknown ferrocenyl chalcones (~250 mg each). Include one "impure" batch (80% or 50% purity) prepared using 1-acetylferrocene. Label vials A–H according to substitution pattern and maintain a separate key accessible only to instructors.

Table 1: Possible ferrocenyl chalcones for experiment

Label	Substituent	Descriptor
A	H	Unsubstituted
B	o-OCH_3	Electron-donating
C	p-OCH_3	Position variation
D	3,4-di-OCH_3	Strong EDG
E	p-NO_2	Strong EWG
F	m-Cl	Moderate EWG
G	p-Cl	Position variation
H	p-Br	Heavy atom substitution

In our case the unknown samples that were provided to students were prepared as follows:

- Unknown sample vials were cleaned and the oven dried.
- Approximately ~ 250.0 mg of the pure *unknown* ferrocenyl chalcone sample were added into a vial. This amount was enough for two lab sections (4 groups of 2-3 students per section)
- For some samples, 1-acetylferrocene was included as an "impurity" to simulate a sample that had starting material (e.g., reaction was not completed, or purification was not done properly). This can apply to any of the unknown samples of ferrocenyl derivatives.

  - "Impure" Sample A

        - 200.0 mg of ferrocenyl chalcone and 50.0 mg of 1-acetylferrocene were dissolved with ethyl acetate / acetone in a round bottom flask until a homogeneous solution was produced and solvent was removed under rotary evaporation. This simulated an “impure” sample with 80% purity (20% impurity of a starting material).
    - “Impure” Sample B
        - 125.0 mg of ferrocenyl chalcone and 125.0 mg of 1-acetylferrocene were dissolved with ethyl acetate / acetone in a round bottom flask until a homogeneous solution was produced and solvent was removed under rotary evaporation. This simulated an “impure” sample with 50% purity (50% impurity of a starting material).
- Students used this sample to prepare solutions for the characterization techniques as described in the laboratory manual.

B. Characterization Analysis

For characterization techniques, the following procedures were followed:

1. Melting Point (MEL-TEMP)

a. ***Calibration/Preparation:*** Determination of temperature was made using calibrated alcohol thermometers.
b. ***Analysis of the Unknown:*** The melting point was determined by use of capillary tubes partially filled with sample (2-3 mm) and using alcohol thermometers for the instrument. The temperature was increased gradually.

2. TLC (support; fluorescence indicator 254 nm)

a. ***Calibration/Preparation:*** Silica gel plates and a hexane:ethyl acetate (8:2) mobile phase were used. 250 mL of mobile phase was prepared and enough for two lab sections (4 groups of 2-3 students per section). TLC plates were cut beforehand in rectangles with dimensions of 1.5 x 2.0 inches. Students made a thin line at the base of the TLC plate (~ 1 cm from the edge) with a carbon pencil to use as a reference to measure retention factor. In addition, capillary tubes were used to apply samples, and a UV light chamber was set in the lab. The idenTLC separation chamber was a beaker covered with a glass watch.
b. ***Analysis of the Unknown:*** Students dissolved a small amount of their unknown compound in the mobile phase and then applied a spot of their unknown in the TLC. After elution, they let the plate dry, evaluated under UV light, and calculated the retention factor R_f.

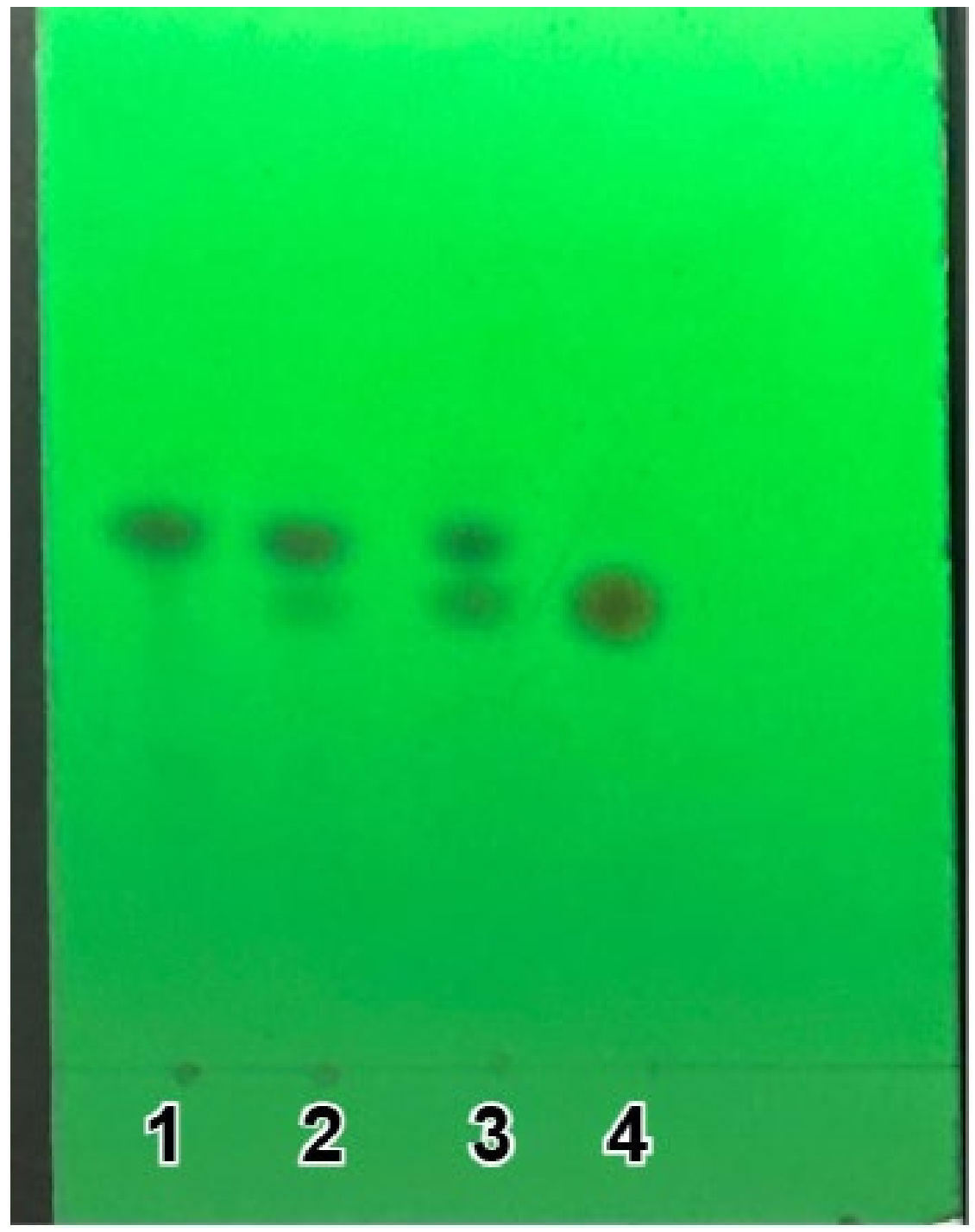

Figure 1: Example of Thin Layer Chromatography result: 1) 3-(4-bromophenyl)-1-ferrocenyl-2-propen-1-one [100%], 2) 3-(4-bromophenyl)-1-ferrocenyl-2-propen-1-one : 1-acetylferrocene [80 : 20], 3) 3-(4-bromophenyl)-1-ferrocenyl-2-propen-1-one : 1-acetylferrocene [50 : 50], 4) 1-acetylferrocene [100%]

3. HPLC/UV-Vis (Waters Alliance e2695, 2998 PDA Detector)

a. ***Calibration/Preparation:*** This calibration exercise validates retention time reproducibility, detector linearity, and method precision in the Waters Alliance e2695 system with the 2998 PDA detector. Using caffeine as a reference analyte introduces students to the process of constructing an external standard calibration curve and reinforces quantitative chromatographic principles essential for later analysis of ferrocenyl chalcones.
b. ***Critical Parameters:*** Use freshly prepared caffeine stock solution (250 ppm) in 1:1 methanol:water. Caffeine is hygroscopic; it weighs quickly and accurately. Degas all solvents and standards via ultrasonic bath (5-10 min) or vacuum filtration to prevent bubble formation in the detector flow cell. Ensure mobile phase composition is precisely 50:50 (v/v) methanol:water; both solvents must be HPLC-grade and filtered (0.22 μm). The students calibrated the instrument by determining the caffeine concentration present in a carbonated beverage using an external standard calibration curve. Initially, they prepared a sample solution of 50% methanol and 50% distilled, fully degassed water. The caffeine standard solutions were

prepared using a 250-ppm caffeine stock solution in a 100 mL volumetric flask. Using this stock solution, diluted caffeine solutions were prepared in 10 mL volumetric flasks, with caffeine concentrations of 25, 50, 75, 100, and 125 ppm. Each solution was brought to a volume of 1:1 methanol and water. Caffeine sample preparation consisted of degassing a portion of the carbonated beverage before preparing the sample. Then, they used 10.0 mL of the solution, transferring it to a 25.0 mL volumetric flask and filling it to the mark with the 1:1 methanol-water solution. Then, they transferred the standard solutions and the sample into vials for analysis. The configuration of the instrument for analysis using the isocratic method was done, placing distilled water in position A and methanol in position B. The isocratic method would distribute the mobile phase as 50% distilled water: 50% methanol throughout the analysis. Instructors guided students in the process of method preparation in the HPLC software, making sure parameters were set correctly for elution. Once the analysis was complete, the students determined the retention times and peak areas of the standard and sample solutions, graphed the peak area of the caffeine standard solutions according to the caffeine concentration, and used linear regression to determine the caffeine concentration in the sample.

c. ***Expected Results:***
   i. Retention time (t_R): 2.8 ± 0.1 min under isocratic conditions.
   ii. Calibration curve (25–125 ppm): linear with $R^2 \geq 0.995$.
   iii. Detector response should yield consistent peak symmetry (As = 0.9–1.2) and minimal baseline noise (<0.005 a.u.).
   iv. The %RSD of peak area for triplicate injections ≤ 2.0% confirms precision.
   v. The beverage sample typically yields caffeine concentrations between 40–55 ppm, depending on the brand.

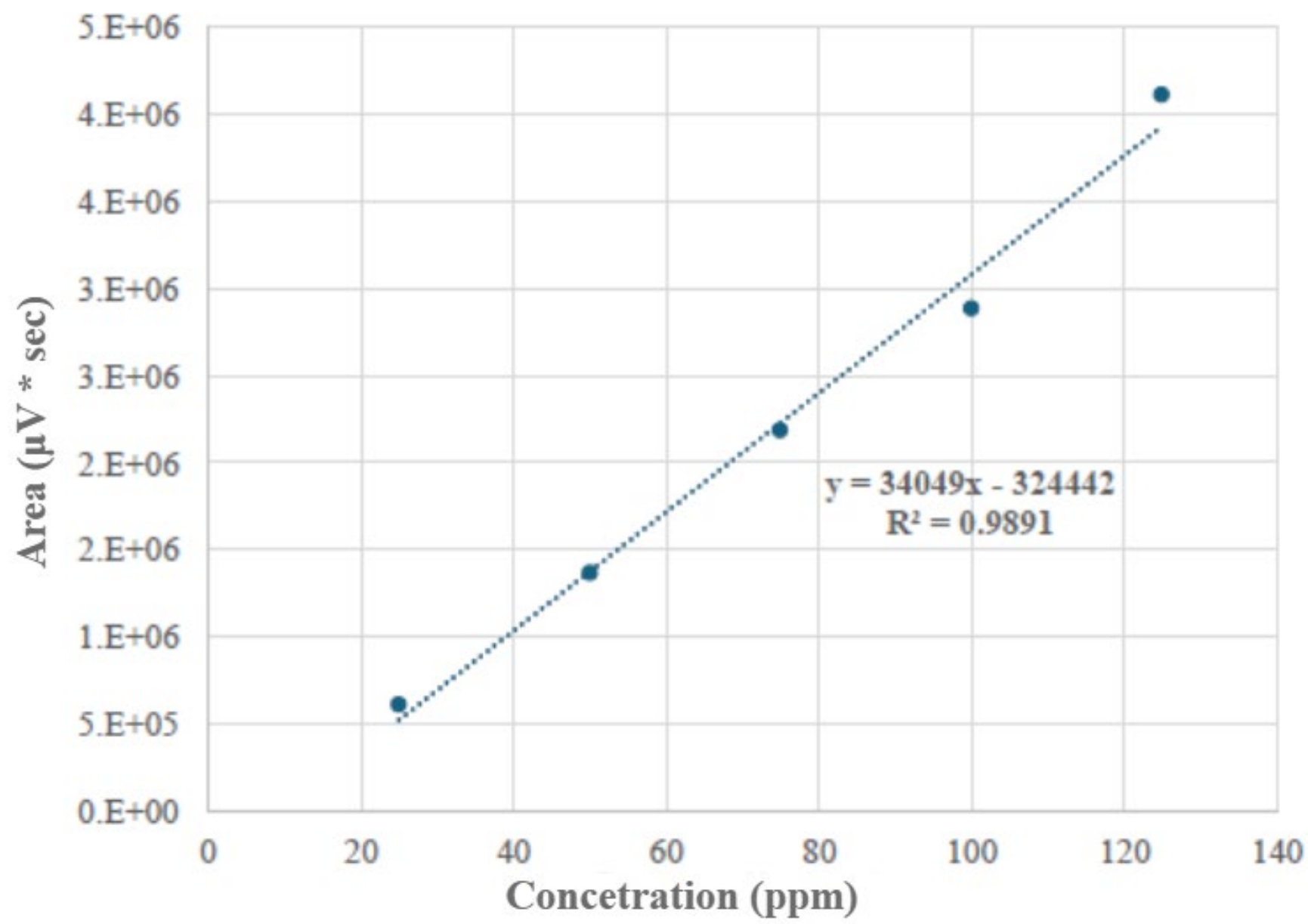

Figure 2. Example of expected calibration curve example for HPLC

d. ***Common Issues & Troubleshooting:***
   i. Baseline drift or noise: Check solvent degassing and detector lamp age. Ensure mobile phase miscibility and cleanliness of the flow cell.
   ii. Irregular peak shape: Indicates column contamination-flush with high organic solvent (methanol or acetonitrile) for 15 min.
   iii. Retention time shifts: Often caused by temperature fluctuations or pump pressure instability; ensure consistent room conditions and leak-free seals.
   iv. Nonlinear calibration curve: Verify standard preparation accuracy and avoid using old caffeine solutions (>48 h).
   v. Quality Control Indicators:
      a. %RSD (retention time) < 0.5%; %RSD (peak area) < 2.0%.
      b. Signal-to-noise ratio > 100:1 at 272 nm.
      c. Baseline stability within ±0.002 a.u. during isocratic run.

e. ***Analysis of the Unknown:*** Students prepared a stock solution by weighing 5.0 mg of their unknown and dissolving it in 10 mL of methanol (0.5 mg/mL) in a volumetric flask and filtered it with a 0.22 µm microporous membrane to remove any particulate matter before placing it in a 2 mL vial for injection. Additionally, the solutions were covered with aluminum foil when not in use to prevent exposure to light. They configured the instrument for analysis using the software Empower, through which a specific method was created. The important components to be established

are for the PDA, the wavelength range of 210-400 nm, and in the chromatography instrument, the elution gradient (table below). This gradient is based on the preparation of the mobile phase, where nanopure or distilled water was used in position A, which is degassed by the sonification method for approximately 5 minutes. In the organic phase, position B, the methanol eluent is placed, which is also degassed by the same procedure.

Table 2: HPLC configuration

<table>
<tr><th>Parameter</th><th colspan="3">Value</th></tr>
<tr><td>Instrument</td><td colspan="3">Waters Alliance e2695, 2998 PDA Detector</td></tr>
<tr><td>Analytical column</td><td colspan="3">X Bridge C18(50 * 4.6mm, 3.5 μm)</td></tr>
<tr><td>Column temperature</td><td colspan="3">room temperature</td></tr>
<tr><td>Injection volume</td><td colspan="3">10 μL</td></tr>
<tr><td>Gradient Flow Rate</td><td colspan="3">1.0 mL/min</td></tr>
<tr><td>Mobile phase</td><td colspan="3">A: Water B: Methanol</td></tr>
<tr><td>Detect wavelength</td><td colspan="3">210 nm – 400 nm</td></tr>
<tr><td>Elution Isocratic (a)</td><td colspan="3">A (50%) Aqueous Phase. B (5%) Organic Phase (Time: 5 min)</td></tr>
<tr><td rowspan="6">Elution Gradient (b)</td><td>Time (min)</td><td>A (%)
Aqueous Phase</td><td>B (%)
Organic Phase</td></tr>
<tr><td>0</td><td>50</td><td>50</td></tr>
<tr><td>4</td><td>40</td><td>60</td></tr>
<tr><td>6</td><td>30</td><td>70</td></tr>
<tr><td>10</td><td>10</td><td>90</td></tr>
<tr><td>15</td><td>50</td><td>50</td></tr>
</table>

f. ***Pedagogical Emphasis:*** Highlight to students how calibration and linear regression form the foundation of quantitative chromatography. Encourage them to interpret the meaning of R^2 as a metric of analytical reliability and to relate HPLC purity quantification to QA/QC principles later applied to ferrocenyl chalcone samples. Emphasize that a clean baseline and reproducible peaks are signs of both technical precision and scientific integrity.

4. UV-Vis (Shimadzu UV-1800 Spectrophotometer)

a. ***Calibration/Preparation:*** This calibration ensures instrument performance meets photometric accuracy standards before student use. Potassium dichromate ($K_2Cr_2O_7$) is a certified UV–Vis reference material with well-defined absorbance maxima at 235, 257, 313, and 350 nm in 0.005 M H_2SO_4.

b. ***Critical Parameters:*** Use analytical-grade $K_2Cr_2O_7$ dried at 120 °C for 2 hours prior to weighing to remove moisture. Always prepare fresh solutions, dichromate slowly photodecomposes under ambient light. Keep volumetric

flasks covered with aluminum foil. Allow the UV–Vis lamp to stabilize for at least 15 minutes to minimize baseline drift. Use matched quartz cuvettes (1.000 cm path length). Check for fingerprints and residue; clean with ethanol and lint-free wipes. between readings, rinse the cuvette with the upcoming solution twice before filling.

c. ***Expected Results:***
   i. Characteristic absorbance peaks at 235 ± 1 nm, 257 ± 1 nm, 313 ± 1 nm, and 350 ± 1 nm.
   ii. Absorbance ratio $A_{350}/A_{235} \approx 0.18\text{-}0.20$ confirms proper instrument linearity and wavelength accuracy.
   iii. Baseline deviation ≤ 0.005 a.u. across the scan confirms lamp stability.

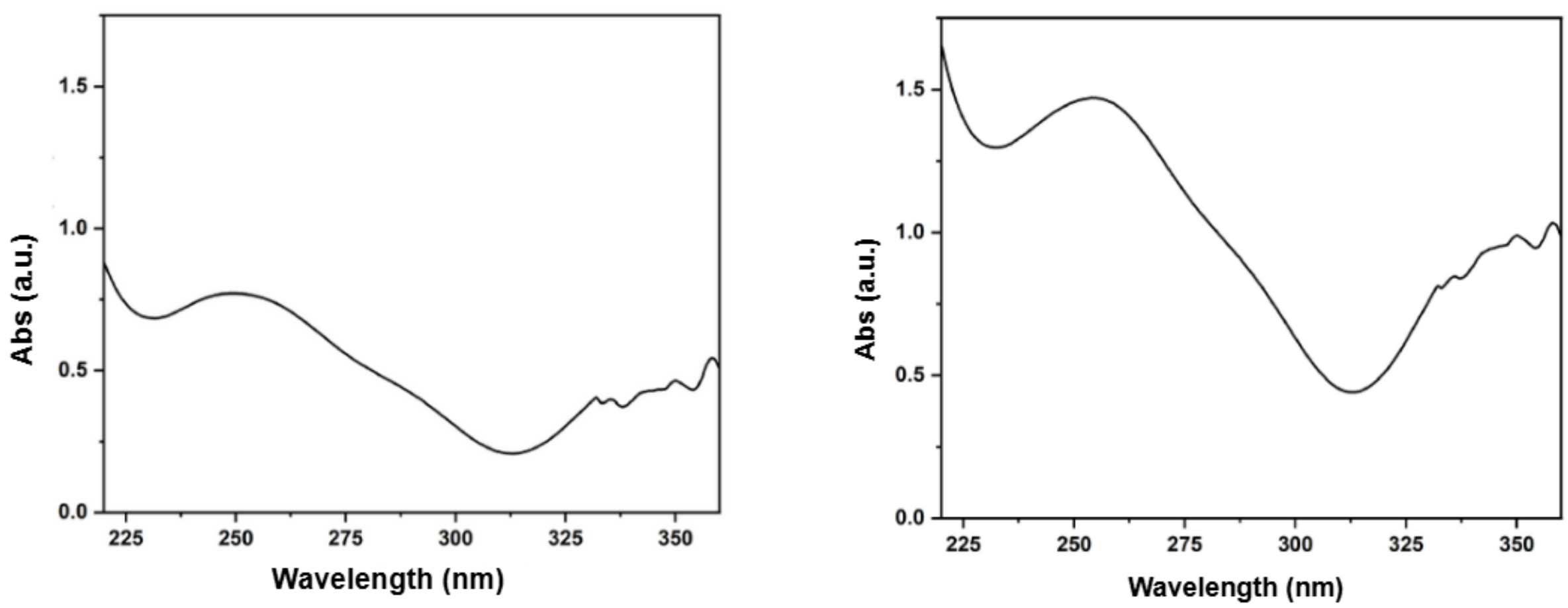

Figure 3. Example of the Potassium Dichromate UV-VIS absorption spectrum obtained by the students.

d. ***Common Issues & Troubleshooting:***
   i. Baseline noise or drift: Ensure adequate lamp warm-up and use clean cuvettes and fresh solvent blanks.
   ii. Peak shift (>2 nm): Verify wavelength calibration using a certified reference standard.
   iii. Abnormal absorbance ratios: Typically caused by contaminated cuvettes or aged potassium dichromate solutions; clean cuvettes and prepare fresh standards.
   iv. Absorbance >1.0 a.u. for unknowns: Further dilute the sample with methanol before analysis.

e. ***Analysis of the Unknown:*** Students diluted the HPLC stock solution (0.5 mg/mL) by taking a 1 mL aliquot and diluting it with methanol in a 10 mL volumetric flask (0.05 mg/mL). Solutions were covered with aluminum foil when not in use to prevent exposure to light. In the instrument, the scan was set up from 200 to 700 nm. Wipes were used to clean cuvette walls before inserting them into the instrument. Instructors are encouraged to

guide students in this process to prevent cuvette spills or breaking. If the solution resulted in an absorbance value > 1 a.u., students were guided to make further dilutions until an appropriate value was obtained.

f. ***Pedagogical Emphasis***: Guide for students to relate system suitability testing to Quality Assurance/Quality Control (QA/QC) principles. Reinforce that calibration is not merely procedural; it quantifies the instrument's analytical reliability and underpins the validity of all subsequent spectra.

5. Fluorescence (Shimadzu RF-5301pc Spectrofluorophotometer)

a. ***Calibration/Preparation:*** Quinine sulfate is a classical standard for verifying wavelength accuracy, photometric response, and sensitivity in fluorescence spectroscopy. Its well-characterized excitation (~350 nm) and emission (~450 nm) maxima provide a reliable benchmark for instrument performance and allow students to visualize the relationship between excitation and emission processes.

b. ***Critical Parameters:*** Use freshly prepared 2.5 ppm quinine sulfate in 0.05 M H_2SO_4; exposure to light and air gradually quenches fluorescence. Always prepare and measure a blank of the same solvent to remove Raman scattering and background signal. Confirm the instrument's xenon lamp has warmed up for at least 30 minutes to ensure intensity stability. Verify slit widths (typically 5–10 nm) are identical for excitation and emission scans. Wider slits increase intensity but decrease resolution. Handle quartz cuvettes by the frosted sides only; fingerprints or scratches cause scattering artifacts.

c. ***Expected Results:***
   i. Excitation maximum (λ_{ex}): 348 ± 2 nm
   ii. Emission maximum (λ_{em}): 450 ± 2 nm
   iii. Emission intensity at 450 nm should be ≥ 10^4 counts at high sensitivity.
   iv. Excitation and UV–VIS spectra of quinine should exhibit similar band shapes, confirming correct instrument alignment.
   v. Quality Control Indicators:
   vi. Signal-to-noise ratio (S/N) > 50 at λ_{em} = 450 nm, indicates good detector sensitivity.
   vii. Spectral resolution: FWHM < 15 nm at slow scan speed.
   viii. Photometric linearity: Intensity doubles when concentration doubles within 0.5–5 ppm range.

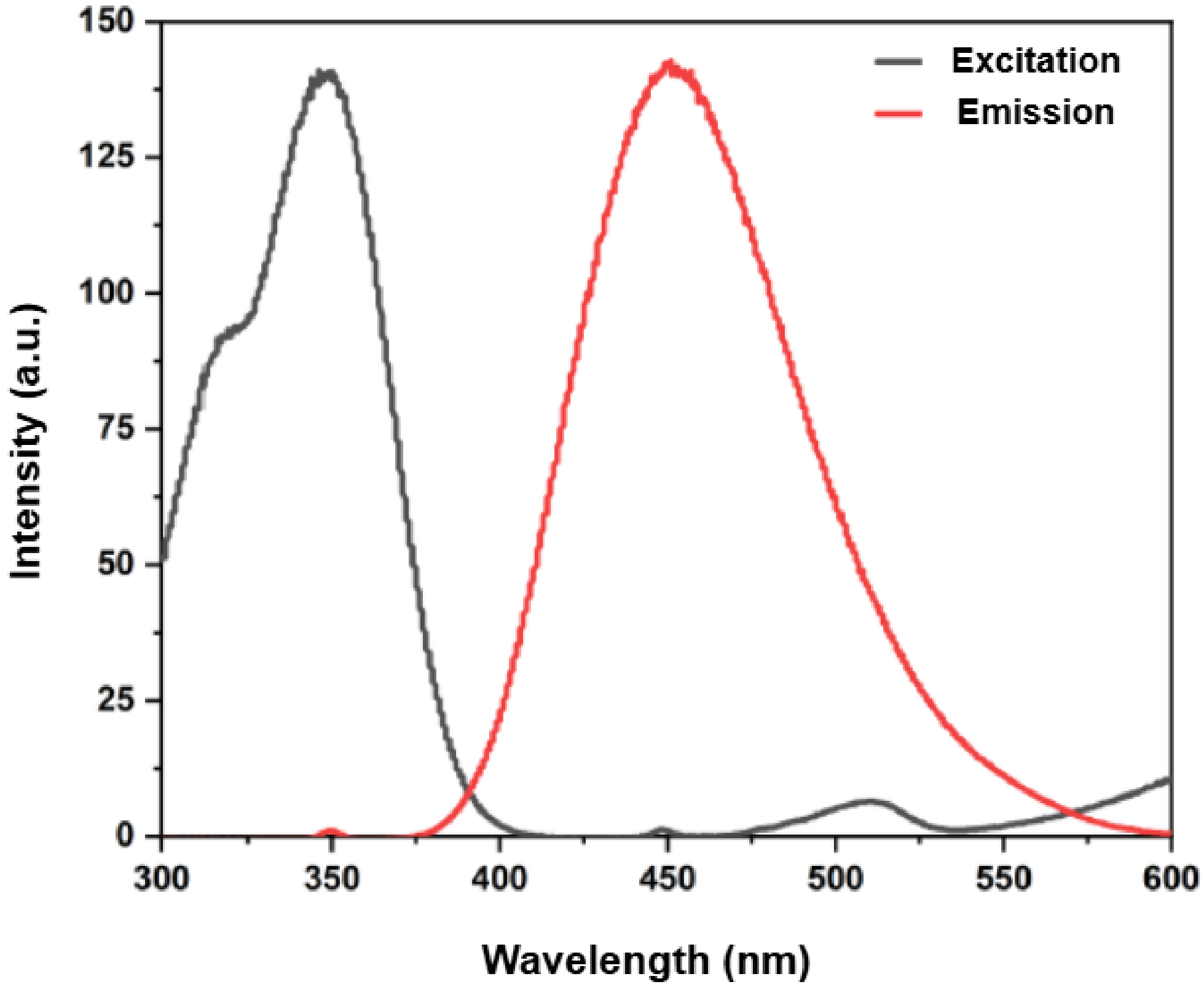

Figure 4. Example of the Expected Fluorescence Excitation and Emission Spectra for Quinine

d. **Common Issues & Troubleshooting:**
    i. Weak or unstable signal: Check lamp alignment or replace aged lamp. Shift in $\lambda_{ex}/\lambda_{em}$ > 3 nm: Indicates wavelength calibration drift verify using a holmium oxide filter.
    ii. High background: Residual acid in cuvettes rinse thoroughly with deionized water and methanol.
    iii. Interference fringes: Use a thinner film or re-seat the standar to remove air gaps.

e. ***Analysis of the Unknown:*** For this experiment, students used the same solution they prepared for UV-Vis (0.05 mg/mL). In the case of fluorescence spectroscopy (Shimadzu RF-5301pc Spectrofluorometer), the excitation and emission scans only showed instrument artifacts around ~ 450 nm; these peaks had the same intensity and overlapped with each other. This result was expected for this compound because ferrocene is a known luminescence quencher. [39-40] Students concluded that this chalcone did not fluoresce; however, they were able to appreciate this phenomenon with the quinine spectrum.

f. ***Pedagogical Emphasis***: Encourage students to discuss why quinine's excitation spectrum mirrors its UV–Vis absorption and how emission

intensity depends on molecular structure and environment (pH, solvent polarity). Highlight the concepts of inner-filter effect, quantum yield, and instrumental parameters.

6. FTIR (PerkinElmer Spectrum Two, UATR Two, Fourier Transform Infrared (FT-IR) Spectrophotometer

a. ***Calibration/Preparation:*** Calibration with a polystyrene film standard validates wavelength accuracy and spectral resolution of the FTIR instrument before sample analysis. Polystyrene is widely used because of its sharp, reproducible absorption bands across the mid-infrared region (4000–400 cm^{-1}). This check ensures students' spectra of ferrocenyl chalcones can be confidently interpreted for functional group identification and comparison to literature values.

b. ***Critical Parameters:*** Ensure the FTIR has been powered for ≥30 minutes to stabilize the source and interferometer. Use a clean ATR crystal (e.g., diamond or ZnSe) free of residue from previous samples. For transmission calibration, ensure the polystyrene film is flat, unwrinkled, and at room temperature to avoid shifts due to thermal expansion. Collect at least 16 scans at a resolution of 4 cm^{-1} over 4000–400 cm^{-1}. Always perform a background scan prior to calibration and after any environmental changes (humidity, temperature).

c. ***Expected Results:***
   i. Diagnostic polystyrene bands appear at 1601, 1583, 1154, 1028, 908, and 698 cm^{-1}.
   ii. The difference between measured and reference wavenumbers should be ≤2 cm^{-1}.
   iii. Signal-to-noise ratio should exceed 150:1 in the 2000-1000 cm^{-1} region.
   iv. The baseline should remain flat, with minimal water vapor or CO_2 interference.

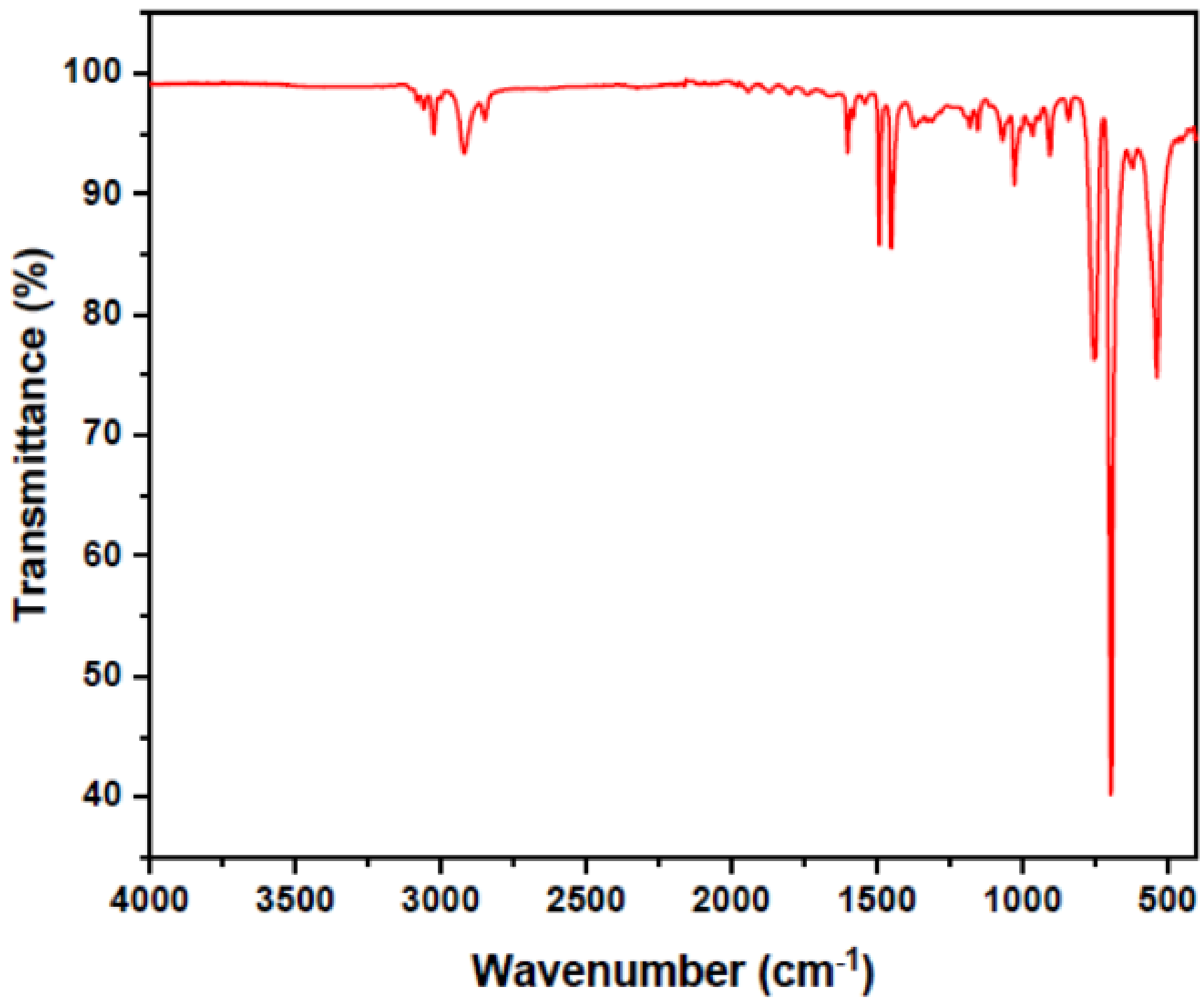

Figure 5: Expected FTIR Spectrum with Polystyrene Calibration Standard

d. ***Common Issues & Troubleshooting:***
   i. Broad or shifted peaks: Check the background or purge system; ambient water and CO_2 absorb strongly near 3700–3500 and 2350 cm^{-1}. Weak signal: Ensure proper contact pressure in ATR mode; clean the crystal and sample window.
   ii. Interference fringes: Use a thinner film or re-seat the standard to remove air gaps.

e. ***Analysis of the Unknown:*** Students used a solid sample directly from the unknown sample vials for this technique. Students ran a background before experiments by leaving the sample platform unloaded. For sample experiments, 16 scans were chosen in the software, and a range of 4000 - 400 cm^{-1} was selected. Around ~1-2 mg (enough sample to cover the ATR crystals) were loaded into the sample platform.

f. ***Pedagogical Emphasis:*** *Link* this calibration to Good Laboratory Practice (GLP) concepts: wavelength verification and baseline correction are QA/QC requirements. Encourage students to interpret how stretching and bending modes in the calibration standard relate to those in ferrocenyl chalcones (e.g., C=O stretch at ~1655 cm^{-1}, Fe–C stretch at ~480 cm^{-1}). Have students note that stable, accurate calibration is essential for reliable peak assignment and functional group identification.

7. ^{1}H-NMR (Spinsolve 80 benchtop NMR)

a. ***Calibration/Preparation:*** Calibration of the ^{1}H-NMR benchtop system ensures magnetic field homogeneity, frequency accuracy, and spectral resolution, which are essential for correctly interpreting the chemical environments of ferrocenyl chalcones. The Spinsolve 80 benchtop NMR system provides a compact, automated platform that introduces students to the fundamentals of nuclear magnetic resonance operation and spectral calibration in a safe and accessible format. The benchtop NMR used for this experiment employs a *Standby* mode in which the instrument magnets are constantly being homogenized by continuous shimming. A standard reference sample (D_2O:H_2O – 95:5) is always left inside the instrument bore while shimming in S*tandby* mode. For running experiments, the *Standby* mode is stopped, and a ~ 5-7 min. Shim is run to ensure instrument readiness. The instructor guides the students through the software interface and explains the steps that are being carried out.

b. ***Critical Parameters:***

   i. Maintain the instrument in Standby mode when not in use. During this period, the system continuously performs active shimming using a standard reference tube (D_2O:H_2O, 95:5), ensuring the magnetic field remains stable and homogenized.

   ii. Before analysis, stop Standby mode and perform a manual or automatic shim cycle (~5–7 min) to confirm magnetic field uniformity.

   iii. Check frequency calibration using the water resonance in the D_2O:H_2O standard; it should appear at 4.79 ppm ± 0.02 ppm.

   iv. Ensure probe cleanliness and dryness; any solvent residue or dust can cause spectral artifacts or broadened peaks.

   v. Confirm instrument temperature stability (ideally 20–25 °C) before inserting samples to prevent frequency drift.

c. ***Expected Results:***

   i. Resolution: Linewidth @ 50% (< 0.50 Hz), Linewidth @ 0.55% (< 20.0 Hz).

   ii. Spectral noise < 1.0×10^{-4} relative to the water peak.

   iii. Peak position reproducibility within ±0.01 ppm across repeated calibrations.

   iv. A stable lock and flat baseline indicate proper magnet shimming and phase correction.

d. ***Common Issues & Troubleshooting:***

   i. Poor shimming or distorted peaks: Re-run the shim routine or verify that no ferromagnetic material is near the magnet.

   ii. Residual solvent signals or contamination: Clean the sample holder; use only high-purity deuterated solvents.

iii. Peak shifts >0.05 ppm: Verify temperature stability and solvent composition; D_2O:H_2O ratio affects the lock frequency.
iv. Low signal-to-noise ratio: Increase the number of scans (up to 32) or check sample concentration (~10 mg/mL recommended).
v. Quality Control Indicators:
   a. Water reference at 4.79 ± 0.02 ppm.
   b. Signal-to-noise ratio (D_2O)> 50:1.
   c. Consistent peak integrals for repeated scans confirm field stability and probe sensitivity.
   d. FWHM ≤ 1 Hz at half-height for the standard peak ensures adequate spectral resolution.

e. ***Analysis of the Unknown***: Students weighed 7-10 mg of their unknown sample and diluted it in ~ 1 mL of deuterated chloroform $CDCl_3$ in a clean test tube. Then, the solution was transferred to an NMR sample tube (5 mm [o.d.]) and sealed with an NMR tube cap. Students used a delicate task wipe to clean the outside of their tubes before inserting it into the instrument. For this step, the instructor inserted the sample tube into the instrument bore. In the instrument software, 16 scans were selected for the proton NMR experiment, and preliminary processing was performed there.

f. ***Pedagogical Emphasis:*** Encourage students to connect field homogeneity (shimming) and frequency calibration to the concept of chemical shift reprodu-cibility. Demonstrate how the D_2O/H_2O standard serves as a reference for field stability and why deuterated solvents are essential for decoupling lock and sample signals. Highlight that proper NMR calibration mirrors professional QA/QC practices in analytical chemistry, ensuring the accuracy of structural assignments in ferrocenyl chalcones and other organic compounds.

8. DSC (METTLER TOLEDO Polymer DSC / 822e DSC) / TGA (METTLER TOLEDO TGA 2)

a. ***Calibration/Preparation:*** Differential Scanning Calorimetry (DSC) and Thermogravimetric Analysis (TGA) are complementary techniques that assess thermal purity, phase transitions, and decomposition behavior. Calibration of both Differential Scanning Calorimetry (DSC) and Thermogravimetric Analysis (TGA) using high-purity indium ensures accurate temperature, enthalpy, and mass measurements across both instruments. This dual calibration verifies thermal response, baseline stability, and heat-flow accuracy critical for evaluating purity, melting transitions, and decomposition behavior of ferrocenyl chalcones. An indium reference was used as a standard for calibration. The standard was transferred to an aluminum crucible. An aluminum lid was put on top, hermetically sealed with a crucible sealing press, and then pin holed to prevent pressurization and ensure consistent volatilization. The DSC/TGA

experiments were run for the standard, and results were compared to the theoretical value to determine error in measurements.

b. ***Critical Parameters:*** Use certified 99.99% indium metal as the calibration standard for both DSC and TGA instruments. Seal indium pieces (~5–10 mg) in clean aluminum crucibles with pin-holed lids to prevent pressurization and ensure consistent volatilization. Conduct calibration and all sample runs under nitrogen purge (60 mL/min) to avoid oxidation and maintain thermal equilibrium. Set the heating rate at 10–15 °C/min; temperature range: 25–350 °C. Balance calibration for TGA is automatically validated through the indium's stable mass profile, no weight loss should occur below 320 °C. Reference crucible should match the sample crucible in geometry and sealing to ensure accurate thermal balance.

c. ***Expected Results:***
   i. Indium melting point (T_m): 156.6 ± 0.3 °C
   ii. Heat of fusion (ΔH_{fus}): 28.45 J/g ± 1.0 J/g
   iii. DSC: Sharp, single endothermic peak with flat baseline before and after melting.
   iv. TGA: Constant mass until indium melting (~156 °C), followed by <0.1% total weight loss (verifying system stability).
   v. Baseline stability: ≤ ±0.01 mg drift and ≤ ±0.5 °C temperature deviation across replicate runs.

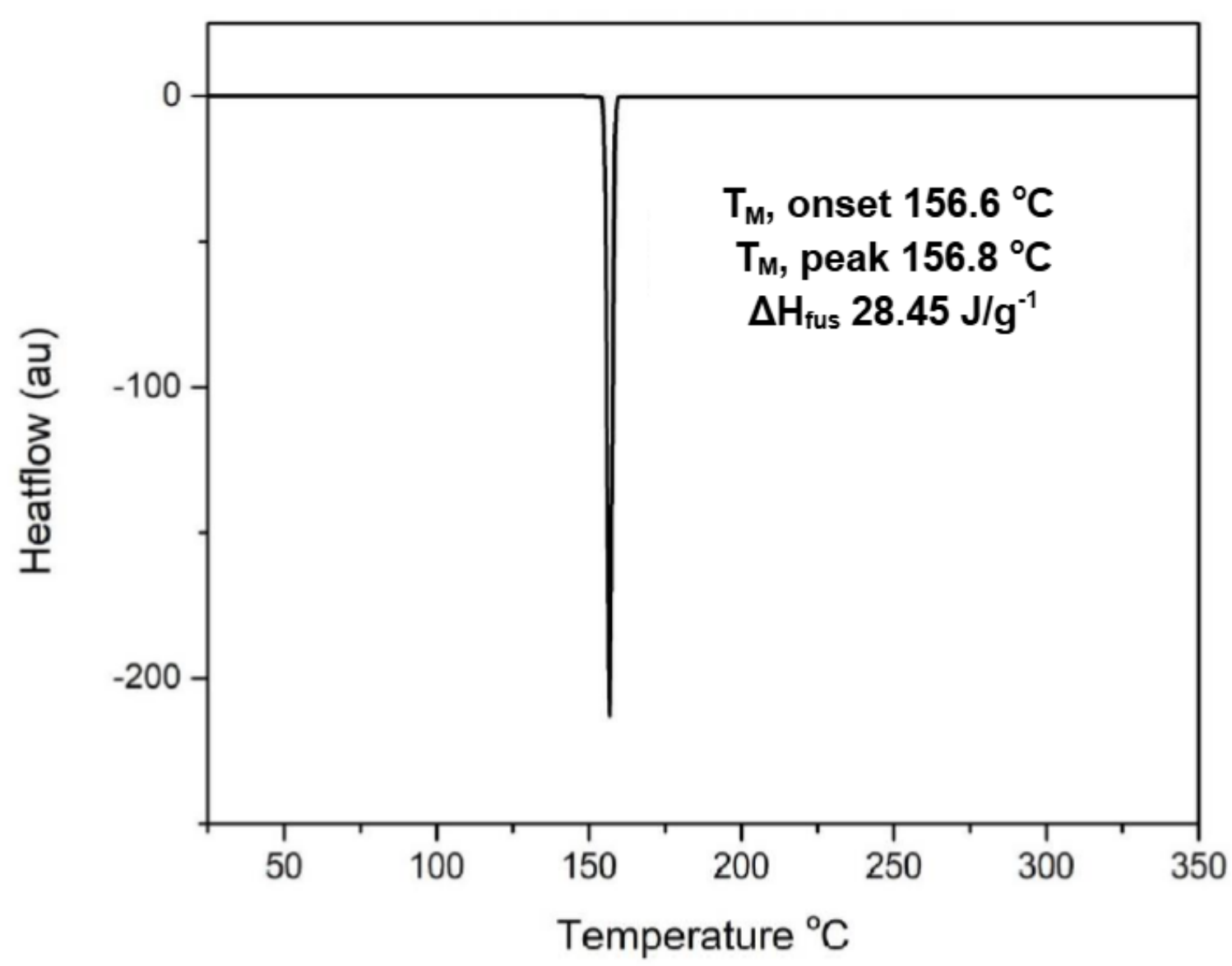

Figure 6. DSC Calibration Thermogram Example (Indium Standard)

d. ***Common Issues & Troubleshooting:***

i. Shifted melting peak or broadened transition: Verify temperature calibration and crucible sealing; contaminants or moisture can cause thermal lag.
ii. Mass drift in TGA: Indicates purge gas flow instability or buoyancy effects; re-zero balance and confirms nitrogen flow.
iii. Excessive baseline curvature: May result from residual grease or sample residue on the sensor platform—clean with acetone and run a blank heating cycle.
iv. Asynchronous DSC/TGA peaks: Ensure both instruments share identical temperature calibration parameters and ramp rates.
v. Quality Control Indicators:
    a. T_m deviation ≤ 0.5 °C; ΔH deviation ≤ 1.0 J/g.
    b. Baseline reproducibility within ±0.01 mg and temperature reproducibility ≤ 2 °C. Consistent melting point across consecutive runs confirms both thermal accuracy and mass stability.

e. ***Analysis of the Unknown***: Students weighed ~2-5 mg of their unknown sample and transferred it into an aluminum crucible. An aluminum lid was put on top, hermetically sealed with a crucible sealing press, and then pin holed to prevent pressurization and ensure consistent volatilization. The exact weight was determined by weighting by difference using the TGA internal balance. The instrument was set up to a heating rate of 15 °C/min and a temperature sweep from 25 °C to 350 °C under nitrogen purge (60 mL/min). The instructor guided students to insert the sample into the instrument using tweezers. For ferrocenyl chalcones: melting transitions typically 150–200 °C; T_{onset} (decomposition) between 250–280 °C. Thermal data was evaluated in METTLER-TOLEDO STARe, for DSC purity, melting temperatures and TGA metrics. The procedure followed established reference methods for calibration and data interpretation, including METTLER TOLEDO Thermal Analysis Application No. 101 – DSC Purity Determination (UserCom 10, 2010) and ASTM International Standards E928-19 (“Standard Test Method for Determination of Purity by Differential Scanning Calorimetry”) and E794-06R18 (“Standard Test Method for Melting and Crystallization Temperatures by Thermal Analysis”). These references provided the methodological framework for purity determination, thermal transition analysis, and temperature calibration under controlled inert conditions.

f. ***Pedagogical Emphasis***: Guide students to understand that indium serves as a thermal reference material for both DSC and TGA calibration because it provides a reproducible melting event without decomposition or mass change. Emphasize how DSC quantifies energy transitions (endothermic/exothermic) while TGA measures mass changes, and that proper calibration establishes the analytical reliability of all subsequent thermograms. Encourage students to compare DSC and TGA traces for

indium to see how enthalpy and mass stability align, reinforcing concepts of instrument validation, QA/QC, and thermal behavior correlation in material characterization.

C. Waste Disposal

All the generated waste was disposed of to the appropriate recipients, depending on composition. *Spent halogenated solvents* include the deuterated solvent used for NMR samples; *spent non halogenated solvents* include the solutions prepared for HPLC, UV-Vis, Fluorescence, and TLC mobile phase; *solid laboratory waste* includes TLC plates, capillary tubes, aluminum crucibles, Pasteur pipettes, and solid ferrocenyl chalcones samples.

D. Example Technical Sheet

The following pages include an example of a filled theoretical technical sheet and a filled experimental sheet with data obtained from a group of students. A complete overview of obtained results can be found in the main text.

Technical Sheet: Ferrocenyl Chalcone

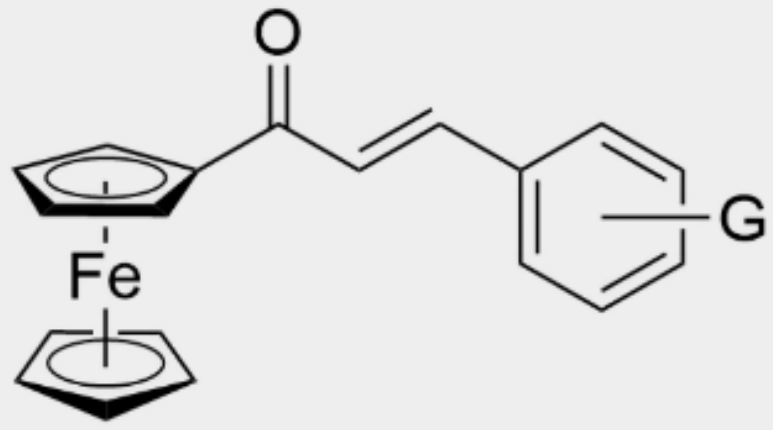

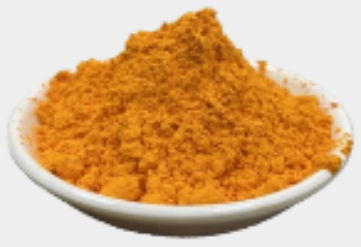

—

Characterization and Quality Control of Ferrocenyl Chalcone Derivatives Utilizing Instrumental Techniques

Name: ______________________ Assigned Sample: **Theoretical**

Sample Identity:

Information	Structure
IUPAC Name: **3-(4-Bromophenyl)-1-ferrocenyl-2-propen-1-one (p-Br)**<br>Molecular Formula: **$C_{19}H_{15}OBrFe$**<br>Molecular weight (g/mol): **395.07 g/mol**<br>Observed Color: **reddish orange**<br>Physical State: **crystalline solid**<br>Relative Purity: **high**<br>Thermal Stability: **stable**	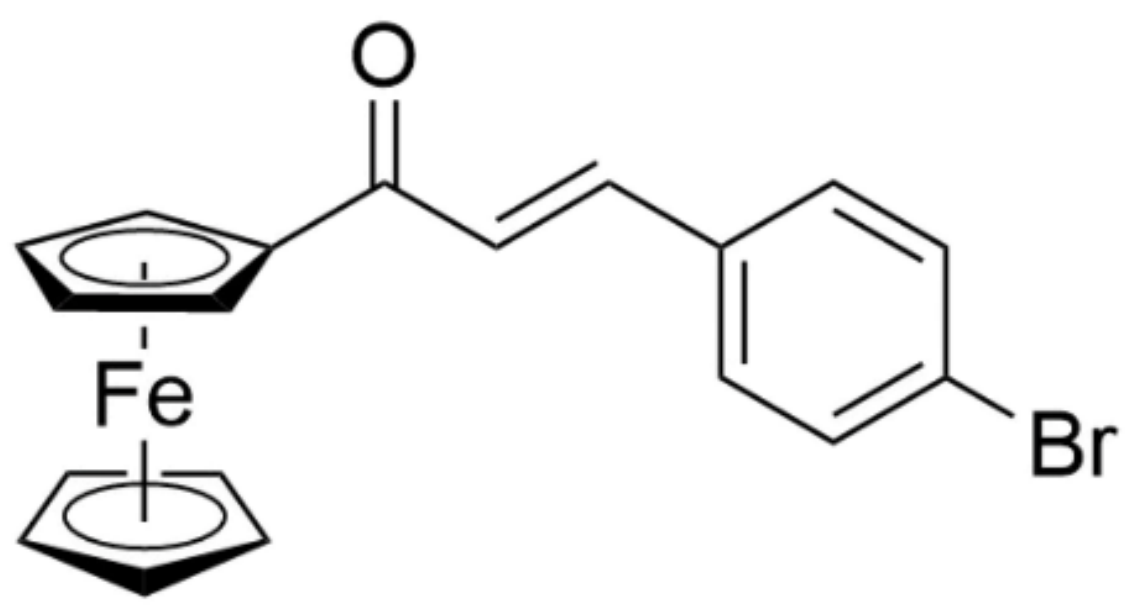

Instrumental Characterization:

HPLC
Purity: **> 95% (Standard)**

UV-Vis
λ_{max}: **257 nm**

FTIR
Characteristic Peaks:

Carbonyl: 1650-1660 cm^{-1},
Fe-C: 480-500 cm^{-1}
Aromatic: 1600-1475 cm^{-1}
Aromatic para-Position:
2000-1667 cm^{-1}, 800 cm^{-1}

H-NMR
Characteristic Peaks:

Ferrocene (3 peaks): 4-5 ppm
Vinyl (2 peaks): 5-6 ppm
Aryl (2 peaks): 7-8 ppm

DSC
T_m: **165 °C**

TGA
Onset: **> 250 °C**

Observations:

____

____

____

____

____

Technical Sheet: Ferrocenyl Chalcone

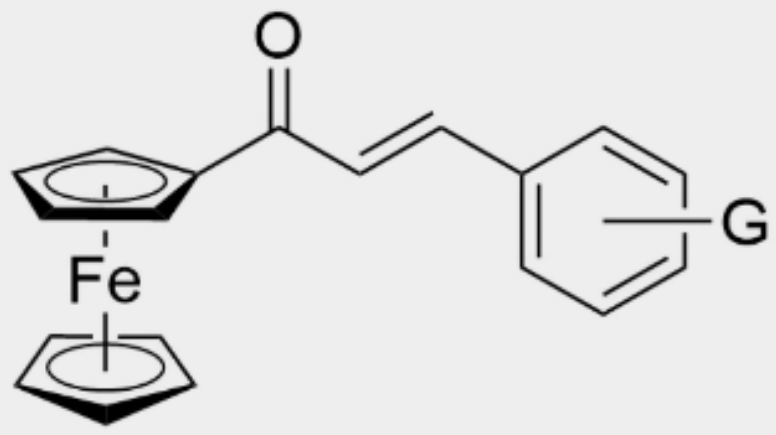

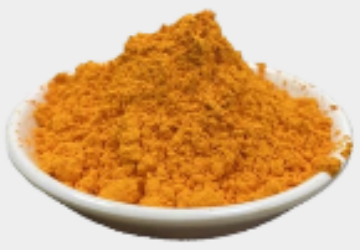

Characterization and Quality Control of Ferrocenyl Chalcone Derivatives Utilizing Instrumental Techniques

Name: ______________________________ Assigned Sample: **Experimental (Students)**

Sample Identity:

Information	Structure
IUPAC Name: **3-(4-Bromophenyl)-1-ferrocenyl-2-propen-1-one (p-Br)**<br>Molecular Formula: **$C_{19}H_{15}OBrFe$**<br>Molecular weight (g/mol): **395.07 g/mol**<br>Observed Color: **reddish orange**<br>Physical State: **crystalline solid**<br>Relative Purity: **high**<br>Thermal Stability: **stable**	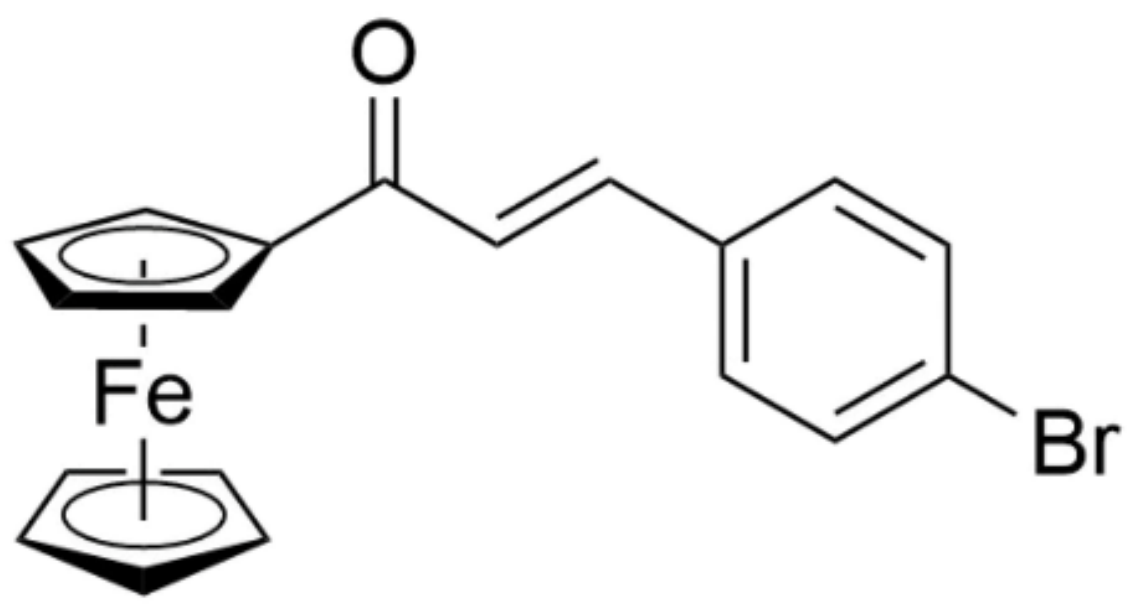

Instrumental Characterization:

HPLC
T_r: **13.197 min** Purity: **95.81 %**

UV-Vis
λ_{max}: **226.50 nm**

FTIR
Characteristic Peaks:

Carbonyl: 1650.46 cm^{-1},
Fe-C: 478.55 cm^{-1}
Aromatic: 1560.47 cm^{-1}
Aromatic para-Position:
1594.80 cm^{-1}, 813.36 cm^{-1}

H-NMR
Characteristic Peaks:

Ferrocene: 4.63, 4.33, and 3.94 ppm
Vinyl and Aryl: 6.71, 7.26, 6.99, and 7.55 ppm

DSC
T_m: **180.5 °C**

TGA
Onset: **200 °C**

Observations:

Key results confirming the identity include NMR shifts and infrared bands. The compound did not show any impurities or deviations. According to the AI predictions, the most probable compound is 3-(4-methoxyphenyl)-1-ferrocenyl-2-propen-1-one, because bromine does not favor fluorescence as much. At the same time, the methoxy group is a strong electron donor and would enhance fluorescence.

Implications for Other Institutions / How to Adopt this Model

The article presents a comprehensive model for organic synthesis and characterization of ferrocenyl chalcones; however, its design allows for adaptations where the core spiral curricular experience can be met. One of the most important keys to success is the coordination between professors from Organic Chemistry and Instrumental Analysis courses in view of a collaborative effort. Within this coordination, details such as materials needed, available instrumentation, time slots for experiments, among others can be arranged. The implementation and adaptation can be fine-tuned based on the institution's resources and schedules. Below are a set of suggestions that allow for modifications that still meet the spiral curriculum and the main synthetic and unknown identity determination.

- Ferrocenyl chalcones as synthetic products:
    - The presented ferrocenyl chalcones are good academic target molecules due to their feasibility of product obtention and purification.
    - Alternatives for Starting Materials:
        - Narrowing down the set of substituents based on the available inventory
        - Choosing one substituent and work with multiple substitution or positions in the aromatic ring
        - Alternating G groups between academic semesters
        - Time limitations can be addressed based on the reaction time for different G groups
        - Synthesis of regular chalcones (Ar-CO-C=C-Ar'; Ar’-CO-C=C-Ar) can also be explored
        - Synthetic products that are already part of the established organic chemistry laboratory experiments and that can be characterized with the presented techniques can also be used as a modification to the presented approach
- Characterization Techniques
    - The used ferrocenyl chalcones are well behaved when analyzed with the presented characterization techniques
    - Alternatives for Characterization Techniques:
        - Unknown sample identity – even though the proposed methodology includes multiple characterization techniques, this can be modified based on the institutions’ available resources. In essence, there are some techniques that are used to understand chemical processes occurring with the samples but that not necessarily provide a definitive tool for identity (e.g., UV-Vis)
            - TLC – this technique and required materials are usually part of the organic chemistry laboratory and can give a general idea of the contents of a sample and some impurities.
            - Melting Point – this technique and required materials are usually part of the organic chemistry laboratory. The melting

point of the presented ferrocenyl chalcones varies depending on the substituent and on the position of a substituent. This can be used as a tool for comparison with published values to help identify unknown samples.

- HPLC – Institutions that do not have an instrument available can use techniques such as TLC or NMR to determine sample purity.
- UV-Vis – This technique is used as part of the Instrumental Analysis laboratory course. Because the spectra between samples is similar, the implementation has a more pedagogical purpose as a physicochemical process of interaction with light. In addition, the methodology asks students to provide a detailed characterization profile for their sample.
- FLU – This technique is used as part of the Instrumental Analysis laboratory course. Because the spectra between samples is similar, the implementation has a more pedagogical purpose as a physicochemical process of interaction with light. In addition, the methodology asks students to provide a detailed characterization profile for their sample.
- FTIR – Institutions that do not have an instrument available can ask students to use an online spectrum simulator to produce a graph based on the students' conclusion of sample identity.
- NMR – Institutions that do not have an instrument available can ask students to use an online spectrum simulator to produce a graph based on the students' conclusion of sample identity.
- DSC – Institutions that do not have an instrument available can ask students to draw a simulated graph of expected results.
- TGA – Institutions that do not have an instrument available can ask students to draw a simulated graph of expected results.
- Institutions can use instruments available to them and include them as part of the characterization sequence. For example, Mass Spectrometry, Elemental Analysis, X-Ray Diffraction, paper chromatography, among others.

- Group Sizes:
    - The student groups can be adjusted based on the laboratory group size. However, groups that are too big (i.e., n > 4) can affect student learning negatively as each student will only perform small tasks.
- Laboratory Sessions:
    - The proposed methodology implements the laboratory experience within 3 laboratory sessions based on our institutions' curriculum for the laboratory courses.
    - Alternatives for Number of Laboratory Sessions:
        - Institutions can adapt the proposed methodology based on their number and time spent in each laboratory session. Within this context, a selection of all characterization techniques can be made, groups can be made smaller/bigger depending on available time, and some tasks can be assigned as homework, to mention a few modifications.
- In-Class Sessions
    - The proposed methodology makes use of the Instrumental Analysis course periods as an introduction to the proposed methodology, including the AI-assisted predictions.
    - Alternatives for In-Class Sessions:
        - Professors can choose to use in-class sessions or to use only laboratory sessions for this experience. In the latter case, the time spent in each laboratory session as well as the selection of experiments to be carried out need to be previously coordinated.

SLF_SupportingInformation_4_StudentManual

Questionnaire

Laboratory Manual Questionnaire: Characterization and Quality Control of Ferrocenyl Chalcone Derivatives Utilizing Instrumental Techniques

STATION I: HIGH PERFORMANCE LIQUID CHROMATOGRAPHY (HPLC/UV-Vis) – Caffeine Calibration, TLC Prediction and Ferrocenyl Chalcone Purity Assessment

Questions:

1. What does the R^2 value indicate about the performance of HPLC?
2. How many components did TLC predict?
3. Do TLC and HPLC results agree in peak number and order? What advantages/disadvantages does TLC have compared to HPLC?
4. Is the sample pure? How many impurities exist?
5. What could be the sources of error in this experiment?
6. How can you determine the identity of impurity using TLC and using HPLC?

STATION II: UV-Vis SPECTROSCOPY – Stability Verification and UV-Vis Characterization

Questions:

7. What are the concentrations (in M) of the solutions A and B of potassium dichromate according to your results? How does the spectrum of both solutions compare to each other? What solution would be more appropriate for a precise calibration of the instrument?
8. What is the wavelength of maximum absorption (λ_{max}) of the unknown ferrocenyl chalcone? How does the shape of the obtained spectrum compare with reference spectra of similar ferrocene derivatives? Based on this comparison, is the analyzed sample pure?
9. What type of information can be obtained from the UV-Vis spectrum of the unknown ferrocenyl chalcone? What are the limitations of this technique for its characterization?
10. What precautions must be taken when preparing solutions and taking measures to ensure the precision and accuracy of the results?
11. How can UV-Vis be used in combination with other analytical techniques, such as chromatography, to obtain a more comprehensive characterization of the unknown ferrocenyl chalcone?

STATION III. FLUORESCENCE - Fluorescence Characterization

Questions:

12. How does a faster scan velocity affect the resolution of the emission spectrum?
13. Why is it important to choose the adjustment of sensitivity appropriate for the fluorescence measurements?
14. Compare and contrast the processes of absorption, excitation, and emission, illustrated with a Jablonski diagram.

STATION IV: FTIR SPECTROPHOTOMETRY – Identification of Functional Groups and Structural Confirmation

Questions:

15. According to the results obtained with the polystyrene, is the FTIR Spectrophotometer calibrated correctly? Justify your answer.
16. Based on the FTIR spectrum, what functional groups can you identify in the unknown ferrocene derivative? Are these functional groups consistent with the structure of the compound?
17. Are there peaks in the FTIR that do NOT correspond to the expected structure? If there are, what could these peaks indicate?
18. How can you use the information obtained with FTIR in combination with the results of other analytical techniques (HPLC or NMR) to confirm the structure of an unknown ferrocenyl chalcone?
19. What are the limitations of FTIR Spectroscopy for characterizing organic compounds?

STATION V: NUCLEAR MAGNETIC RESONANCE (NMR) – Structural Confirmation and Purity Assessment

Questions:

20. What is the molecular formula and molecular weight of the unknown ferrocene derivative? What information did you use to determine these values?
21. In the ^{1}H-NMR spectrum, which signals correspond to the hydrogen atoms in the cyclopentadienyl ring? How do you justify the assignment based on chemical displacement and the signal multiplicity? How does the chemical shifts of these aromatic hydrogens of the cyclopentadienyl ring compare with other aromatic hydrogens? Explain the difference in displacement (Review the concept of anisotropy).
22. What substituents are present in the unknown ferrocenyl chalcone as determined by the ^{1}H-NMR? What is the position of the substituent in the

aromatic ring? How do you justify the assignment of peaks based on the chemical shifts, splitting, and signal coupling patterns?

23. According to the NMR spectrum, what is the purity of the unknown ferrocene derivative? What are the possible impurities you have identified in the sample?
24. What are the limitations of NMR for characterizing organic compounds? What type of additional information could be obtained with other spectroscopy or chromatography techniques?

STATIONS VI and VII Thermal Analysis – Differential Scanning Calorimetry (DSC) and Thermogravimetric Analysis (TGA)

Questions:

25. What does the melting temperature obtained in DSC analysis of the unknown indicate? Compare the value with the values in the literature.
26. Does the form of the melting peak in DSC suggest that the ferrocene is pure or impure? Explain.
27. If there were more than one peak in the DSC curve, explain what would be the effect on the melting point when a soluble impurity is present in the sample analyzed? What would be the effect if the impurity were insoluble?
28. At what temperature does the ferrocenyl chalcone start to degrade, as determined by the TGA analysis? Is this temperature reasonable, considering the structure of the compound?
29. What information about the decomposition of the ferrocenyl chalcone can be obtained from the TGA curve?
30. How do the results in DSC and TGA compare to each other? Do they complement each other to have a more complete characterization?
31. What are the possible errors of this experiment? How can they optimize the precision of the measurements?

- **Upload all tables from Results and Analysis into one document. (Please label each station and add the table title)**

SLF_SupportingInformation_5_PrelaboratoryEvaluation

Rubric For Evaluation of the Pre-Laboratory Experience

Purpose: To assess students' ability to predict, justify, and plan analytical characterization of ferrocenyl chalcones across multiple techniques (HPLC, FTIR, NMR, UV–Vis, DSC/TGA) while integrating AI-based reasoning, QA/QC, and scientific reflection.

Criteria	Excellent (4)	Proficient (3)	Developing (2)	Beginning (1)
Identification of Possible Impurities	Clearly identifies all relevant sources (unreacted starting materials, by-products, decomposition products) with specific examples and chemical reasoning.	Identifies most impurity types with partial explanation.	Mentions general impurities without clear chemical reasoning.	Provides incomplete or incorrect list of impurities.
Instrumental Manifestation of Impurities (HPLC, FTIR, NMR, DSC/TGA)	Accurately predicts characteristic spectral/chromatographic changes for each instrument, linking them to impurity type.	Predicts most instrumental responses with reasonable accuracy.	Provides limited or vague predictions without clear connection to impurity identity.	Inaccurate or missing predictions for most techniques.
Predictive Analysis (UV–Vis Substituent Effects)	Provides correct and well-justified explanation of λ_{max} shifts using molecular orbital theory and inductive/mesomeric effects.	Provides mostly correct prediction with basic justification.	Prediction given with minimal explanation or conceptual errors.	Incorrect or missing analysis of substituent effects.
Integrated Instrumental Predictions Table	Completes the table thoroughly with consistent, realistic values and clear trends across all instruments.	Provides most predicted values; minor inconsistencies or missing data.	Incomplete table or inconsistent values; lacks rationale.	Minimal or incorrect entries; lacks understanding of relationships.
AI-Assisted Prediction and Reflection	Demonstrates critical comparison between AI and human predictions; identifies biases, limitations, and data-driven corrections.	Compares AI and human results with moderate reflection on discrepancies.	Summarizes AI output with limited critical analysis.	Relies solely on AI output without evaluation or justification.
QA/QC Plan Design	Proposes comprehensive, realistic calibration and control plan with appropriate standards, acceptance criteria, and verification steps.	Includes key QA/QC elements but lacks one or two important components.	Basic plan with general statements; lacks measurable acceptance criteria.	Incomplete or unrealistic QA/QC plan.
Pre-registration of Identity Hypothesis	Develops logical, data-based hypotheses with clear decision criteria and decisive technique justification.	Provides reasonable hypotheses with limited rationale.	Hypotheses vague or missing decisive technique justification.	No clear hypothesis or justification.

Criteria	Excellent (4)	Proficient (3)	Developing (2)	Beginning (1)
Conceptual Understanding (Mini Prelab Quiz)	Accurately answers all conceptual questions with clear, scientifically sound reasoning.	Minor inaccuracies; overall demonstrates understanding.	Some correct concepts but with notable misconceptions.	Major misconceptions or incomplete answers.
Scientific Communication and Clarity	Responses are precise, well-organized, and use appropriate scientific terminology.	Generally clear and well-structured with minor lapses in terminology.	Disorganized or lacks technical precision.	Incoherent, minimal, or off-topic responses.

Scoring and Weighting (Total = 100%)

Category	Weight
Identification & Instrumental Prediction (Criteria 1–2)	25%
Predictive Analysis & Table (Criteria 3–4)	20%
AI Integration & Reflection (Criterion 5)	15%
QA/QC Plan & Hypothesis (Criteria 6–7)	20%
Conceptual Quiz & Communication (Criteria 8–9)	20%

Performance Interpretation:
- 90–100 (Excellent): Mastery of predictive reasoning and interdisciplinary integration.
- 75–89 (Proficient): Solid conceptual understanding and methodological awareness.
- 60–74 (Developing): Partial understanding; reinforcement needed.
- <60 (Beginning): Lacks conceptual and procedural understanding.

SLF_SupportingInformation_6_ComprehensiveImpact Evaluation

Ferrocenyl Chalcones Experiment: Comprehensive Impact Evaluation Questionnaire

Introduction:

This questionnaire aims to gather in-depth feedback on the ferrocenyl chalcones experiment, focusing on its impact on your attitudes toward materials chemistry, your views on laboratory work, and your understanding of new concepts. Additionally, we seek your insights into how this activity can help shape the future of materials and design in education. Your thoughtful and honest responses are invaluable. Your responses will be used confidentially and anonymously to improve courses and generate data for educational research in materials, design thinking, sustainability, and the use of artificial intelligence in chemistry education.

Section 1 – General Information

- Name optional (Short answer)
- Age (Short answer)
- Gender (optional) (Dropdown list)
- Year of Study (Sophomore, Junior, Senior, Graduate) (Dropdown list)
- Chemistry Courses Completed Before This Lab (Checkboxes: Organic I, Organic II, Analytical, Instrumental, Other)

I. Attitudes Toward Learning Materials Chemistry

1. **Initial Interest:** Before this experiment, how interested were you in learning about materials chemistry? (Likert Scale: 1 - Not at all Interested, 5 - Extremely Interested)
2. **Post-Experiment Interest:** After completing this experiment, how interested are you in learning about materials chemistry? (Likert Scale: 1 - Not at all Interested, 5 - Extremely Interested)
3. **Shift in Perspective:** Did this experiment change your perspective on materials chemistry? (Yes/No) **If yes, please explain how your perspective changed and what aspects of the experiment contributed to this change.**
4. **Relevance Perception:** To what extent do you agree with the following statements:
    - Materials chemistry is relevant to real-world problems. (Likert Scale: 1 - Strongly Disagree, 5 - Strongly Agree)

- Materials chemistry is an exciting and innovative field. (Likert Scale: 1 - Strongly Disagree, 5 - Strongly Agree)
- I am more likely to explore advanced topics in materials chemistry because of this experiment. (Likert Scale: 1 - Strongly Disagree, 5 - Strongly Agree)

II. Views on Laboratory Work

1. **Enjoyment of Lab Work:** How much did you enjoy the laboratory work component of this experiment? (Likert Scale: 1 - Not at all Enjoyable, 5 - Extremely Enjoyable)
2. **Perceived Value of Lab Experience:** How valuable do you think this laboratory experience was for enhancing your understanding of materials chemistry? (Likert Scale: 1 - Not at all Valuable, 5 - Extremely Valuable)
3. **Skill Development:** To what extent do you agree with the following statements:
   - This experiment improved my experimental skills. (Likert Scale: 1 - Strongly Disagree, 5 - Strongly Agree)
   - This experiment enhanced my data analysis skills. (Likert Scale: 1 - Strongly Disagree, 5 - Strongly Agree)
   - This experiment strengthened my problem-solving abilities. (Likert Scale: 1 - Strongly Disagree, 5 - Strongly Agree)

Section 2 – Critical Thinking

- I was able to identify assumptions and limitations of each instrumental technique. (Likert Scale: 1-5)
- I integrated data from different techniques to reach a coherent conclusion. (Likert Scale: 1-5)
- I identified discrepancies in the results and proposed reasoned explanations. (Likert Scale: 1-5)
- I was able to justify my conclusions with experimental evidence. (Likert Scale: 1-5)

Section 3 – Design Thinking

- I clearly understood the initial challenge of characterizing an unknown sample. (Likert Scale: 1-5)

- I generated diverse strategies or routes to solve the problem. (Likert Scale: 1-5)
- I adjusted my approaches in response to new experimental evidence. (Likert Scale: 1-5)
- I worked effectively with my team in an iterative problem-solving process. (Likert Scale: 1-5)
- My technical report reflects a design process rather than just a result. (Likert Scale: 1-5)

Section 4 – Instrumental Techniques Learning

- I understand the operating principles of HPLC, UV-Vis, FTIR, NMR, DSC, and TGA. (Likert Scale: 1-5)
- I can explain what type of information each technique provides for characterizing a material. (Likert Scale: 1-5)
- I recognize the importance of calibration and QA/QC in the validity of data. (Likert Scale: 1-5)
- I feel more confident in my ability to use instrumental equipment in future projects. (Likert Scale: 1-5)

Section 5 – Connection with Organic Chemistry and Materials Science

- I identified how the instrumental data confirms structures proposed in organic synthesis. (Likert Scale: 1-5)
- I recognize the relationship between aromatic substituents and the spectra obtained. (Likert Scale: 1-5)
- I understood how spectroscopic and thermal properties reflect the chemical structure. (Likert Scale: 1-5)
- The lab helped me to link concepts from Organic Chemistry with materials design. (Likert Scale: 1-5)

Section 6 – Attitudes Towards Sustainability and AI

- I recognize the importance of sustainability in materials research and design. (Likert Scale: 1-5)
- This experience allowed me to reflect on principles of circular economy and responsible materials selection. (Likert Scale: 1-5)
- I consider it useful to integrate AI tools to analyze experimental data or improve lab experience. (Likert Scale: 1-5)

- I am interested in learning more about the role of AI in materials chemistry and design. (Likert Scale: 1-5)

Section 7 – Global Impact

- The experience integrated theory and practice in a meaningful way. (Likert Scale: 1-5)
- It increased my motivation to learn about materials and their applications. (Likert Scale: 1-5)
- It improved my confidence in solving open-ended problems in STEM. (Likert Scale: 1-5)
- I believe that this lab developed transferable skills for research and industry. (Likert Scale: 1-5)

4. **Aspects of Lab Experience: What specific aspects of the laboratory work (e.g., techniques used, instruments operated, data analysis performed) did you find most beneficial and why?**
5. **Challenges Encountered:** What were some of the biggest challenges you encountered during the laboratory work and how did you overcome them?
6. Which instrumental technique was most decisive in identifying your sample? Explain why.
7. What limitations did you find in any technique and how did you complement it with another to overcome those limitations?

III. Understanding of New Concepts

1. **Conceptual Understanding:** How well do you understand the following concepts after completing this experiment?
    - Structure-property relationships (Likert Scale: 1 - Not at all Well, 5 - Extremely Well)
    - Spectroscopic techniques for materials characterization (Likert Scale: 1 - Not at all Well, 5 - Extremely Well)
    - Factors influencing materials properties (Likert Scale: 1 - Not at all Well, 5 - Extremely Well)
2. **Theoretical Knowledge Reinforcement:** Did this experiment reinforce your understanding of the theoretical background of materials chemistry? (Yes/No) If yes, please provide examples of how theoretical concepts came to life through the experiment.

3. **Application of Concepts:** How well do you feel you can apply the concepts learned in this experiment to new and unfamiliar situations? (Likert Scale: 1 - Not at all Well, 5 - Extremely Well)

4. **Concept Clarification:** What concepts, if any, remain unclear and what could be done to improve your understanding?

IV. Shaping the Future of Materials and Design for Education

1. **Inspiration for Innovation:** Did this experiment inspire you to think about new ways in which materials can be designed and used for real-world applications? (Yes/No) If yes, please describe any innovative ideas or potential applications that came to mind**.**

2. **Relevance to Future Careers:** How relevant do you think this type of experimental activity is for preparing students for future careers in materials science and engineering? (Likert Scale: 1 - Not at all Relevant, 5 - Extremely Relevant)

3. **Educational Design Implications:** How could similar experiments be designed to be more engaging and effective for educating future generations of materials scientists and engineers?

4. **Skills for Innovation:** Which specific skills developed through this experiment (e.g., critical thinking, problem-solving, experimental design) do you think will be most valuable for contributing to future innovation in materials science and design?

5. **Educational Enhancements:** What changes or enhancements to this experiment could make it even more effective in shaping the future of materials and design for education?

V. Synthesis (Prior Knowledge Application)

1. **Influence of Prior Knowledge:** How much did your previous synthesis experience (learned in organic chemistry) influence your understanding of the properties and characterization? (Likert Scale: 1 - Not at all, 5 - Very Much)

2. **Specific examples:** What are the *specific* ways your prior synthesis influenced you?

VI. Overall Evaluation/ Section 8 – Open-Ended Questions (Long Answer)

1. **Most Valuable Aspect:** What was the most valuable aspect of this experiment overall?

2. **Least Valuable Aspect:** What was the least valuable aspect of this experiment overall?
3. **Recommendations:** What are your *top three* recommendations for improving this experiment to maximize its educational impact?
4. What similarities or differences did you notice between Organic Chemistry theory and the experimental results in relation to the properties of the material?
5. Select a ferrocenyl chalcone with a specific substituent and explain how at least three instrumental techniques would confirm its identity and purity. Indicate what signals, peaks, or transitions you would observe and how they relate to Organic and Materials theory. **Be specific about the expected results of each technique.**
6. How would you apply what you learned in this lab to a real-world problem in industry, sustainability, or the design of new materials? **Describe a specific problem and your approach based on this lab.**
7. If you had access to AI tools, how would you use them to improve the analysis or interpretation of the results obtained in this lab? **Provide concrete examples of AI applications.**

SLF_SupportingInformation _7_EvaluationRubrics

Rubric For Evaluation of the Pre-Laboratory Experience

Purpose: To assess students' ability to predict, justify, and plan analytical characterization of ferrocenyl chalcones across multiple techniques (HPLC, FTIR, NMR, UV–Vis, DSC/TGA) while integrating AI-based reasoning, QA/QC, and scientific reflection.

Criteria	Excellent (4)	Proficient (3)	Developing (2)	Beginning (1)	Grade	Feedback
Identification of Possible Impurities	Clearly identifies all relevant sources (unreacted starting materials, by-products, decomposition products) with specific examples and chemical reasoning.	Identifies most impurity types with partial explanation.	Mentions general impurities without clear chemical reasoning.	Provides incomplete or incorrect list of impurities.		
Instrumental Manifestation of Impurities (HPLC, FTIR, NMR, DSC/TGA)	Accurately predicts characteristic spectral/chromatographic changes for each instrument, linking them to impurity type.	Predicts most instrumental responses with reasonable accuracy.	Provides limited or vague predictions without clear connection to impurity identity.	Inaccurate or missing predictions for most techniques.		
Predictive Analysis (UV–Vis Substituent Effects)	Provides correct and well-justified explanation of λmax shifts using molecular orbital theory and inductive/mesomeric effects.	Provides mostly correct prediction with basic justification.	Prediction given with minimal explanation or conceptual errors.	Incorrect or missing analysis of substituent effects.		
Integrated Instrumental Predictions Table	Completes the table thoroughly with consistent, realistic values and clear trends across all instruments.	Provides most predicted values; minor inconsistencies or missing data.	Incomplete table or inconsistent values; lacks rationale.	Minimal or incorrect entries; lacks understanding of relationships.		
AI-Assisted Prediction and Reflection	Demonstrates critical comparison between AI and human predictions; identifies biases, limitations, and data-driven corrections.	Compares AI and human results with moderate reflection on discrepancies.	Summarizes AI output with limited critical analysis.	Relies solely on AI output without evaluation or justification.		

Criteria	Excellent (4)	Proficient (3)	Developing (2)	Beginning (1)	Grade	Feedback
QA/QC Plan Design	Proposes comprehensive, realistic calibration and control plan with appropriate standards, acceptance criteria, and verification steps.	Includes key QA/QC elements but lacks one or two important components.	Basic plan with general statements; lacks measurable acceptance criteria.	Incomplete or unrealistic QA/QC plan.		
Pre-registration of Identity Hypothesis	Develops logical, data-based hypotheses with clear decision criteria and decisive technique justification.	Provides reasonable hypotheses with limited rationale.	Hypotheses vague or missing decisive technique justification.	No clear hypothesis or justification.		
Conceptual Understanding (Mini Prelab Quiz)	Accurately answers all conceptual questions with clear, scientifically sound reasoning.	Minor inaccuracies; overall demonstrates understanding.	Some correct concepts but with notable misconceptions.	Major misconceptions or incomplete answers.		
Scientific Communication and Clarity	Responses are precise, well-organized, and use appropriate scientific terminology.	Generally clear and well-structured with minor lapses in terminology.	Disorganized or lacks technical precision.	Incoherent, minimal, or off-topic responses.		

Scoring and Weighting (Total = 100%)

Category	Weight
Identification & Instrumental Prediction (Criteria 1–2)	25%
Predictive Analysis & Table (Criteria 3–4)	20%
AI Integration & Reflection (Criterion 5)	15%
QA/QC Plan & Hypothesis (Criteria 6–7)	20%
Conceptual Quiz & Communication (Criteria 8–9)	20%

Performance Interpretation:

- 90–100 (Excellent): Mastery of predictive reasoning and interdisciplinary integration.
- 75–89 (Proficient): Solid conceptual understanding and methodological awareness.
- 60–74 (Developing): Partial understanding; reinforcement needed.
- <60 (Beginning): Lacks conceptual and procedural understanding.

Rubric For Evaluating the Results Integration Sheet – Ferrocenyl Chalcones

This rubric is designed to assess students' ability to synthesize, interpret, and integrate data obtained from multiple instrumental analytical techniques (UV-Vis, Fluorescence, FTIR, NMR, HPLC, DSC, TGA) into a coherent and scientifically justified conclusion regarding compound identity, purity, and quality control.

Criterion	Exemplary (4)	Proficient (3)	Developing (2)	Beginning (1)	Weight	Grade	Feedback
Completeness and Accuracy of Experimental Data	All techniques are included; data are accurately transcribed, units correct, and calibration verified; clear understanding of instrument outputs.	Most data reported with minor errors or omissions; calibration partially verified.	Some techniques missing or contain significant errors; limited calibration discussion.	Data incomplete or inaccurate; minimal understanding of experimental outcomes.	15%		
Integration Across Techniques	Exceptional ability to interconnect results from spectroscopy, chromatography, and thermal analysis; clear correlations.	Integrates most techniques logically; minor inconsistencies.	Limited integration; superficial or lacks chemical rationale.	No integration; techniques reported in isolation.	20%		
Chemical Interpretation and Identity Assignment	Correctly identifies chalcone; provides specific spectral or chromatographic evidence supporting identification.	Identifies compound with reasonable justification; minor misinterpretations.	Identification uncertain or weakly supported by data.	Incorrect or unjustified identification.	20%		
Purity Assessment and Critical Comparison (HPLC, DSC, NMR)	Accurately interprets purity data; correlates findings; explains discrepancies using chemical reasoning.	Adequate purity analysis; some discussion of discrepancies.	Purity results partially analyzed; lacks comparison.	Misinterprets or omits purity analysis.	15%		
Consistency and Cross-validation	Deep understanding of reinforcing/challenging results; example of	General consistency analysis; at least one	Mentions consistency superficially.	No discussion of consistency.	10%		

Criterion	Exemplary (4)	Proficient (3)	Developing (2)	Beginning (1)	Weight	Grade	Feedback
Among Techniques	match/discrepancy with explanation.	match/discrepancy discussed.					
Application of GLP and QA/QC Principles	Clearly describes calibration standards (caffeine, $K_2Cr_2O_7$, polystyrene, indium) and explains how they ensure reliability.	Mentions calibration and QA/QC; limited reflection.	References calibration without connecting it to validity.	No mention of calibration or QA/QC.	10%		
Integrative Conclusion and Reflection	Synthesizes all results into evidence-based conclusion; identifies most decisive technique with rationale.	Provides reasonable summary with some integration.	Summarizes without linking data; unclear rationale.	No clear conclusion; lacks justification.	10%		

Scoring and Feedback Guide

Score Range	Performance Level	Descriptor
31–28	Outstanding (A)	Analytical, cohesive, and demonstrates mastery of instrumental interpretation and reasoning.
27–23	Proficient (B)	Solid understanding; minor gaps in integration or interpretation.
22–18	Developing (C)	Basic completion with limited synthesis; mostly descriptive.
17–10	Beginning (D/F)	Incomplete, inconsistent, or incorrect data; lacks analytical understanding.

Instructor Evaluation Notes

- Provide qualitative feedback on data integrity, calibration, and interpretation.
- Highlight evidence-based reasoning (referencing peaks, shifts, chromatographic behavior).
- Encourage precise, data-driven writing.
- Use alongside the Technical Data Sheet and ACS-format report for comprehensive assessment.

Rubric For Evaluating the Laboratory Report – Characterization and Quality Control of Ferrocenyl Chalcones

This rubric evaluates students' ability to write a professional scientific report in ACS format, demonstrating mastery in data analysis, interpretation, synthesis of instrumental results, and effective scientific communication. The report should integrate data from UV-Vis, Fluorescence, FTIR, NMR, HPLC, DSC, and TGA analyses.

Criterion	Exemplary (4)	Proficient (3)	Developing (2)	Beginning (1)	Weight	Feedback	Grade
Title and Abstract	Title concise and informative; abstract (150–200 words) clearly states objectives, methods, main findings, and conclusions using scientific language.	Title appropriate; abstract summarizes key aspects with minor omissions.	Title or abstract lacks precision; some critical elements missing or unclear.	Title vague or missing; abstract incomplete or off-topic.	10%		
Introduction and Theoretical Context	Provides a well-developed background explaining ferrocenyl chalcones' relevance, instrumental techniques, and purpose; integrates literature and chemical theory.	Presents relevant background and rationale; some theoretical details missing.	Minimal context or theory; lacks logical connection between background and objectives.	Lacks context or inaccurate introduction.	15%		
Experimental Methodology (Summarized)	Concise ACS-style description; includes calibration, instruments, and safety considerations; demonstrates GLP and QA/QC understanding.	Adequate description; includes most relevant details; minor omissions.	Procedural description without rationale or calibration info.	Incomplete or copied methods; lacks understanding.	10%		

Criterion	Exemplary (4)	Proficient (3)	Developing (2)	Beginning (1)	Weight	Feedback	Grade
Results Presentation (Tables, Figures, Graphs)	Data clear and organized; figures/tables numbered, captioned, referenced; high-quality visuals showing trends and comparisons.	Data mostly clear and formatted correctly; minor inconsistencies.	Results lack clarity or labeling; formatting issues.	Results incomplete, disorganized, or missing visuals.	10%		
Data Interpretation and Discussion	Insightful interpretation supported by evidence (peaks, λmax, R^2, Tm, etc.); integrates multiple techniques coherently; discusses anomalies scientifically.	Adequate interpretation; discusses trends and findings with limited cross-analysis.	Descriptive rather than analytical; weak interpretation.	No meaningful interpretation; lists results only.	20%		
Comparison with Literature and AI Predictions	Thoroughly compares experimental data with literature and AI predictions; identifies patterns and discrepancies with critical reasoning.	Includes comparisons with literature values; limited discussion.	Mentions literature without analysis or relevance.	No or incorrect literature comparison.	10%		
Discussion of Purity, Structure, and Identity	Explains identity and purity using multiple evidences (HPLC, NMR, FTIR, DSC, TGA); provides scientific rationale and validation.	Explains identity/purity adequately; minor gaps in validation.	Partial discussion; misses key correlations.	Lacks justification or incorrect analysis.	10%		

Criterion	Exemplary (4)	Proficient (3)	Developing (2)	Beginning (1)	Weight	Feedback	Grade
Reflection on GLP and QA/QC	Discusses standards/calibrations (caffeine, $K_2Cr_2O_7$, indium, polystyrene) and connects to data reliability; demonstrates ethical awareness.	Mentions GLP/QA-QC with limited discussion.	References calibration superficially without analytical link.	No mention of GLP or QA/QC.	5%		
Conclusions	Concise, evidence-based, aligned with results; identifies key findings, technique relevance, and limitations; includes reflection on learning outcomes.	Clear summary of findings; minor omissions.	Generic or weak conclusions; lacks linkage to results.	Missing or unsupported conclusions.	5%		
Writing Quality and ACS Formatting	Writing fluent, precise, and professional; follows ACS format; no grammatical errors.	Minor errors; mostly consistent with ACS style and tone.	Several grammar/formatting issues; inconsistent writing.	Poor writing; lacks structure or ACS compliance.	5%		

Scoring and Feedback Guide

Score Range	Performance Level	Descriptor	Instructor Evaluation Notes:
36–40	Outstanding (A)	Excellent integration of data and reasoning; professional ACS-style report.	- Provide written Feedback on data integrity, interpretation logic, and scientific coherence.<br>- Highlight connections among techniques and reasoning quality.<br>- Evaluate ACS-format compliance (titles, captions, references, structure).<br>- Encourage reflection on calibration, safety, and quality assurance.
31–35	Proficient (B)	Solid and coherent report; minor weaknesses.	
25–30	Developing (C)	Adequate completion with limited synthesis or weak writing.	
<25	Beginning (D/F)	Incomplete or incorrect; lacks analytical understanding and structure.	
